# Role of OAF and smoking initiation in COPD risk: Insights from Mendelian randomization

**DOI:** 10.18332/tid/210379

**Published:** 2025-10-09

**Authors:** Feng You, Kai Xu, Gengzhong Chen, Siwen Chen, Qiheng Yuan, Bianjin Sun

**Affiliations:** 1 The Wenzhou Third Clinical Institute Affiliated to Wenzhou Medical University, Wenzhou People's Hospital Affiliated to Hangzhou Medical College, Wenzhou People's Hospital, Wenzhou Maternal and Child Health Care Hospital, Wenzhou, China; 2 National Clinical Research Center for Ocular Diseases, Eye Hospital, Wenzhou Medical University, Wenzhou, China; 3 Wenzhou Key Laboratory of Sanitary Microbiology, Key Laboratory of Laboratory Medicine, Ministry of Education, School of Laboratory Medicine and Life Science, Wenzhou Medical University, Wenzhou, China

**Keywords:** chronic obstructive pulmonary disease, out at first, smoking initiation, immune cell phenotypes, mendelian randomization

## Abstract

**INTRODUCTION:**

Chronic Obstructive Pulmonary Disease (COPD) is a complex condition influenced by both genetic and environmental factors. This study aims to identify potential biomarkers and explore the associations between specific maker genes, smoking initiation, and COPD.

**METHODS:**

We used Mendelian randomization (MR) with inverse variance weighting (IVW) to identify significant associations (p<0.05). Genetic instruments for smoking initiation (Genome-Wide Association Study [GWAS]: ieu-b-4877) and Out at First (OAF) (cis-expression Quantitative Trait Locus [eQTL] GWAS) were selected based on single nucleotide polymorphisms (SNPs) with p<5×10^-8^. COPD GWAS data served as the outcome. Two-sample MR analysis estimated causal effects of smoking initiation/OAF on COPD. Mediation analysis explored the role of 731 immune cell phenotypes in these associations.

**RESULTS:**

We identified OAF as a key genetic marker associated with COPD risk, with the eQTL analysis yielding an odds ratio (OR) of 1.09 (95% CI: 1.02–1.17, p=0.01). The IVW analysis for smoking initiation-COPD indicated an OR of 1.89 (95% CI: 1.69–2.11, p<0.001). Mediation analysis revealed that the immune cell phenotype CD14- CD16- absolute count (AC) (GCST90001581) accounted for 30.16% and 4.27% of the mediation in the OAF-COPD and smoking initiation-COPD associations, respectively.

**CONCLUSIONS:**

The MR results suggest that OAF may be a genetic risk factor for COPD, with immune cell phenotypes, particularly CD14-CD16-AC, potentially playing a mediating role in COPD development. Smoking initiation is also positively correlated with COPD, playing an important role in its pathogenesis.

## INTRODUCTION

COPD is a typical long-standing respiratory ailment characterized by a gradual reduction in airflow, which is usually not fully reversible^1^. It is often associated with airway inflammation and a range of respiratory symptoms. The pathogenesis of COPD is complex, involving both genetic predisposition and environmental factors. Long-term exposure to tobacco smoke is the primary cause of COPD^2,3^. With the accelerating process of global population aging and the increasing severity of environmental pollution, the prevention and control of COPD have become more challenging, requiring greater attention and concerted efforts from all sectors of society. Although the basic pathological processes of COPD are better understood, identifying molecular features for early diagnosis, as well as exploring the potential effects of smoking on COPD, remains a major challenge.

It is well established that smoking is harmful to lung health. Factors such as the age at which smoking begins, the frequency and intensity of smoking, and smoking status (current smoker, passive smoker, or former smoker) may all play significant roles in determining the risk of COPD. Therefore, addressing smoking-related issues is of paramount importance. Studies have shown that maternal smoking around birth increases the risk of COPD in offspring^4^. In certain young smokers, imaging abnormalities, such as ground-glass opacities and functional small airway disease, occur prior to detectable changes in lung function^5^. Smoking initiation may also have long-term adverse effects on lung function, which still warrants further investigation.

The pathogenesis of COPD involves various immune cells, including macrophages, lymphocytes, and neutrophils^6^. NK cells are crucial innate immune cells that are primarily responsible for directly recognizing and eliminating virus-infected and tumor cells. Similar to peripheral blood NK cells, those in lung tissue are also lymphocytes, accounting for 5% to 20% of the total lymphocyte population in the lungs^7^. These NK cells are typically composed of three subtypes, with approximately 80% being circulating cells of the CD56dimCD16+ population^7^. NK cells are also important in the pathological progression of various pulmonary diseases, including lung cancer, acute lung injury, and COPD^8,9^.

MR is a key method for understanding genetic determinants and identifying potential therapeutic targets. Cis-protein Quantitative Trait Loci (cis-pQTLs) are genetic variants that impact the abundance of plasma proteins, which in turn affect protein function and stability, contributing to the onset and development of diseases^10,11^. On the other hand, cis-expression Quantitative Trait Loci (cis-eQTLs) are genetic variations located near the target gene that regulate gene expression levels, thereby influencing related biological processes^11,12^. Compared to trans-eQTL and trans-pQTL, cis-eQTL and cis-pQTL exhibit greater specificity, as they generally affect genes that are either adjacent or located within the same genomic region. This specificity minimizes interference from other genes or regulatory elements, thereby enhancing the study’s reliability.

In this finding, we identified potential gene markers for COPD by analyzing NK cell gene expression at the single-cell level. We then employed a two-sample MR approach to validate these biomarkers. Additionally, incorporating mediating variables to explore their potential mediating effects in the MR analysis of smoking initiation-COPD and the characteristic gene-COPD.

## METHODS

### Study design

We used MR to analyze the impact of smoking initiation and genetic factors on COPD risk. First, candidate genes were identified and validated using multiple approaches. Specifically, we analyzed single-cell RNA-seq data related to COPD, performing dimensionality reduction and clustering to identify distinct cell types, followed by differential expression analysis between COPD and control groups to extract NK cell marker genes.

Next, we conducted comprehensive screening and validation of candidate genes using cis-eQTLs and cis-pQTLs. Two-sample MR analyses were then performed to evaluate the causal effects of smoking initiation and genetic factors on COPD, including the assessment of potential mediating effects of 731 immune cell phenotypes.

### Source and handling of scRNA-seq data

The scRNA-seq data related to COPD (GSE270667) were downloaded from the Gene Expression Omnibus (GEO) database, consisting of three cases of COPD (GOLD grade I) and three matched control lung tissue samples. Normalization and scaling were performed to ensure comparability across cells. The samples from the disease group and the control group were merged separately, and linear dimensionality reduction was performed using principal component analysis (PCA). Subsequently, cell clustering was performed, followed by nonlinear dimensionality reduction using t-distributed stochastic neighbor embedding (t-SNE). Differential analysis was conducted to identify marker genes, with an absolute value of the average log2 fold change (avg_log2FC) greater than 1 and an adjusted p-value (FDR) less than 0.05. We extracted the marker genes expressed by NK cells for subsequent analysis. The analysis utilized R packages such as *limma*, *Seurat*, *dplyr*, *SingleR*, and *celldex*.

### MR analysis

This study was conducted according to predefined inclusion criteria and underwent quality assessment using the STROBE-MR guidelines^13^. The checklist can be found in the Supplementary file. We illustrated the principles of Mendelian randomization using Figdraw (Supplementary file Figure S1).

In the MR analysis, we utilized GWAS data for COPD, which included 21617 cases and 372627 controls sourced from the FinnGen consortium (https://r11.finngen.fi/). The GWAS data on smoking initiation (ieu-b-4877) were downloaded from the IEU database, with a sample size of n=607291. The eQTL data came from the eQTLGen Consortium. Additionally, the proteomic data comprised 4907 plasma proteins from 35559 individuals in Iceland^14^. The genetic data for 731 immune cell phenotypes were obtained from European populations and include various trait types, such as relative count, absolute count, mean fluorescence intensity (MFI), and morphological parameters^15^. These data are associated with GWAS Catalog Accession Numbers GCST90001391 to GCST90002121. The corresponding trait types for each GWAS ID are provided in Supplementary file Table S1.

The clumping parameters were set as follows: cis_wind_kb=1000 kb, p=5×10^-8^, clump_kb=10000 kb, and r^2^=0.1. Five MR methods were used, with IVW as the main approach for evaluating the causal effect. Heterogeneity analysis was conducted using the MR-Egger and IVW methods, with MR-Egger applied to evaluate pleiotropy. The R packages used included ‘TxDb.Hsapiens.UCSC.hg38.knownGene’, ‘TwoSampleMR’, *ggplot2*, and *foreach*.

## RESULTS

### Differentially expressed genes specific to NK cells in COPD tissue

**Figure 1:**
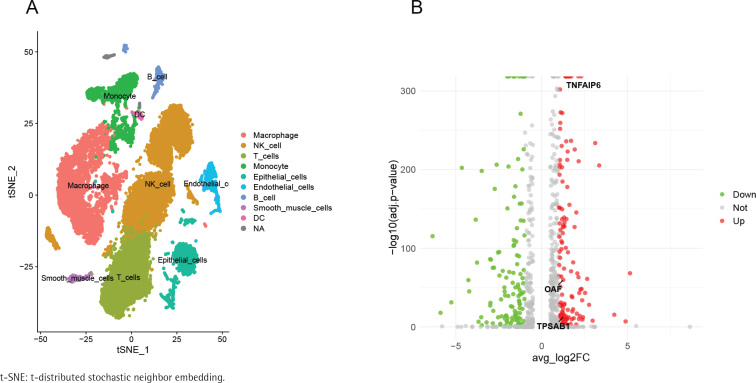
Single-cell t-SNE maps and volcano plots of differentially expressed genes: A) t-SNE plot of single cells, showing classified cell clusters in distinct colors. Axes tSNE_1 and tSNE_2 represent the two-dimensional embedding of high-dimensional gene expression, highlighting the distribution and heterogeneity of cell populations; B) Volcano plot of differentially expressed genes in NK cells between the COPD and control groups, highlighting significantly up- and down-regulated genes

This study aimed to explore cell type-specific gene enrichment in COPD tissue from a cellular perspective, using scRNA-seq data (GSE270667) from the GEO database. The cells were annotated and classified into nine distinct types, including macrophages, NK cells, monocytes, epithelial cells, T cells, B cells, endothelial cells, smooth muscle cells, dendritic cells (DC), and an unclassified cell population (Figure 1A). Among these, we focused on NK cells.

We performed differential expression analysis to compare gene expression levels in NK cells between the COPD and control groups. After filtering based on an absolute avg_log2FC greater than 1 and an adjusted p<0.05, we identified 253 differentially expressed genes (DEGs) (Supplementary file Table S2). The volcano plot (Figure 1B) visualizes these results, with green representing downregulated genes in the disease group, grey for non-significant genes, and red for upregulated genes.

### Significant role of OAF in COPD risk

We identified 15695 cis-eQTLs using cis-filtering criteria, and after intersecting these with the 253 DEGs, 148 common genes were found. The eQTLs of these 148 genes were used as exposure instruments in a two-sample MR analysis with COPD GWAS data. This analysis identified 25 positive results (IVW method, p<0.05), including genes like AURKB, BAMBI, CLEC10A, OAF, TNFAIP6, and TPSAB1 (Table 1).

**Table 1:** Results of MR analysis using the IVW method based on cis-eQTL data

*Exposure*	*Outcome*	*SNPs*	*OR (95% CI)*	*p*	*Heterogeneity*	*Pleiotropy*
AURKB	COPD	6	0.93 (0.87–0.99)	0.02	0.46	0.40
BAMBI	COPD	15	0.93 (0.87–0.99)	0.02	0.20	0.03
CLEC10A	COPD	16	1.07 (1.02–1.13)	0.01	0.04	0.15
DSP	COPD	52	1.04 (1.02–1.06)	<0.001	0.26	0.18
HSPG2	COPD	16	0.97 (0.94–1.00)	0.04	0.22	0.71
IGHV4-59	COPD	5	1.09 (1.00–1.2)	0.05	0.60	0.25
LCN2	COPD	3	0.90 (0.81–0.99)	0.03	0.36	0.59
LDB2	COPD	9	1.17 (1.06–1.28)	<0.01	0.78	0.84
NAPSA	COPD	6	0.90 (0.84–0.97)	<0.01	0.80	0.87
OAF	COPD	10	1.09 (1.02–1.17)	0.01	0.11	0.60
PKIB	COPD	15	0.95 (0.90–1.00)	0.05	0.47	0.57
PLEKHH2	COPD	34	1.08 (1.03–1.12)	<0.001	0.19	0.92
PPL	COPD	15	1.13 (1.05–1.22)	<0.01	0.23	0.39
S100B	COPD	42	1.03 (1.01–1.04)	<0.001	0.17	0.03
SEMA3C	COPD	10	0.92 (0.87–0.98)	0.01	0.56	0.56
TCN2	COPD	43	1.05 (1.01–1.08)	0.01	0.03	0.11
THBS1	COPD	38	0.97 (0.94–0.99)	0.01	0.93	0.69
TMEM45A	COPD	21	1.04 (1.00–1.09)	0.04	0.45	0.52
TNFAIP6	COPD	27	0.93 (0.89–0.96)	<0.001	0.78	0.60
TNFRSF10D	COPD	7	1.18 (1.05–1.32)	<0.01	0.28	0.87
TPPP3	COPD	7	0.87 (0.79–0.95)	<0.01	0.93	0.88
TPSAB1	COPD	32	0.93 (0.91–0.96)	<0.001	0.18	0.04
TRAV8-4	COPD	6	0.92 (0.88–0.97)	<0.01	0.81	0.76
VEGFA	COPD	12	1.07 (1.01–1.13)	0.02	0.52	0.56
ZDHHC1	COPD	4	0.86 (0.79–0.94)	<0.001	0.54	0.31

IVW: inverse variance weighted. eQTL: expression quantitative trait loci. COPD: chronic obstructive pulmonary disease. SNPs: single-nucleotide polymorphisms. The ‘Heterogeneity’ column reports the p-value from Cochran’s Q statistic (derived from the IVW method). The ‘Pleiotropy’ column reports the p-value from the MR-Egger intercept test.

Next, we selected 1614 cis-pQTLs and intersected them with the 25 identified genes, finding 12 cis-pQTLs for 9 plasma proteins. Using these cis-pQTLs as exposure SNPs in two-sample MR analysis with COPD GWAS data, we identified three positive results, suggesting a significant role of these proteins in COPD risk (Table 2).

**Table 2:** Results of MR analysis using the IVW method based on cis-pQTL data

*Exposure*	*Outcome*	*SNPs*	*OR (95% CI)*	*p*	*Heterogeneity*	*Pleiotropy*
OAF	COPD	79	1.03 (1.00–1.05)	0.04	0.87	0.22
TNFAIP6	COPD	85	0.96 (0.94–0.98)	<0.001	0.23	0.69
TPSAB1	COPD	152	0.98 (0.97–1.00)	0.01	0.45	0.73

IVW: inverse variance weighted. pQTL: protein quantitative trait loci. COPD: chronic obstructive pulmonary disease. SNPs: single-nucleotide polymorphisms. The ‘Heterogeneity’ column reports the p-value from Cochran’s Q statistic (derived from the IVW method). The ‘Pleiotropy’ column reports the p-value from the MR-Egger intercept test.

**Figure 2:**
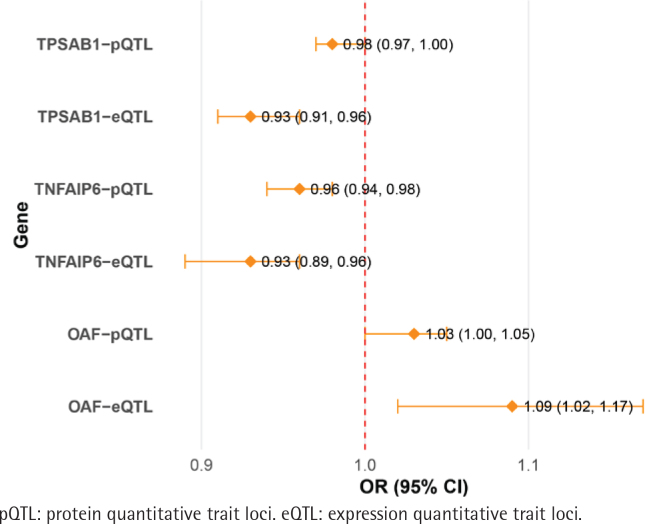
Forest plot showing odds ratio (OR) for genetic variants from eQTL and pQTL analyses

Figure 2 presents the results of the MR analysis using the IVW method. The eQTL OR values of the three potential marker genes align with those observed in the pQTL analysis. OAF has an OR greater than 1, while TNFAIP6 and TPSAB1 have ORs less than 1. Additionally, Figure 1B demonstrates the upregulation of these genes in the COPD group. As a result, TNFAIP6 and TPSAB1 were excluded from further analysis, with the focus shifting to OAF in relation to COPD development. Specifically, the MR results for the OAF-COPD association revealed that the eQTL analysis yielded an odds ratio (OR) of 1.09 (95% CI: 1.02–1.17, p=0.01), while the pQTL analysis showed an OR of 1.03 (95% CI: 1.00–1.05, p=0.04).

**Figure 3:**
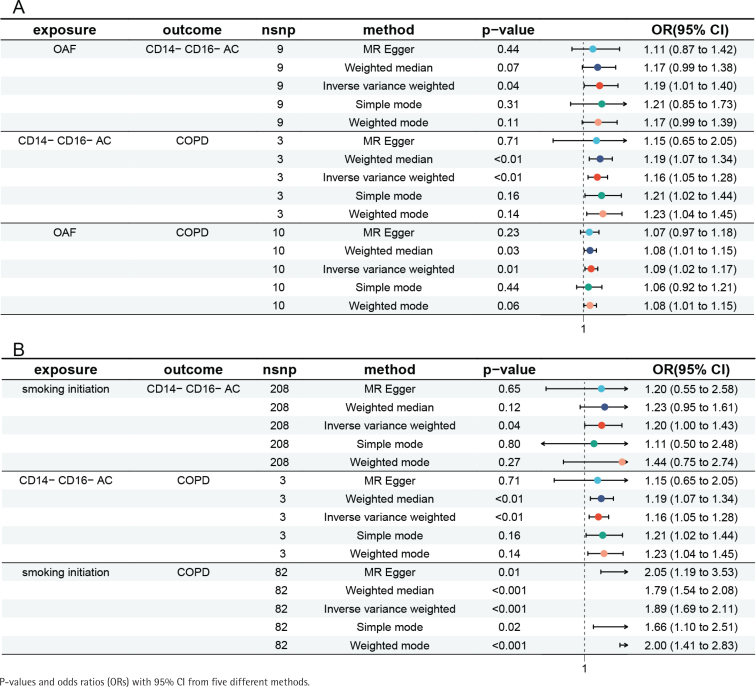
Forest plot of MR analysis: A) Examining the association between OAF, immune cell phenotype, and COPD; B) Examining the association between smoking initiation, immune cell phenotype, and COPD

The OAF-COPD MR results from either the eQTL or pQTL perspectives, showed no significant evidence of horizontal pleiotropy (MR-Egger intercept=0.005, p=0.60) or heterogeneity (Cochran’s Q=14.43, p=0.11). These results support the validity of the instrument strength independent of direct effect (InSIDE) assumption for our MR models.

Additionally, based on single-cell data, we found that OAF is not only expressed in NK cells but also in other cell types, such as smooth muscle cells and endothelial cells. (Supplementary file Figure S2) illustrates the expression differences of the OAF gene across various cell types.

### Significant role of smoking initiation in COPD risk

Mendelian randomization analysis was performed with smoking initiation as the exposure variable and COPD as the outcome. SNPs strongly associated with smoking initiation were selected based on a threshold of p<5×10^-8^. The initial IVW results showed an OR of 1.84 (95% CI: 1.62–2.08, p<0.001). However, heterogeneity analysis (using both MR Egger and IVW methods) revealed p<0.001, prompting the exclusion of three SNPs – rs66631011, rs1565735, and rs2274993 – based on the leave-one-out sensitivity analysis and forest plots. After excluding these SNPs and re-running the MR analysis, the IVW results showed an OR of 1.89 (95% CI: 1.69–2.11, p<0.001), with the conclusion remaining unchanged. Additionally, no heterogeneity or pleiotropy was observed in the MR results (Table 3) (p>0.05).

**Table 3:** Detailed results of MR sensitivity analyses

*Exposure*	*Outcome*	*MR-Egger Intercept*	*Intercept p*	*Cochran’s Q*	*Q p*
OAF (eQTL)	COPD	0.005	0.60	14.43	0.11
OAF (pQTL)	COPD	-0.004	0.22	64.17	0.87
CD14−CD16−AC	COPD	0.002	0.98	2.29	0.32
OAF	CD14−CD16−AC	0.015	0.48	9.33	0.32
Smoking initiation	COPD	-0.002	0.77	101.19	0.06
Smoking initiation	CD14−CD16−AC	7.37E-05	0.99	221.24	0.22

OAF: out at first. COPD: chronic obstructive pulmonary disease. AC: absolute count. eQTL: expression quantitative trait loci. pQTL: protein quantitative trait loci. Cochran’s Q statistic and its corresponding p-value were derived from the IVW method.

### Causal impact of 731 immune cell phenotypes on COPD

We performed a genetic association analysis aimed at identifying single nucleotide polymorphisms (SNPs) that are linked to 731 distinct immune cell phenotypes. To ensure robustness in our findings, we set a significance threshold at p<5×10^-8^. We employed a genomic window of 10000 kilobases and established an r^2^ threshold of 0.1 to refine our search for associations. Subsequently, a two-sample MR analysis was performed, and based on the results from the IVW method, 92 immune cell phenotypes with adjusted p<0.05 were identified as potentially causally related to COPD (Supplementary file Table S3). For example, immune cell phenotypes related to relative count included IgD- CD27- %B cells, HLA DR+ T cell %lymphocyte, HLA DR+ NK %NK, HLA DR+ NK %CD3- lymphocyte and IgD- CD27- %lymphocytes; those associated with absolute count included CD62L- monocyte AC, DC AC, myeloid DC AC, CD33- HLA-DR+ AC, and CD62L- DC AC. Regarding mean fluorescence intensity (MFI), we identified BAFF-R on IgD- CD38-, BAFF-R on IgD- CD27-, and BAFF-R on IgD- CD24-. In terms of morphological parameters, FSC-A on HLA-DR+ NK cells was also notable.

### Mediated Mendelian randomization analysis

Based on the 92 immune cell phenotypes identified, we performed a two-step MR analysis to investigate how immune cell phenotypes mediate the relationships between OAF and COPD, as well as between smoking initiation and COPD. As shown in Supplementary file Figure S1, in the first step, we used SNPs strongly associated with OAF (p<5×10^-8^) or smoking initiation (p<5×10^-6^) as exposures, with 92 immune cell phenotypes as outcomes. The MR analysis estimated the causal effect of these exposures on immune cell phenotypes, denoted as β1. In the second step, we treated the identified immune cell phenotypes as exposures and COPD as the outcome, evaluating the causal relationship between immune cell phenotypes and COPD, with the effect size denoted as β2. The total, mediation, and mediation proportion were then calculated to assess the role of immune cell phenotypes in mediating the OAF-COPD and smoking initiation-COPD connections.

Interestingly, we found that the mediation proportion of CD14-CD16- AC (GCST90001581) in mediating the OAF-COPD and smoking initiation-COPD relationships was the largest, at 30.16% and 4.27%, respectively (Supplementary file Tables S4 and S5). The detailed MR results for the causal relationships between OAF, CD14-CD16- AC, and COPD are shown in Figure 3A, while those for smoking initiation, CD14-CD16- AC, and COPD are presented in Figure 3B.

**Figure 4:**
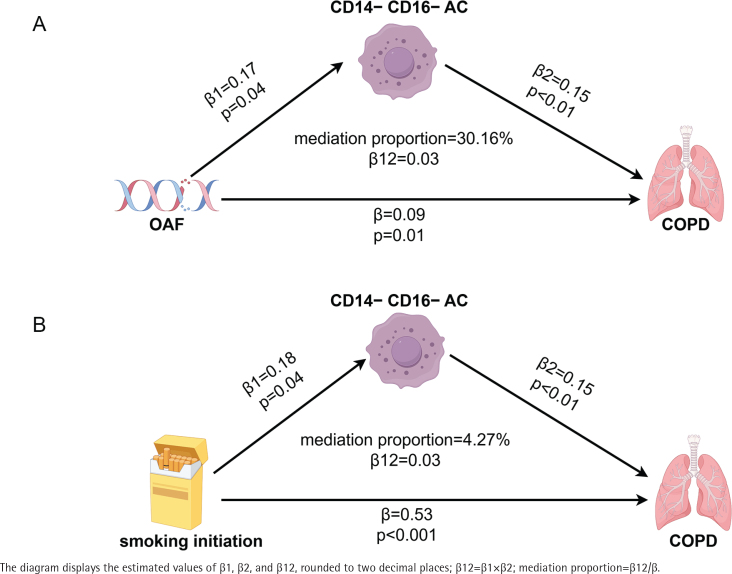
Visualization of the results from the mediation MR analysis: A) Association between OAF, CD14-CD16-AC, and COPD; B) Association between smoking initiation, CD14-CD16-AC, and COPD

Figure 4, created using Figdraw, illustrates the mediation MR results. As shown in Figure 4A, the data suggest that OAF may be a risk factor for COPD (β=0.09), with OAF positively associated with CD14-CD16-AC (β1=0.17), and CD14-CD16-AC positively associated with COPD (β2=0.15). Figure 4B suggests a positive correlation between smoking initiation and COPD (β=0.53), with a potential mediation effect through CD14-CD16-AC, although the mediation proportion is relatively small at 4.27%. No pleiotropy or heterogeneity was detected (Table 3) (p>0.05).

Furthermore, we analyzed the SNPs (p<5×10^-8^) strongly associated with both OAF (cis-eQTL) and smoking initiation, and found no overlap between the SNPs linked to these two exposures.

## DISCUSSION

COPD is a significant chronic disease globally and warrants widespread attention. According to the MR analysis, OAF may be a potential risk factor for COPD (β=0.09), with a positive correlation observed between OAF and CD14-CD16-AC (β1=0.17), and between CD14-CD16-AC and COPD (β2=0.15). In contrast, the MR analysis of smoking initiation and COPD revealed a stronger correlation (β=0.53), suggesting that smoking initiation has a more direct impact on COPD. Although a mediation effect through immune cell phenotypes was identified, its proportion was low (4.27%), indicating that smoking initiation likely affects COPD through direct mechanisms, with immune system activation playing a supplementary role. The stronger effect of smoking initiation further underscores the importance of addressing smoking as a primary risk factor for COPD. These findings highlight the urgent need for ongoing smoking cessation efforts to reduce the burden of COPD.

The OAF (Out at First) protein was first described in Drosophila and is primarily associated with neuronal development^16^. Currently, research on human OAF in diseases is quite limited. Recent studies have revealed substantial structural likenesses between OAF and various members of the BRICHOS family members, suggesting that OAF might be a new addition to the human BRICHOS family^17^. Furthermore, OAF shows potential as a novel biomarker for pulmonary tuberculosis (TB)^18^. All members of the human BRICHOS family contain a BRICHOS domain, thought to act as a chaperone protein, assisting in the folding and stability of other proteins. The human BRICHOS family comprises ten well-known proteins, which are classified into five subfamilies: proSP-C/SFTPC, ITM/BRI, BRICD5, Gastrokines, and Tenomodulin/Chondromodulin. Various diseases, including neurodegenerative disorders, respiratory distress, and cancer, are closely connected to members of the BRICHOS family^19-21^. For example, mutations in BRI2 are associated with a variety of neurodegenerative diseases^22-24^, while abnormal expression of CA11 is associated with stomach cancer^25^.

Interestingly, proSP-C (pro-Surfactant Protein C), part of the BRICHOS family, is a crucial protein synthesized in the alveoli and is closely associated with various respiratory diseases, particularly chronic inflammatory lung diseases. Alterations in the SFTPC gene are regarded as a major cause of interstitial lung disease. These mutations may disrupt the synthesis of proSP-C, thereby impairing the normal function of surfactant proteins and ultimately leading to pathological changes such as lung inflammation and fibrosis^26,27^. Additionally, proSP-C can affect the gas exchange function of the alveoli, influencing the progression of interstitial lung disease^28^. In our study, based on Mendelian results, OAF exhibited an OR greater than 1, suggesting its role in promoting the development of COPD.

CD14 and CD16 are key markers for monocyte subpopulations, and based on their expression profiles, monocytes are classified into three subtypes. Specifically, CD14++CD16- (classical monocytes) account for approximately 80% of the population and are characterized by strong phagocytic activity. CD14dimCD16+ (non-classical monocytes) typically make up 10–15% of the population and are mainly involved in the clearance of damaged cells and regulation of inflammatory responses. CD14+CD16+ (intermediate monocytes) represent around 5–10% of monocytes and are considered to play an important role in inflammation and immune responses^29,30^. In our study, the CD14- CD16- subpopulation (GCST90001581) refers to a specific group identified within the monocyte panel, which does not express CD14 or CD16 on their surface. Research on this subpopulation is limited, but some studies have reported that these cells consist of dendritic cells (DCs) and lymphocytes^31^.

The involvement of DCs and lymphocytes, notably CD8+ cytotoxic T cells, is vital in COPD pathogenesis. DCs recognize and capture pathogens and harmful substances induced by smoking or other environmental factors, migrate to lymph nodes, and present antigens, thereby activating and promoting the proliferation and expansion of specific lymphocytes, particularly CD8+ T cells^32,33^. In addition to antigen presentation, DCs secrete several pro-inflammatory cytokines, including IL-6, IL-12, etc. Studies have shown that the number of DCs is significantly increased in the lungs of COPD patients, which is likely associated with prolonged smoking and ongoing environmental stimuli^34^. Moreover, the cytokines secreted by DCs not only promote lymphocyte proliferation but also regulate their function. For instance, dendritic cells secrete IL-12, which enhances the cytotoxic activity of CD8+ T cells^35,36^.

### Strengths and limitations

This study has several notable strengths. We approached the identification of candidate genes associated with COPD from a micro-level perspective, specifically at the single-cell level, and validated our findings across multiple dimensions. We further analyzed and discussed the associations between a smoking-related variable (smoking initiation), a non-smoking variable (OAF), and COPD risk, incorporating immune cell phenotypes to explore potential mediating effects. Moreover, we utilized the most recent data from the Finnish cohort (R11), enhancing the robustness and generalizability of our conclusions.

However, this study has several limitations. While we focused on COPD as a whole, we did not investigate the specific relationships between the identified gene and COPD subtypes. The single-cell analysis in this study was based on only 3 COPD samples and 3 control samples. The small sample size may limit the generalizability and statistical power of the results, potentially overlooking key characteristic genes and being susceptible to sample bias. And future studies should include larger sample sizes to validate these preliminary findings. Additionally, both the smoking initiation and COPD cohorts were of European descent, which may limit the generalizability of our findings to other populations. Furthermore, the smoking initiation data may be subject to recall bias, as participants may have misreported the actual initiation time due to memory recall issues. In the MR analysis involving immune cell phenotypes and COPD, the number of SNPs included was relatively small, and the conclusions may require further validation. Additionally, this study only examined immune cell phenotypes as potential mediators, without exploring other possible mediating factors, such as plasma metabolites.

## CONCLUSIONS

Taken together, OAF may be a genetic risk factor for COPD, with immune cell phenotypes, particularly CD14-CD16- AC, potentially mediating its development. The positive link between smoking initiation and COPD highlights the need for early interventions.

## Supplementary Material



## Data Availability

The data supporting this research are available from the authors on reasonable request.
