# Supplementary material for: Role of OAF and smoking initiation in COPD risk: Insights from Mendelian randomization
Source: Tob Induc Dis. 2025 Oct 9;23:10.18332/tid/210379. doi: 10.18332/tid/210379 (PMC12510324; doi:10.18332/tid/210379)
Supplement: Supplementary file 1 [file TID-23-151-s1.pdf]

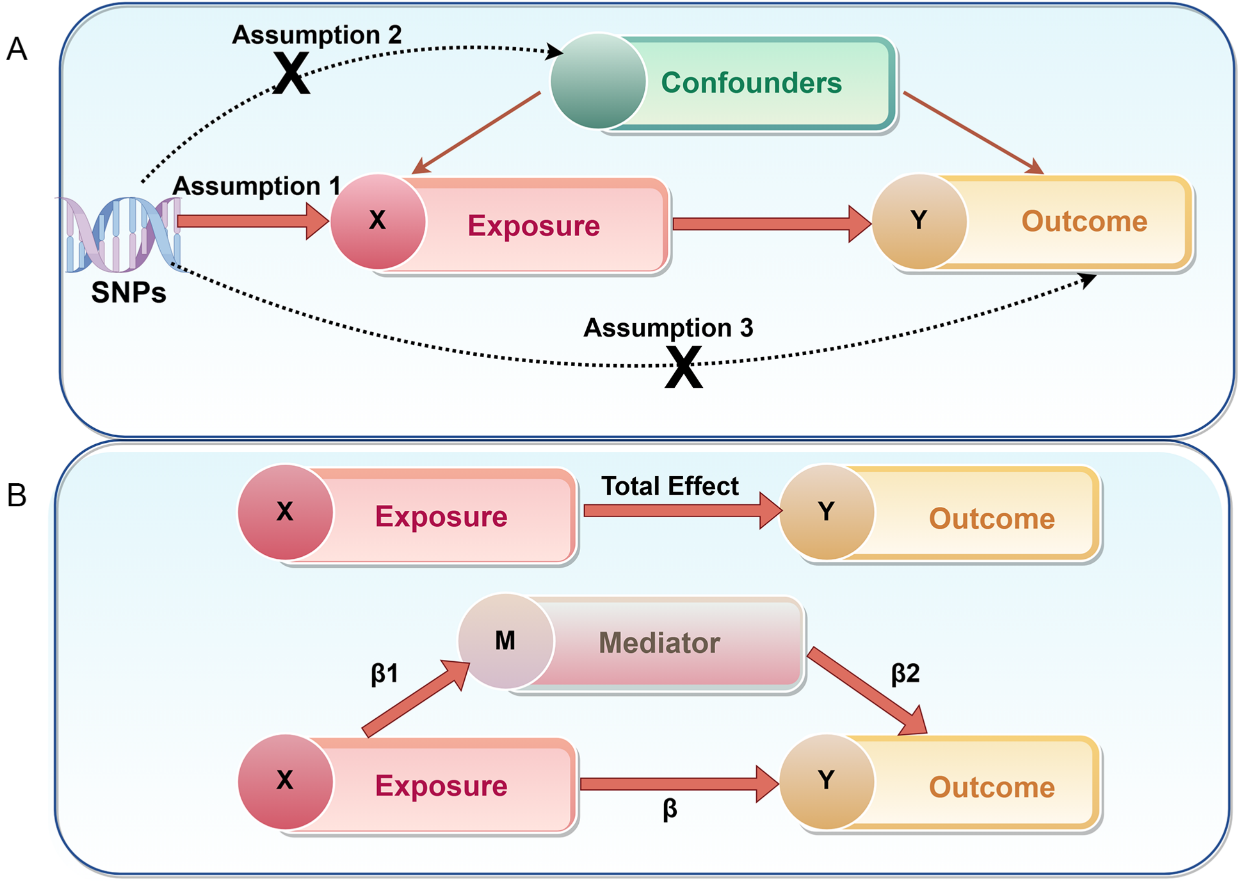

A
Assumption 2
Confounders
Assumption 1
X
Exposure
Y
Outcome
SNPs
Assumption 3
B
X
Exposure
Total Effect
Y
Outcome
M
Mediator
β1
β2
X
Exposure
β
Y
Outcome

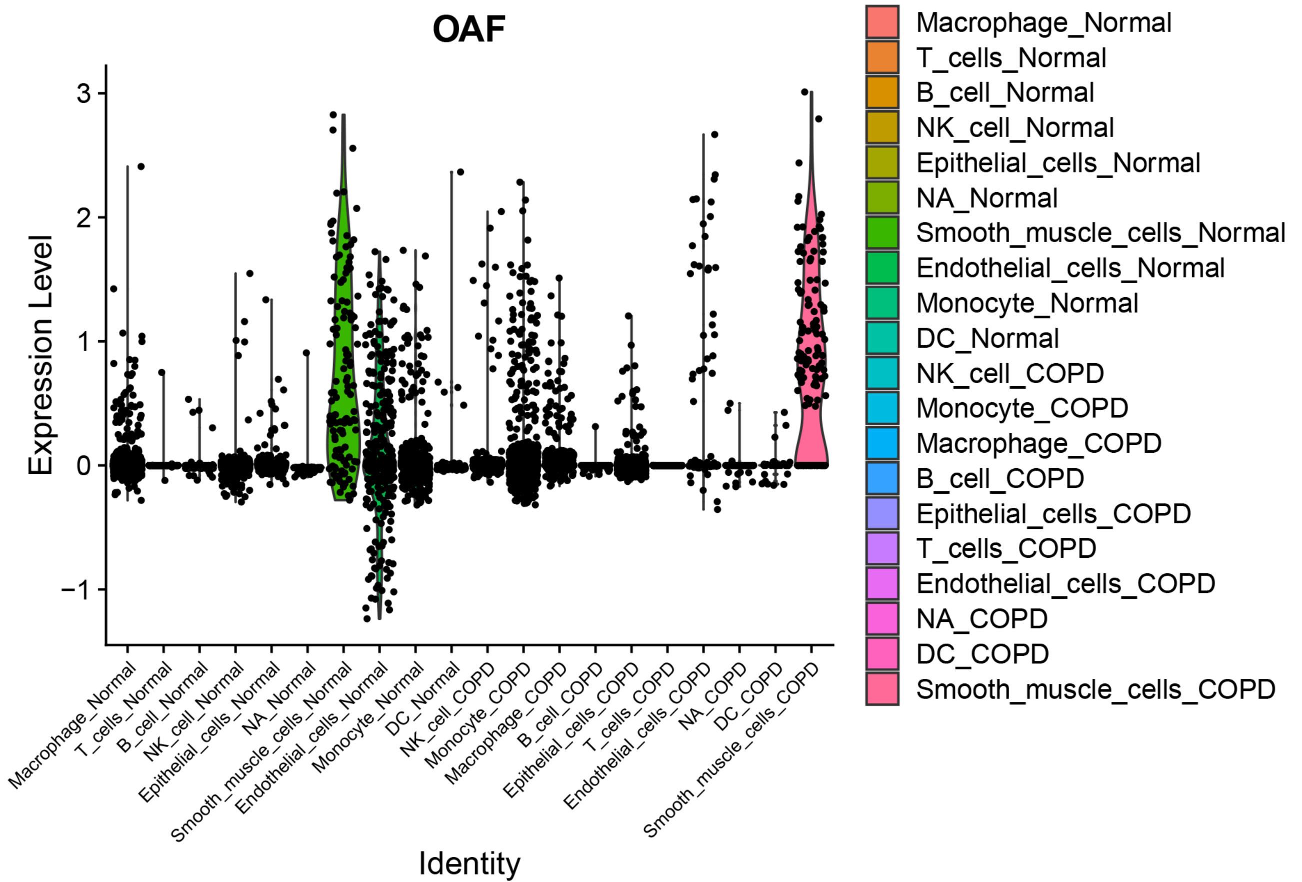

OAF
Expression Level
3
2
1
0
−1
Identity
Macrophage_Normal
T_cells_Normal
B_cell_Normal
NK_cell_Normal
Epithelial_cells_Normal
NA_Normal
Smooth_muscle_cells_Normal
Endothelial_cells_Normal
Monocyte_Normal
DC_Normal
NK_cell_COPD
Monocyte_COPD
Macrophage_COPD
B_cell_COPD
Epithelial_cells_COPD
T_cells_COPD
Endothelial_cells_COPD
NA_COPD
DC_COPD
Smooth_muscle_cells_COPD

Table S1 731 immune cell phenotypes
Table S2 Marker genes in NK cells of COPD tissue
Table S3  MR results of Immune cells and COPD using the IVW method
Table S4  Results of the Mendelian MR for OAF, Immune Cells, and CO
Table S5  Results of the Mendelian MR for OAF, Immune Cells, and CO

|
PD
PD

| Trait type | Panel | Statistical trai | Trait | GWAS Catalog Ac | id |
|---|---|---|---|---|---|
| Relative count | B cell | BC_Trait1 | IgD+ %B cell | GCST90001391 | ebi-a-GCS |
| Absolute count | B cell | BC_Trait10 | IgD+ CD38br A | GCST90001392 | ebi-a-GCS |
| Relative count | B cell | BC_Trait11 | IgD+ CD38dim ' | GCST90001393 | ebi-a-GCS |
| Absolute count | B cell | BC_Trait12 | IgD+ CD38dim | GCST90001394 | ebi-a-GCS |
| Relative count | B cell | BC_Trait13 | IgD+ CD38- %B | GCST90001395 | ebi-a-GCS |
| Absolute count | B cell | BC_Trait14 | IgD+ CD38- AC | GCST90001396 | ebi-a-GCS |
| Relative count | B cell | BC_Trait17 | Unsw mem %B | GCST90001397 | ebi-a-GCS |
| Absolute count | B cell | BC_Trait18 | Unsw mem AC | GCST90001398 | ebi-a-GCS |
| Relative count | B cell | BC_Trait19 | IgD- CD27- %B | GCST90001399 | ebi-a-GCS |
| Absolute count | B cell | BC_Trait2 | IgD+ AC | GCST90001400 | ebi-a-GCS |
| Absolute count | B cell | BC_Trait20 | IgD- CD27- AC | GCST90001401 | ebi-a-GCS |
| Relative count | B cell | BC_Trait21 | Sw mem %B ce | GCST90001402 | ebi-a-GCS |
| Absolute count | B cell | BC_Trait22 | Sw mem AC | GCST90001403 | ebi-a-GCS |
| Relative count | B cell | BC_Trait23 | PB/PC %B cell | GCST90001404 | ebi-a-GCS |
| Absolute count | B cell | BC_Trait24 | PB/PC AC | GCST90001405 | ebi-a-GCS |
| Relative count | B cell | BC_Trait25 | Memory B cell % | GCST90001406 | ebi-a-GCS |
| Absolute count | B cell | BC_Trait26 | Memory B cell | GCST90001407 | ebi-a-GCS |
| Relative count | B cell | BC_Trait27 | Naive-mature B | GCST90001408 | ebi-a-GCS |
| Absolute count | B cell | BC_Trait28 | Naive-mature B | GCST90001409 | ebi-a-GCS |
| Relative count | B cell | BC_Trait3 | IgD- CD38br %E | GCST90001410 | ebi-a-GCS |
| Relative count | B cell | BC_Trait31 | IgD+ CD24+ %E | GCST90001411 | ebi-a-GCS |
| Absolute count | B cell | BC_Trait32 | IgD+ CD24+ AC | GCST90001412 | ebi-a-GCS |
| Relative count | B cell | BC_Trait33 | IgD- CD24- %B | GCST90001413 | ebi-a-GCS |
| Absolute count | B cell | BC_Trait34 | IgD- CD24- AC | GCST90001414 | ebi-a-GCS |
| Relative count | B cell | BC_Trait35 | IgD+ CD24- %B | GCST90001415 | ebi-a-GCS |
| Absolute count | B cell | BC_Trait36 | IgD+ CD24- AC | GCST90001416 | ebi-a-GCS |
| Relative count | B cell | BC_Trait37 | CD24+ CD27+ ' | GCST90001417 | ebi-a-GCS |
| Absolute count | B cell | BC_Trait38 | CD24+ CD27+ | GCST90001418 | ebi-a-GCS |
| Relative count | B cell | BC_Trait39 | CD20- %B cell | GCST90001419 | ebi-a-GCS |
| Absolute count | B cell | BC_Trait4 | IgD- CD38br AC | GCST90001420 | ebi-a-GCS |
| Absolute count | B cell | BC_Trait40 | CD20- AC | GCST90001421 | ebi-a-GCS |
| Relative count | B cell | BC_Trait43 | CD20- CD38- % | GCST90001422 | ebi-a-GCS |
| Absolute count | B cell | BC_Trait44 | CD20- CD38- A | GCST90001423 | ebi-a-GCS |
| Relative count | B cell | BC_Trait46 | IgD+ %Lymphoc | GCST90001424 | ebi-a-GCS |
| Relative count | B cell | BC_Trait47 | IgD- CD38br %l | GCST90001425 | ebi-a-GCS |
| Relative count | B cell | BC_Trait48 | IgD- CD38dim % | GCST90001426 | ebi-a-GCS |
| Relative count | B cell | BC_Trait49 | IgD- CD38- %ly | GCST90001427 | ebi-a-GCS |
| Relative count | B cell | BC_Trait5 | IgD- CD38dim % | GCST90001428 | ebi-a-GCS |
| Relative count | B cell | BC_Trait50 | IgD+ CD38br % | GCST90001429 | ebi-a-GCS |
| Relative count | B cell | BC_Trait51 | IgD+ CD38dim ' | GCST90001430 | ebi-a-GCS |
| Relative count | B cell | BC_Trait52 | IgD+ CD38- %ly | GCST90001431 | ebi-a-GCS |
| Relative count | B cell | BC_Trait54 | Unsw Mem %lyı | GCST90001432 | ebi-a-GCS |
| Relative count | B cell | BC_Trait55 | IgD- CD27- %ly | GCST90001433 | ebi-a-GCS |
| Relative count | B cell | BC_Trait56 | Sw mem %lymp | GCST90001434 | ebi-a-GCS |

| | | | | | |
|---|---|---|---|---|---|
| Relative count | B cell | BC_Trait57 | PB/PC %lymph | GCST90001435 | ebi-a-GC |
| Relative count | B cell | BC_Trait58 | Memory B cell | GCST90001436 | ebi-a-GC |
| Relative count | B cell | BC_Trait59 | Naive-mature B | GCST90001437 | ebi-a-GC |
| Absolute count | B cell | BC_Trait6 | IgD- CD38dim A | GCST90001438 | ebi-a-GC |
| Relative count | B cell | BC_Trait61 | IgD+ CD24+ %l | GCST90001439 | ebi-a-GC |
| Relative count | B cell | BC_Trait62 | IgD- CD24- %ly | GCST90001440 | ebi-a-GC |
| Relative count | B cell | BC_Trait63 | IgD+ CD24- %ly | GCST90001441 | ebi-a-GC |
| Relative count | B cell | BC_Trait64 | CD24+ CD27+ | GCST90001442 | ebi-a-GC |
| Relative count | B cell | BC_Trait65 | CD20- %lymph | GCST90001443 | ebi-a-GC |
| Relative count | B cell | BC_Trait67 | CD20- CD38- % | GCST90001444 | ebi-a-GC |
| Relative count | B cell | BC_Trait7 | IgD- CD38- %B | GCST90001445 | ebi-a-GC |
| Absolute count | B cell | BC_Trait8 | IgD- CD38- AC | GCST90001446 | ebi-a-GC |
| Relative count | B cell | BC_Trait9 | IgD+ CD38br % | GCST90001447 | ebi-a-GC |
| Absolute count | cDC | DC_Trait524 | CD11c+ monoc | GCST90001448 | ebi-a-GC |
| Relative count | cDC | DC_Trait525 | CD11c+ monoc | GCST90001449 | ebi-a-GC |
| Absolute count | cDC | DC_Trait526 | CD62L- monocy | GCST90001450 | ebi-a-GC |
| Relative count | cDC | DC_Trait527 | CD62L- monocy | GCST90001451 | ebi-a-GC |
| Absolute count | cDC | DC_Trait530 | CD11c+ CD62L | GCST90001452 | ebi-a-GC |
| Relative count | cDC | DC_Trait531 | CD11c+ CD62L | GCST90001453 | ebi-a-GC |
| Absolute count | cDC | DC_Trait532 | CD62L- HLA DF | GCST90001454 | ebi-a-GC |
| Relative count | cDC | DC_Trait533 | CD62L- HLA DF | GCST90001455 | ebi-a-GC |
| Absolute count | cDC | DC_Trait534 | CD11c+ HLA DI | GCST90001456 | ebi-a-GC |
| Relative count | cDC | DC_Trait535 | CD11c+ HLA DI | GCST90001457 | ebi-a-GC |
| Absolute count | cDC | f_DC_Trait502 | Myeloid DC AC | GCST90001458 | ebi-a-GC |
| Relative count | cDC | f_DC_Trait503 | Myeloid DC %D | GCST90001459 | ebi-a-GC |
| Absolute count | cDC | f_DC_Trait504 | Plasmacytoid D | GCST90001460 | ebi-a-GC |
| Absolute count | cDC | f_DC_Trait505 | DC AC | GCST90001461 | ebi-a-GC |
| Absolute count | cDC | f_DC_Trait508 | CD62L- DC AC | GCST90001462 | ebi-a-GC |
| Relative count | cDC | f_DC_Trait509 | CD62L- DC %D | GCST90001463 | ebi-a-GC |
| Absolute count | cDC | f_DC_Trait512 | CD86+ myeloid | GCST90001464 | ebi-a-GC |
| Relative count | cDC | f_DC_Trait513 | CD86+ myeloid | GCST90001465 | ebi-a-GC |
| Absolute count | cDC | f_DC_Trait514 | CD86+ plasmac | GCST90001466 | ebi-a-GC |
| Relative count | cDC | f_DC_Trait515 | CD86+ plasmac | GCST90001467 | ebi-a-GC |
| Absolute count | cDC | f_DC_Trait516 | CD62L- myeloid | GCST90001468 | ebi-a-GC |
| Relative count | cDC | f_DC_Trait517 | CD62L- myeloi | GCST90001469 | ebi-a-GC |
| Absolute count | cDC | f_DC_Trait518 | CD62L- plasma | GCST90001470 | ebi-a-GC |
| Relative count | cDC | f_DC_Trait519 | CD62L- plasma | GCST90001471 | ebi-a-GC |
| Absolute count | cDC | f_DC_Trait520 | CD62L- CD86+ | GCST90001472 | ebi-a-GC |
| Relative count | cDC | f_DC_Trait521 | CD62L- CD86+ | GCST90001473 | ebi-a-GC |
| Relative count | cDC | f_DC_Trait522 | Plasmacytoid | GCST90001474 | ebi-a-GC |
| Relative count | TBNK | f_MONO_Trait | HLA DR++ mon | GCST90001475 | ebi-a-GC |
| Relative count | TBNK | f_MONO_Trait | HLA DR++ mon | GCST90001476 | ebi-a-GC |
| Absolute count | TBNK | f_MONO_Trait | HLA DR++ mon | GCST90001477 | ebi-a-GC |
| Relative count | Treg | f_Treg_Trait50 | CD4 Treg %CD | GCST90001478 | ebi-a-GC |
| Relative count | Treg | f_Treg_Trait50 | CD4 Treg %T c | GCST90001479 | ebi-a-GC |

| | | | | | |
|---|---|---|---|---|---|
| Absolute count | Treg | f_Treg_Trait50 | Resting Treg AC | GCST90001480 | ebi-a-GCS |
| Relative count | Treg | f_Treg_Trait50 | Resting Treg % | GCST90001481 | ebi-a-GCS |
| Relative count | Treg | f_Treg_Trait50 | Resting Treg % | GCST90001482 | ebi-a-GCS |
| Absolute count | Treg | f_Treg_Trait50 | CD39+ resting T | GCST90001483 | ebi-a-GCS |
| Relative count | Treg | f_Treg_Trait50 | CD39+ resting T | GCST90001484 | ebi-a-GCS |
| Relative count | Treg | f_Treg_Trait50 | CD39+ resting | GCST90001485 | ebi-a-GCS |
| Absolute count | Treg | f_Treg_Trait50 | Activated Treg | GCST90001486 | ebi-a-GCS |
| Relative count | Treg | f_Treg_Trait51 | Activated Treg | GCST90001487 | ebi-a-GCS |
| Relative count | Treg | f_Treg_Trait51 | Activated Treg | GCST90001488 | ebi-a-GCS |
| Absolute count | Treg | f_Treg_Trait51 | CD39+ activated | GCST90001489 | ebi-a-GCS |
| Relative count | Treg | f_Treg_Trait51 | CD39+ activated | GCST90001490 | ebi-a-GCS |
| Relative count | Treg | f_Treg_Trait51 | CD39+ activate | GCST90001491 | ebi-a-GCS |
| Absolute count | Treg | f_Treg_Trait51 | Secreting Treg | GCST90001492 | ebi-a-GCS |
| Relative count | Treg | f_Treg_Trait51 | Secreting Treg | GCST90001493 | ebi-a-GCS |
| Relative count | Treg | f_Treg_Trait51 | Secreting Treg | GCST90001494 | ebi-a-GCS |
| Absolute count | Treg | f_Treg_Trait51 | CD39+ secreting | GCST90001495 | ebi-a-GCS |
| Relative count | Treg | f_Treg_Trait51 | CD39+ secreting | GCST90001496 | ebi-a-GCS |
| Relative count | Treg | f_Treg_Trait52 | CD39+ secretin | GCST90001497 | ebi-a-GCS |
| Absolute count | Treg | f_Treg_Trait52 | Activated & rest | GCST90001498 | ebi-a-GCS |
| Relative count | Treg | f_Treg_Trait52 | Activated & res | GCST90001499 | ebi-a-GCS |
| Relative count | Treg | f_Treg_Trait52 | Activated & res | GCST90001500 | ebi-a-GCS |
| Absolute count | Treg | f_Treg_Trait52 | Activated & secr | GCST90001501 | ebi-a-GCS |
| Relative count | Treg | f_Treg_Trait52 | Activated & sec | GCST90001502 | ebi-a-GCS |
| Relative count | Treg | f_Treg_Trait52 | Activated & sec | GCST90001503 | ebi-a-GCS |
| Absolute count | Treg | f_Treg_Trait53 | CD25hi AC | GCST90001504 | ebi-a-GCS |
| Relative count | Treg | f_Treg_Trait53 | CD25hi %CD4+ | GCST90001505 | ebi-a-GCS |
| Relative count | Treg | f_Treg_Trait53 | CD25hi %T cell | GCST90001506 | ebi-a-GCS |
| Absolute count | Treg | f_Treg_Trait53 | CD25hi CD45RA | GCST90001507 | ebi-a-GCS |
| Relative count | Treg | f_Treg_Trait53 | CD25hi CD45RA | GCST90001508 | ebi-a-GCS |
| Relative count | Treg | f_Treg_Trait53 | CD25hi CD45RA | GCST90001509 | ebi-a-GCS |
| Absolute count | Treg | f_Treg_Trait53 | CD25hi CD45RA | GCST90001510 | ebi-a-GCS |
| Relative count | Treg | f_Treg_Trait54 | CD25hi CD45RA | GCST90001511 | ebi-a-GCS |
| Relative count | Treg | f_Treg_Trait54 | CD25hi CD45RA | GCST90001512 | ebi-a-GCS |
| Absolute count | Treg | f_Treg_Trait54 | CD4 Treg AC | GCST90001513 | ebi-a-GCS |
| Absolute count | Myeloid c | MDSC_Trait1 | HSC AC | GCST90001514 | ebi-a-GCS |
| Absolute count | Myeloid c | MDSC_Trait10 | Im MDSC AC | GCST90001515 | ebi-a-GCS |
| Relative count | Myeloid c | MDSC_Trait11 | Im MDSC %CD3 | GCST90001516 | ebi-a-GCS |
| Absolute count | Myeloid c | MDSC_Trait12 | CD33br HLA DR | GCST90001517 | ebi-a-GCS |
| Absolute count | Myeloid c | MDSC_Trait13 | CD33br HLA DR | GCST90001518 | ebi-a-GCS |
| Relative count | Myeloid c | MDSC_Trait14 | CD33br HLA DR | GCST90001519 | ebi-a-GCS |
| Absolute count | Myeloid c | MDSC_Trait15 | CD33br HLA DR | GCST90001520 | ebi-a-GCS |
| Relative count | Myeloid c | MDSC_Trait16 | CD33br HLA DR | GCST90001521 | ebi-a-GCS |
| Absolute count | Myeloid c | MDSC_Trait22 | CD33- HLA DR- | GCST90001522 | ebi-a-GCS |
| Absolute count | Myeloid c | MDSC_Trait23 | CD33- HLA DR- | GCST90001523 | ebi-a-GCS |
| Absolute count | Myeloid c | MDSC_Trait24 | Gr MDSC AC | GCST90001524 | ebi-a-GCS |

| | | | | | |
|---|---|---|---|---|---|
| Absolute count | Myeloid c | MDSC_Trait25 | CD33dim HLA D | GCST90001525 | ebi-a-GCS |
| Relative count | Myeloid c | MDSC_Trait26 | CD33dim HLA D | GCST90001526 | ebi-a-GCS |
| Absolute count | Myeloid c | MDSC_Trait27 | CD33dim HLA D | GCST90001527 | ebi-a-GCS |
| Relative count | Myeloid c | MDSC_Trait28 | CD33dim HLA D | GCST90001528 | ebi-a-GCS |
| Absolute count | Myeloid c | MDSC_Trait3 | CD66b++ myelo | GCST90001529 | ebi-a-GCS |
| Absolute count | Myeloid c | MDSC_Trait4 | Mo MDSC AC | GCST90001530 | ebi-a-GCS |
| Absolute count | Myeloid c | MDSC_Trait5 | CD33dim HLA D | GCST90001531 | ebi-a-GCS |
| Absolute count | Myeloid c | MDSC_Trait8 | Basophil AC | GCST90001532 | ebi-a-GCS |
| Relative count | Myeloid c | MDSC_Trait9 | Basophil %CD3 | GCST90001533 | ebi-a-GCS |
| Absolute count | Maturatio | MT_ry_Trait51 | CD45RA- CD4+ | GCST90001534 | ebi-a-GCS |
| Relative count | Maturatio | MT_ry_Trait51 | CD45RA- CD4+ | GCST90001535 | ebi-a-GCS |
| Relative count | Maturatio | MT_ry_Trait51 | CD45RA- CD4+ | GCST90001536 | ebi-a-GCS |
| Absolute count | Maturatio | MT_Trait503 | CM CD4+ AC | GCST90001537 | ebi-a-GCS |
| Relative count | Maturatio | MT_Trait504 | CM CD4+ %CD | GCST90001538 | ebi-a-GCS |
| Relative count | Maturatio | MT_Trait505 | CM CD4+ %T c | GCST90001539 | ebi-a-GCS |
| Absolute count | Maturatio | MT_Trait506 | Naive CD4+ AC | GCST90001540 | ebi-a-GCS |
| Relative count | Maturatio | MT_Trait507 | Naive CD4+ %C | GCST90001541 | ebi-a-GCS |
| Absolute count | Maturatio | MT_Trait508 | EM CD4+ AC | GCST90001542 | ebi-a-GCS |
| Relative count | Maturatio | MT_Trait509 | EM CD4+ %CD | GCST90001543 | ebi-a-GCS |
| Relative count | Maturatio | MT_Trait510 | EM CD4+ %T c | GCST90001544 | ebi-a-GCS |
| Absolute count | Maturatio | MT_Trait511 | TD CD4+ AC | GCST90001545 | ebi-a-GCS |
| Relative count | Maturatio | MT_Trait512 | TD CD4+ %CD4 | GCST90001546 | ebi-a-GCS |
| Relative count | Maturatio | MT_Trait513 | TD CD4+ %T ce | GCST90001547 | ebi-a-GCS |
| Absolute count | Maturatio | MT_Trait522 | CM CD8br AC | GCST90001548 | ebi-a-GCS |
| Relative count | Maturatio | MT_Trait523 | CM  CD8br %CI | GCST90001549 | ebi-a-GCS |
| Relative count | Maturatio | MT_Trait524 | CM CD8br %T c | GCST90001550 | ebi-a-GCS |
| Absolute count | Maturatio | MT_Trait525 | Naive CD8br AC | GCST90001551 | ebi-a-GCS |
| Relative count | Maturatio | MT_Trait526 | Naive  CD8br % | GCST90001552 | ebi-a-GCS |
| Relative count | Maturatio | MT_Trait527 | Naive CD8br %T | GCST90001553 | ebi-a-GCS |
| Absolute count | Maturatio | MT_Trait528 | EM CD8br AC | GCST90001554 | ebi-a-GCS |
| Relative count | Maturatio | MT_Trait529 | EM CD8br %CD | GCST90001555 | ebi-a-GCS |
| Relative count | Maturatio | MT_Trait530 | EM CD8br %T c | GCST90001556 | ebi-a-GCS |
| Absolute count | Maturatio | MT_Trait531 | TD CD8br AC | GCST90001557 | ebi-a-GCS |
| Relative count | Maturatio | MT_Trait532 | TD  CD8br %CD | GCST90001558 | ebi-a-GCS |
| Relative count | Maturatio | MT_Trait533 | TD CD8br %T c | GCST90001559 | ebi-a-GCS |
| Absolute count | Maturatio | MT_Trait537 | CD45RA+ CD8b | GCST90001560 | ebi-a-GCS |
| Relative count | Maturatio | MT_Trait538 | CD45RA+ CD8b | GCST90001561 | ebi-a-GCS |
| Relative count | Maturatio | MT_Trait539 | CD45RA+ CD8b | GCST90001562 | ebi-a-GCS |
| Absolute count | Maturatio | MT_Trait542 | CM DN (CD4-CI | GCST90001563 | ebi-a-GCS |
| Relative count | Maturatio | MT_Trait543 | CM DN (CD4-CI | GCST90001564 | ebi-a-GCS |
| Relative count | Maturatio | MT_Trait544 | CM DN (CD4-CI | GCST90001565 | ebi-a-GCS |
| Absolute count | Maturatio | MT_Trait545 | Naive DN (CD4- | GCST90001566 | ebi-a-GCS |
| Relative count | Maturatio | MT_Trait546 | Naive DN (CD4 | GCST90001567 | ebi-a-GCS |
| Relative count | Maturatio | MT_Trait547 | Naive DN (CD4- | GCST90001568 | ebi-a-GCS |
| Absolute count | Maturatio | MT_Trait548 | EM DN (CD4-CI | GCST90001569 | ebi-a-GCS |

| | | | | | |
|---|---|---|---|---|---|
| Relative count | Maturatio | MT_Trait549 | EM DN (CD4-C | GCST90001570 | ebi-a-GCS |
| Relative count | Maturatio | MT_Trait550 | EM DN (CD4-C | GCST90001571 | ebi-a-GCS |
| Absolute count | Maturatio | MT_Trait551 | TD DN (CD4-C | GCST90001572 | ebi-a-GCS |
| Relative count | Maturatio | MT_Trait552 | TD DN (CD4-C | GCST90001573 | ebi-a-GCS |
| Relative count | Maturatio | MT_Trait553 | TD DN (CD4-C | GCST90001574 | ebi-a-GCS |
| Relative count | Maturatio | MT_Trait560 | Naive CD4+ % | GCST90001575 | ebi-a-GCS |
| Relative count | B cell | N_BC_Trait3 | Transitional %B | GCST90001576 | ebi-a-GCS |
| Absolute count | B cell | N_BC_Trait4 | Transitional AC | GCST90001577 | ebi-a-GCS |
| Relative count | B cell | N_BC_Trait6 | Transitional %l | GCST90001578 | ebi-a-GCS |
| Absolute count | Monocyte | N_MONO_Trai | CD14- CD16+ n | GCST90001579 | ebi-a-GCS |
| Absolute count | Monocyte | N_MONO_Trai | CD14+ CD16+ r | GCST90001580 | ebi-a-GCS |
| Absolute count | Monocyte | N_MONO_Trai | CD14- CD16- A | GCST90001581 | ebi-a-GCS |
| Absolute count | Monocyte | N_MONO_Trai | CD14+ CD16- n | GCST90001582 | ebi-a-GCS |
| Absolute count | Monocyte | N_MONO_Trai | Monocyte AC | GCST90001583 | ebi-a-GCS |
| Relative count | Monocyte | N_MONO_Trai | CD14- CD16+ n | GCST90001584 | ebi-a-GCS |
| Relative count | Monocyte | N_MONO_Trai | CD14+ CD16+ r | GCST90001585 | ebi-a-GCS |
| Relative count | Monocyte | N_MONO_Trai | CD14+ CD16- n | GCST90001586 | ebi-a-GCS |
| Relative count | Monocyte | N_MONO_Trai | CD16+ monocy | GCST90001587 | ebi-a-GCS |
| Relative count | TBNK | RATIO_CD3_E | T/B | GCST90001588 | ebi-a-GCS |
| Relative count | TBNK | RATIO_CD4_C | CD4/CD8br | GCST90001589 | ebi-a-GCS |
| Absolute count | TBNK | TB_ry_Trait512 | CD4+ AC | GCST90001590 | ebi-a-GCS |
| Relative count | TBNK | TB_ry_Trait513 | CD4+ %T cell | GCST90001591 | ebi-a-GCS |
| Absolute count | TBNK | TB_ry_Trait515 | CD8br AC | GCST90001592 | ebi-a-GCS |
| Relative count | TBNK | TB_ry_Trait516 | CD8br %T cell | GCST90001593 | ebi-a-GCS |
| Absolute count | TBNK | TB_ry_Trait518 | DP (CD4+CD8+ | GCST90001594 | ebi-a-GCS |
| Relative count | TBNK | TB_ry_Trait519 | DP (CD4+CD8+ | GCST90001595 | ebi-a-GCS |
| Absolute count | TBNK | TB_ry_Trait524 | CD8dim AC | GCST90001596 | ebi-a-GCS |
| Relative count | TBNK | TB_ry_Trait525 | CD8dim %T cell | GCST90001597 | ebi-a-GCS |
| Absolute count | TBNK | TB_ry_Trait527 | DN (CD4-CD8-) | GCST90001598 | ebi-a-GCS |
| Relative count | TBNK | TB_ry_Trait528 | DN (CD4-CD8-) | GCST90001599 | ebi-a-GCS |
| Absolute count | TBNK | TB_Trait501 | Leukocyte AC | GCST90001600 | ebi-a-GCS |
| Absolute count | TBNK | TB_Trait507 | Lymphocyte AC | GCST90001601 | ebi-a-GCS |
| Relative count | TBNK | TB_Trait508 | Lymphocyte %le | GCST90001602 | ebi-a-GCS |
| Absolute count | TBNK | TB_Trait509 | T cell AC | GCST90001603 | ebi-a-GCS |
| Relative count | TBNK | TB_Trait510 | T cell %lymphoc | GCST90001604 | ebi-a-GCS |
| Relative count | TBNK | TB_Trait511 | T cell %leukocy | GCST90001605 | ebi-a-GCS |
| Relative count | TBNK | TB_Trait514 | CD4+ %leukocy | GCST90001606 | ebi-a-GCS |
| Relative count | TBNK | TB_Trait517 | CD8br %leukoc | GCST90001607 | ebi-a-GCS |
| Relative count | TBNK | TB_Trait520 | DP (CD4+CD8+ | GCST90001608 | ebi-a-GCS |
| Absolute count | TBNK | TB_Trait521 | CD4+ CD8dim | GCST90001609 | ebi-a-GCS |
| Relative count | TBNK | TB_Trait522 | CD4+ CD8dim % | GCST90001610 | ebi-a-GCS |
| Relative count | TBNK | TB_Trait523 | CD4+ CD8dim % | GCST90001611 | ebi-a-GCS |
| Relative count | TBNK | TB_Trait526 | CD8dim %leuko | GCST90001612 | ebi-a-GCS |
| Relative count | TBNK | TB_Trait529 | DN (CD4-CD8-) | GCST90001613 | ebi-a-GCS |
| Relative count | TBNK | TB_Trait532 | CD8br and CD8 | GCST90001614 | ebi-a-GCS |

| | | | | | |
|---|---|---|---|---|---|
| Absolute count | TBNK | TB_Trait533 | TCRgd AC | GCST90001615 | ebi-a-GC[illegible] |
| Relative count | TBNK | TB_Trait534 | TCRgd %T cell | GCST90001616 | ebi-a-GC[illegible] |
| Relative count | TBNK | TB_Trait535 | TCRgd %lymph[illegible] | GCST90001617 | ebi-a-GC[illegible] |
| Absolute count | TBNK | TB_Trait536 | HLA DR+ T cell | GCST90001618 | ebi-a-GC[illegible] |
| Relative count | TBNK | TB_Trait537 | HLA DR+ T cell[illegible] | GCST90001619 | ebi-a-GC[illegible] |
| Relative count | TBNK | TB_Trait538 | HLA DR+ T cell | GCST90001620 | ebi-a-GC[illegible] |
| Absolute count | TBNK | TB_Trait539 | NKT AC | GCST90001621 | ebi-a-GC[illegible] |
| Relative count | TBNK | TB_Trait540 | NKT %T cell | GCST90001622 | ebi-a-GC[illegible] |
| Relative count | TBNK | TB_Trait541 | NKT %lymphoc[illegible] | GCST90001623 | ebi-a-GC[illegible] |
| Absolute count | TBNK | TB_Trait542 | HLA DR+ CD4+ | GCST90001624 | ebi-a-GC[illegible] |
| Relative count | TBNK | TB_Trait543 | HLA DR+ CD4+ | GCST90001625 | ebi-a-GC[illegible] |
| Relative count | TBNK | TB_Trait544 | HLA DR+ CD4+ | GCST90001626 | ebi-a-GC[illegible] |
| Absolute count | TBNK | TB_Trait545 | HLA DR+ CD8b | GCST90001627 | ebi-a-GC[illegible] |
| Relative count | TBNK | TB_Trait546 | HLA DR+ CD8b | GCST90001628 | ebi-a-GC[illegible] |
| Relative count | TBNK | TB_Trait547 | HLA DR+ CD8b | GCST90001629 | ebi-a-GC[illegible] |
| Absolute count | TBNK | TB_Trait548 | CD8br NKT AC | GCST90001630 | ebi-a-GC[illegible] |
| Relative count | TBNK | TB_Trait549 | CD8br NKT %T | GCST90001631 | ebi-a-GC[illegible] |
| Relative count | TBNK | TB_Trait550 | CD8br NKT %ly | GCST90001632 | ebi-a-GC[illegible] |
| Absolute count | TBNK | TB_Trait551 | CD8dim NKT A | GCST90001633 | ebi-a-GC[illegible] |
| Relative count | TBNK | TB_Trait552 | CD8dim NKT % | GCST90001634 | ebi-a-GC[illegible] |
| Relative count | TBNK | TB_Trait553 | CD8dim NKT % | GCST90001635 | ebi-a-GC[illegible] |
| Absolute count | TBNK | TB_Trait554 | DN (CD4-CD8-) | GCST90001636 | ebi-a-GC[illegible] |
| Relative count | TBNK | TB_Trait555 | DN (CD4-CD8-) | GCST90001637 | ebi-a-GC[illegible] |
| Relative count | TBNK | TB_Trait556 | DN (CD4-CD8-) | GCST90001638 | ebi-a-GC[illegible] |
| Absolute count | TBNK | TB_Trait560 | CD3- lymphocyt | GCST90001639 | ebi-a-GC[illegible] |
| Relative count | TBNK | TB_Trait561 | CD3- lymphocyt | GCST90001640 | ebi-a-GC[illegible] |
| Relative count | TBNK | TB_Trait562 | CD3- lymphocyt | GCST90001641 | ebi-a-GC[illegible] |
| Absolute count | TBNK | TB_Trait563 | B cell AC | GCST90001642 | ebi-a-GC[illegible] |
| Relative count | TBNK | TB_Trait564 | B cell % CD3- l | GCST90001643 | ebi-a-GC[illegible] |
| Relative count | TBNK | TB_Trait565 | B cell %lympho[illegible] | GCST90001644 | ebi-a-GC[illegible] |
| Absolute count | TBNK | TB_Trait566 | NK AC | GCST90001645 | ebi-a-GC[illegible] |
| Relative count | TBNK | TB_Trait567 | NK %CD3- lym[illegible] | GCST90001646 | ebi-a-GC[illegible] |
| Relative count | TBNK | TB_Trait568 | NK %lymphocyt | GCST90001647 | ebi-a-GC[illegible] |
| Absolute count | TBNK | TB_Trait569 | HLA DR+ NK A | GCST90001648 | ebi-a-GC[illegible] |
| Relative count | TBNK | TB_Trait570 | HLA DR+ NK % | GCST90001649 | ebi-a-GC[illegible] |
| Relative count | TBNK | TB_Trait571 | HLA DR+ NK % | GCST90001650 | ebi-a-GC[illegible] |
| Absolute count | TBNK | TB_Trait575 | Granulocyte AC | GCST90001651 | ebi-a-GC[illegible] |
| Relative count | TBNK | TB_Trait576 | Granulocyte %l[illegible] | GCST90001652 | ebi-a-GC[illegible] |
| Relative count | Treg | Treg_Trait505 | CD28- DN (CD4 | GCST90001653 | ebi-a-GC[illegible] |
| Absolute count | Treg | Treg_Trait506 | CD28- DN (CD4 | GCST90001654 | ebi-a-GC[illegible] |
| Relative count | Treg | Treg_Trait507 | CD28+ DN (CD[illegible] | GCST90001655 | ebi-a-GC[illegible] |
| Relative count | Treg | Treg_Trait508 | CD28+ DN (CD[illegible] | GCST90001656 | ebi-a-GC[illegible] |
| Absolute count | Treg | Treg_Trait509 | CD28+ DN (CD[illegible] | GCST90001657 | ebi-a-GC[illegible] |
| Relative count | Treg | Treg_Trait512 | CD39+ CD4+ % | GCST90001658 | ebi-a-GC[illegible] |
| Relative count | Treg | Treg_Trait513 | CD39+ CD4+ % | GCST90001659 | ebi-a-GC[illegible] |

| | | | | | |
|---|---|---|---|---|---|
| Absolute count | Treg | Treg_Trait514 | CD39+ CD4+ A | GCST90001660 | ebi-a-GCS |
| Relative count | Treg | Treg_Trait574 | CD28- CD8dim | GCST90001661 | ebi-a-GCS |
| Relative count | Treg | Treg_Trait575 | CD28- CD8dim | GCST90001662 | ebi-a-GCS |
| Absolute count | Treg | Treg_Trait576 | CD28- CD8dim | GCST90001663 | ebi-a-GCS |
| Relative count | Treg | Treg_Trait577 | CD28+ CD45R | GCST90001664 | ebi-a-GCS |
| Relative count | Treg | Treg_Trait578 | CD28+ CD45R | GCST90001665 | ebi-a-GCS |
| Absolute count | Treg | Treg_Trait579 | CD28+ CD45R | GCST90001666 | ebi-a-GCS |
| Relative count | Treg | Treg_Trait580 | CD28+ CD45R | GCST90001667 | ebi-a-GCS |
| Relative count | Treg | Treg_Trait581 | CD28+ CD45R | GCST90001668 | ebi-a-GCS |
| Absolute count | Treg | Treg_Trait582 | CD28+ CD45R | GCST90001669 | ebi-a-GCS |
| Relative count | Treg | Treg_Trait585 | CD39+ CD8br % | GCST90001670 | ebi-a-GCS |
| Relative count | Treg | Treg_Trait586 | CD39+ CD8br % | GCST90001671 | ebi-a-GCS |
| Absolute count | Treg | Treg_Trait587 | CD39+ CD8br A | GCST90001672 | ebi-a-GCS |
| Relative count | Treg | Treg_Trait588 | CD28- CD127- ( | GCST90001673 | ebi-a-GCS |
| Relative count | Treg | Treg_Trait589 | CD28- CD127- | GCST90001674 | ebi-a-GCS |
| Absolute count | Treg | Treg_Trait590 | CD28- CD127- | GCST90001675 | ebi-a-GCS |
| Relative count | Treg | Treg_Trait591 | CD28- CD25++ | GCST90001676 | ebi-a-GCS |
| Relative count | Treg | Treg_Trait592 | CD28- CD25++ | GCST90001677 | ebi-a-GCS |
| Absolute count | Treg | Treg_Trait593 | CD28- CD25++ | GCST90001678 | ebi-a-GCS |
| Relative count | Treg | Treg_Trait594 | CD25++ CD8br | GCST90001679 | ebi-a-GCS |
| Relative count | Treg | Treg_Trait595 | CD25++ CD8br | GCST90001680 | ebi-a-GCS |
| Absolute count | Treg | Treg_Trait596 | CD25++ CD8br | GCST90001681 | ebi-a-GCS |
| Relative count | Treg | Treg_Trait597 | CD127- CD8br | GCST90001682 | ebi-a-GCS |
| Relative count | Treg | Treg_Trait598 | CD127- CD8br | GCST90001683 | ebi-a-GCS |
| Absolute count | Treg | Treg_Trait599 | CD127- CD8br | GCST90001684 | ebi-a-GCS |
| Relative count | Treg | Treg_Trait600 | CD28- CD8br % | GCST90001685 | ebi-a-GCS |
| Relative count | Treg | Treg_Trait601 | CD28- CD8br % | GCST90001686 | ebi-a-GCS |
| Absolute count | Treg | Treg_Trait602 | CD28- CD8br A | GCST90001687 | ebi-a-GCS |
| Relative count | Treg | Treg_Trait603 | CD28+ CD45R | GCST90001688 | ebi-a-GCS |
| Relative count | Treg | Treg_Trait604 | CD28+ CD45R | GCST90001689 | ebi-a-GCS |
| Absolute count | Treg | Treg_Trait605 | CD28+ CD45R | GCST90001690 | ebi-a-GCS |
| Relative count | Treg | Treg_Trait606 | CD28+ CD45R | GCST90001691 | ebi-a-GCS |
| Relative count | Treg | Treg_Trait607 | CD28+ CD45R | GCST90001692 | ebi-a-GCS |
| Absolute count | Treg | Treg_Trait608 | CD28+ CD45R | GCST90001693 | ebi-a-GCS |
| Relative count | Treg | Treg_Trait611 | CD28- DN (CD4 | GCST90001694 | ebi-a-GCS |
| Absolute count | Treg | Treg_Trait700 | CD45RA- CD28 | GCST90001695 | ebi-a-GCS |
| Relative count | Treg | Treg_Trait701 | CD45RA- CD28 | GCST90001696 | ebi-a-GCS |
| Relative count | Treg | Treg_Trait702 | CD45RA- CD28 | GCST90001697 | ebi-a-GCS |
| Absolute count | Treg | Treg_Trait706 | CD45RA+ CD2 | GCST90001698 | ebi-a-GCS |
| Relative count | Treg | Treg_Trait707 | CD45RA+ CD2 | GCST90001699 | ebi-a-GCS |
| Relative count | Treg | Treg_Trait708 | CD45RA+ CD2 | GCST90001700 | ebi-a-GCS |
| MFI | B cell | Bcells.trait111 | BAFF-R on CD | GCST90001701 | ebi-a-GCS |
| MFI | B cell | Bcells.trait112 | BAFF-R on CD | GCST90001702 | ebi-a-GCS |
| MFI | B cell | Bcells.trait137 | BAFF-R on IgD | GCST90001703 | ebi-a-GCS |
| MFI | B cell | Bcells.trait138 | BAFF-R on IgD- | GCST90001704 | ebi-a-GCS |

| | | | | | |
|---|---|---|---|---|---|
| MFI | B cell | Bcells.trait139 | BAFF-R on IgD· | GCST90001705 | ebi-a-GCS |
| MFI | B cell | Bcells.trait140 | BAFF-R on IgD· | GCST90001706 | ebi-a-GCS |
| MFI | B cell | Bcells.trait141 | BAFF-R on IgD | GCST90001707 | ebi-a-GCS |
| MFI | B cell | Bcells.trait142 | BAFF-R on IgD· | GCST90001708 | ebi-a-GCS |
| MFI | B cell | Bcells.trait143 | BAFF-R on IgD | GCST90001709 | ebi-a-GCS |
| MFI | B cell | Bcells.trait147 | BAFF-R on IgD- | GCST90001710 | ebi-a-GCS |
| MFI | B cell | Bcells.trait148 | BAFF-R on IgD- | GCST90001711 | ebi-a-GCS |
| MFI | B cell | Bcells.trait149 | BAFF-R on IgD- | GCST90001712 | ebi-a-GCS |
| MFI | B cell | Bcells.trait150 | BAFF-R on IgD- | GCST90001713 | ebi-a-GCS |
| MFI | B cell | Bcells.trait151 | BAFF-R on IgD- | GCST90001714 | ebi-a-GCS |
| MFI | B cell | Bcells.trait154 | BAFF-R on mem | GCST90001715 | ebi-a-GCS |
| MFI | B cell | Bcells.trait156 | BAFF-R on naiv | GCST90001716 | ebi-a-GCS |
| MFI | B cell | Bcells.trait157 | BAFF-R on uns | GCST90001717 | ebi-a-GCS |
| MFI | B cell | Bcells.trait159 | BAFF-R on sw | GCST90001718 | ebi-a-GCS |
| MFI | B cell | Bcells.trait160 | BAFF-R on IgD· | GCST90001719 | ebi-a-GCS |
| MFI | B cell | Bcells.trait161 | BAFF-R on tran | GCST90001720 | ebi-a-GCS |
| MFI | B cell | Bcells.trait255 | CD19 on CD20- | GCST90001721 | ebi-a-GCS |
| MFI | B cell | Bcells.trait282 | CD19 on CD20- | GCST90001722 | ebi-a-GCS |
| MFI | B cell | Bcells.trait283 | CD19 on CD24+ | GCST90001723 | ebi-a-GCS |
| MFI | B cell | Bcells.trait308 | CD19 on IgD+ | GCST90001724 | ebi-a-GCS |
| MFI | B cell | Bcells.trait309 | CD19 on IgD+ C | GCST90001725 | ebi-a-GCS |
| MFI | B cell | Bcells.trait310 | CD19 on IgD+ C | GCST90001726 | ebi-a-GCS |
| MFI | B cell | Bcells.trait311 | CD19 on IgD+ C | GCST90001727 | ebi-a-GCS |
| MFI | B cell | Bcells.trait312 | CD19 on IgD+ | GCST90001728 | ebi-a-GCS |
| MFI | B cell | Bcells.trait313 | CD19 on IgD+ C | GCST90001729 | ebi-a-GCS |
| MFI | B cell | Bcells.trait314 | CD19 on IgD+ | GCST90001730 | ebi-a-GCS |
| MFI | B cell | Bcells.trait318 | CD19 on IgD- C | GCST90001731 | ebi-a-GCS |
| MFI | B cell | Bcells.trait319 | CD19 on IgD- C | GCST90001732 | ebi-a-GCS |
| MFI | B cell | Bcells.trait320 | CD19 on IgD- C | GCST90001733 | ebi-a-GCS |
| MFI | B cell | Bcells.trait321 | CD19 on IgD- C | GCST90001734 | ebi-a-GCS |
| MFI | B cell | Bcells.trait322 | CD19 on IgD- C | GCST90001735 | ebi-a-GCS |
| MFI | B cell | Bcells.trait325 | CD19 on memo | GCST90001736 | ebi-a-GCS |
| MFI | B cell | Bcells.trait327 | CD19 on naive-I | GCST90001737 | ebi-a-GCS |
| MFI | B cell | Bcells.trait328 | CD19 on unsw | GCST90001738 | ebi-a-GCS |
| MFI | B cell | Bcells.trait329 | CD19 on PB/PC | GCST90001739 | ebi-a-GCS |
| MFI | B cell | Bcells.trait331 | CD19 on sw me | GCST90001740 | ebi-a-GCS |
| MFI | B cell | Bcells.trait332 | CD19 on IgD+ | GCST90001741 | ebi-a-GCS |
| MFI | B cell | Bcells.trait333 | CD19 on transiti | GCST90001742 | ebi-a-GCS |
| MFI | B cell | Bcells.trait335 | CD20 on B cell | GCST90001743 | ebi-a-GCS |
| MFI | B cell | Bcells.trait354 | CD20 on CD20- | GCST90001744 | ebi-a-GCS |
| MFI | B cell | Bcells.trait355 | CD20 on CD24+ | GCST90001745 | ebi-a-GCS |
| MFI | B cell | Bcells.trait378 | CD20 on IgD+ | GCST90001746 | ebi-a-GCS |
| MFI | B cell | Bcells.trait379 | CD20 on IgD+ C | GCST90001747 | ebi-a-GCS |
| MFI | B cell | Bcells.trait380 | CD20 on IgD+ C | GCST90001748 | ebi-a-GCS |
| MFI | B cell | Bcells.trait381 | CD20 on IgD+ C | GCST90001749 | ebi-a-GCS |

| | | | | | |
|---|---|---|---|---|---|
| MFI | B cell | Bcells.trait382 | CD20 on IgD+ | GCST90001750 | ebi-a-GC[illegible] |
| MFI | B cell | Bcells.trait383 | CD20 on IgD+ ( | GCST90001751 | ebi-a-GC[illegible] |
| MFI | B cell | Bcells.trait384 | CD20 on IgD+ | GCST90001752 | ebi-a-GC[illegible] |
| MFI | B cell | Bcells.trait388 | CD20 on IgD- C | GCST90001753 | ebi-a-GC[illegible] |
| MFI | B cell | Bcells.trait389 | CD20 on IgD- C | GCST90001754 | ebi-a-GC[illegible] |
| MFI | B cell | Bcells.trait390 | CD20 on IgD- C | GCST90001755 | ebi-a-GC[illegible] |
| MFI | B cell | Bcells.trait391 | CD20 on IgD- C | GCST90001756 | ebi-a-GC[illegible] |
| MFI | B cell | Bcells.trait392 | CD20 on IgD- C | GCST90001757 | ebi-a-GC[illegible] |
| MFI | B cell | Bcells.trait395 | CD20 on memo | GCST90001758 | ebi-a-GC[illegible] |
| MFI | B cell | Bcells.trait397 | CD20 on naive-[illegible] | GCST90001759 | ebi-a-GC[illegible] |
| MFI | B cell | Bcells.trait398 | CD20 on unsw | GCST90001760 | ebi-a-GC[illegible] |
| MFI | B cell | Bcells.trait399 | CD20 on sw me | GCST90001761 | ebi-a-GC[illegible] |
| MFI | B cell | Bcells.trait400 | CD20 on IgD+ | GCST90001762 | ebi-a-GC[illegible] |
| MFI | B cell | Bcells.trait401 | CD20 on transiti | GCST90001763 | ebi-a-GC[illegible] |
| MFI | B cell | Bcells.trait418 | CD24 on CD24[illegible] | GCST90001764 | ebi-a-GC[illegible] |
| MFI | B cell | Bcells.trait438 | CD24 on IgD+ | GCST90001765 | ebi-a-GC[illegible] |
| MFI | B cell | Bcells.trait439 | CD24 on IgD+ ( | GCST90001766 | ebi-a-GC[illegible] |
| MFI | B cell | Bcells.trait441 | CD24 on IgD+ | GCST90001767 | ebi-a-GC[illegible] |
| MFI | B cell | Bcells.trait442 | CD24 on IgD+ ( | GCST90001768 | ebi-a-GC[illegible] |
| MFI | B cell | Bcells.trait445 | CD24 on IgD- C | GCST90001769 | ebi-a-GC[illegible] |
| MFI | B cell | Bcells.trait446 | CD24 on IgD- C | GCST90001770 | ebi-a-GC[illegible] |
| MFI | B cell | Bcells.trait448 | CD24 on memo | GCST90001771 | ebi-a-GC[illegible] |
| MFI | B cell | Bcells.trait449 | CD24 on unsw | GCST90001772 | ebi-a-GC[illegible] |
| MFI | B cell | Bcells.trait450 | CD24 on sw me | GCST90001773 | ebi-a-GC[illegible] |
| MFI | B cell | Bcells.trait451 | CD24 on transiti | GCST90001774 | ebi-a-GC[illegible] |
| MFI | B cell | Bcells.trait453 | CD25 on B cell | GCST90001775 | ebi-a-GC[illegible] |
| MFI | B cell | Bcells.trait470 | CD25 on CD20- | GCST90001776 | ebi-a-GC[illegible] |
| MFI | B cell | Bcells.trait471 | CD25 on CD24[illegible] | GCST90001777 | ebi-a-GC[illegible] |
| MFI | B cell | Bcells.trait490 | CD25 on IgD+ | GCST90001778 | ebi-a-GC[illegible] |
| MFI | B cell | Bcells.trait491 | CD25 on IgD+ ( | GCST90001779 | ebi-a-GC[illegible] |
| MFI | B cell | Bcells.trait492 | CD25 on IgD+ ( | GCST90001780 | ebi-a-GC[illegible] |
| MFI | B cell | Bcells.trait493 | CD25 on IgD+ ( | GCST90001781 | ebi-a-GC[illegible] |
| MFI | B cell | Bcells.trait494 | CD25 on IgD+ | GCST90001782 | ebi-a-GC[illegible] |
| MFI | B cell | Bcells.trait495 | CD25 on IgD+ ( | GCST90001783 | ebi-a-GC[illegible] |
| MFI | B cell | Bcells.trait496 | CD25 on IgD+ | GCST90001784 | ebi-a-GC[illegible] |
| MFI | B cell | Bcells.trait500 | CD25 on IgD- C | GCST90001785 | ebi-a-GC[illegible] |
| MFI | B cell | Bcells.trait501 | CD25 on IgD- C | GCST90001786 | ebi-a-GC[illegible] |
| MFI | B cell | Bcells.trait502 | CD25 on IgD- C | GCST90001787 | ebi-a-GC[illegible] |
| MFI | B cell | Bcells.trait503 | CD25 on IgD- C | GCST90001788 | ebi-a-GC[illegible] |
| MFI | B cell | Bcells.trait504 | CD25 on IgD- C | GCST90001789 | ebi-a-GC[illegible] |
| MFI | B cell | Bcells.trait506 | CD25 on memo | GCST90001790 | ebi-a-GC[illegible] |
| MFI | B cell | Bcells.trait508 | CD25 on naive-[illegible] | GCST90001791 | ebi-a-GC[illegible] |
| MFI | B cell | Bcells.trait509 | CD25 on unsw | GCST90001792 | ebi-a-GC[illegible] |
| MFI | B cell | Bcells.trait510 | CD25 on sw me | GCST90001793 | ebi-a-GC[illegible] |
| MFI | B cell | Bcells.trait511 | CD25 on IgD+ | GCST90001794 | ebi-a-GC[illegible] |

| | | | | | |
|---|---|---|---|---|---|
| MFI | B cell | Bcells.trait512 | CD25 on transiti | GCST90001795 | ebi-a-GC[illegible] |
| MFI | B cell | Bcells.trait514 | CD27 on CD20- | GCST90001796 | ebi-a-GC[illegible] |
| MFI | B cell | Bcells.trait536 | CD27 on CD20- | GCST90001797 | ebi-a-GC[illegible] |
| MFI | B cell | Bcells.trait537 | CD27 on CD24+ | GCST90001798 | ebi-a-GC[illegible] |
| MFI | B cell | Bcells.trait539 | CD27 on T cell | GCST90001799 | ebi-a-GC[illegible] |
| MFI | B cell | Bcells.trait561 | CD27 on IgD+ | GCST90001800 | ebi-a-GC[illegible] |
| MFI | B cell | Bcells.trait563 | CD27 on IgD+ | GCST90001801 | ebi-a-GC[illegible] |
| MFI | B cell | Bcells.trait565 | CD27 on IgD- C | GCST90001802 | ebi-a-GC[illegible] |
| MFI | B cell | Bcells.trait566 | CD27 on IgD- C | GCST90001803 | ebi-a-GC[illegible] |
| MFI | B cell | Bcells.trait567 | CD27 on IgD- C | GCST90001804 | ebi-a-GC[illegible] |
| MFI | B cell | Bcells.trait570 | CD27 on memo | GCST90001805 | ebi-a-GC[illegible] |
| MFI | B cell | Bcells.trait571 | CD27 on unsw | GCST90001806 | ebi-a-GC[illegible] |
| MFI | B cell | Bcells.trait572 | CD27 on PB/PC | GCST90001807 | ebi-a-GC[illegible] |
| MFI | B cell | Bcells.trait574 | CD27 on sw me | GCST90001808 | ebi-a-GC[illegible] |
| MFI | B cell | Bcells.trait597 | CD38 on CD20- | GCST90001809 | ebi-a-GC[illegible] |
| MFI | B cell | Bcells.trait619 | CD38 on CD3- | GCST90001810 | ebi-a-GC[illegible] |
| MFI | B cell | Bcells.trait637 | CD38 on IgD+ ( | GCST90001811 | ebi-a-GC[illegible] |
| MFI | B cell | Bcells.trait640 | CD38 on IgD+ ( | GCST90001812 | ebi-a-GC[illegible] |
| MFI | B cell | Bcells.trait641 | CD38 on IgD+ | GCST90001813 | ebi-a-GC[illegible] |
| MFI | B cell | Bcells.trait643 | CD38 on IgD- C | GCST90001814 | ebi-a-GC[illegible] |
| MFI | B cell | Bcells.trait644 | CD38 on IgD- C | GCST90001815 | ebi-a-GC[illegible] |
| MFI | B cell | Bcells.trait646 | CD38 on naive- | GCST90001816 | ebi-a-GC[illegible] |
| MFI | B cell | Bcells.trait647 | CD38 on PB/PC | GCST90001817 | ebi-a-GC[illegible] |
| MFI | B cell | Bcells.trait649 | CD38 on IgD+ | GCST90001818 | ebi-a-GC[illegible] |
| MFI | B cell | Bcells.trait650 | CD38 on transiti | GCST90001819 | ebi-a-GC[illegible] |
| MFI | B cell | Bcells.trait775 | IgD on IgD+ CD | GCST90001820 | ebi-a-GC[illegible] |
| MFI | B cell | Bcells.trait776 | IgD on IgD+ CD | GCST90001821 | ebi-a-GC[illegible] |
| MFI | B cell | Bcells.trait777 | IgD on IgD+ CD | GCST90001822 | ebi-a-GC[illegible] |
| MFI | B cell | Bcells.trait779 | IgD on IgD+ CD | GCST90001823 | ebi-a-GC[illegible] |
| MFI | B cell | Bcells.trait780 | IgD on IgD+ CD | GCST90001824 | ebi-a-GC[illegible] |
| MFI | B cell | Bcells.trait781 | IgD on IgD+ CD | GCST90001825 | ebi-a-GC[illegible] |
| MFI | B cell | Bcells.trait785 | IgD on unsw m | GCST90001826 | ebi-a-GC[illegible] |
| MFI | B cell | Bcells.trait786 | IgD on IgD+ | GCST90001827 | ebi-a-GC[illegible] |
| MFI | B cell | Bcells.trait787 | IgD on transitior | GCST90001828 | ebi-a-GC[illegible] |
| MFI | B cell | Bcells.trait84 | BAFF-R on B ce | GCST90001829 | ebi-a-GC[illegible] |
| MFI | B cell | Bcells.trait85 | BAFF-R on CD2 | GCST90001830 | ebi-a-GC[illegible] |
| MFI | cDC | Blue.530.30.cD | CD62L on CD62 | GCST90001831 | ebi-a-GC[illegible] |
| MFI | cDC | Blue.530.30.cD | CD62L on CD62 | GCST90001832 | ebi-a-GC[illegible] |
| MFI | cDC | Blue.530.30.cD | CD62L on CD62 | GCST90001833 | ebi-a-GC[illegible] |
| MFI | cDC | Blue.530.30.cD | CD62L on mono | GCST90001834 | ebi-a-GC[illegible] |
| MFI | cDC | Blue.530.30.cD | CD62L on granu | GCST90001835 | ebi-a-GC[illegible] |
| MFI | Myeloid c | Blue.530.30.MI | CD66b on CD66 | GCST90001836 | ebi-a-GC[illegible] |
| MFI | Myeloid c | Blue.530.30.MI | CD66b on Gr M | GCST90001837 | ebi-a-GC[illegible] |
| MFI | Maturatio | Blue.530.30.M | CD3 on naive C | GCST90001838 | ebi-a-GC[illegible] |
| MFI | Maturatio | Blue.530.30.M | CD3 on EM CD | GCST90001839 | ebi-a-GC[illegible] |

| | | | | | |
|---|---|---|---|---|---|
| MFI | Maturatio | Blue.530.30.M | CD3 on TD CD8 | GCST90001840 | ebi-a-GCS |
| MFI | Maturatio | Blue.530.30.M | CD3 on CM CD4 | GCST90001841 | ebi-a-GCS |
| MFI | Maturatio | Blue.530.30.M | CD3 on Naive C | GCST90001842 | ebi-a-GCS |
| MFI | Maturatio | Blue.530.30.M | CD3 on EM CD4 | GCST90001843 | ebi-a-GCS |
| MFI | Maturatio | Blue.530.30.M | CD3 on TD CD4 | GCST90001844 | ebi-a-GCS |
| MFI | Maturatio | Blue.530.30.M | CD3 on CD45RA | GCST90001845 | ebi-a-GCS |
| MFI | Maturatio | Blue.530.30.M | CD3 on CM CD8 | GCST90001846 | ebi-a-GCS |
| MFI | TBNK | Blue.530.30.TB | CD3 on HLA DR | GCST90001847 | ebi-a-GCS |
| MFI | TBNK | Blue.530.30.TB | CD3 on NKT | GCST90001848 | ebi-a-GCS |
| MFI | TBNK | Blue.530.30.TB | CD3 on HLA DR | GCST90001849 | ebi-a-GCS |
| MFI | TBNK | Blue.530.30.TB | CD3 on HLA DR | GCST90001850 | ebi-a-GCS |
| MFI | Treg | Blue.530.30.Tr | CD3 on T cell | GCST90001851 | ebi-a-GCS |
| MFI | Treg | Blue.530.30.Tr | CD3 on CD39+ | GCST90001852 | ebi-a-GCS |
| MFI | Treg | Blue.530.30.Tr | CD3 on activate | GCST90001853 | ebi-a-GCS |
| MFI | Treg | Blue.530.30.Tr | CD3 on CD39+ | GCST90001854 | ebi-a-GCS |
| MFI | Treg | Blue.530.30.Tr | CD3 on secretin | GCST90001855 | ebi-a-GCS |
| MFI | Treg | Blue.530.30.Tr | CD3 on CD39+ | GCST90001856 | ebi-a-GCS |
| MFI | Treg | Blue.530.30.Tr | CD3 on activate | GCST90001857 | ebi-a-GCS |
| MFI | Treg | Blue.530.30.Tr | CD3 on CD45R | GCST90001858 | ebi-a-GCS |
| MFI | Treg | Blue.530.30.Tr | CD3 on CD8br | GCST90001859 | ebi-a-GCS |
| MFI | Treg | Blue.530.30.Tr | CD3 on CD39+ | GCST90001860 | ebi-a-GCS |
| MFI | Treg | Blue.530.30.Tr | CD3 on CD28+ | GCST90001861 | ebi-a-GCS |
| MFI | Treg | Blue.530.30.Tr | CD3 on CD28+ | GCST90001862 | ebi-a-GCS |
| MFI | Treg | Blue.530.30.Tr | CD3 on CD28+ | GCST90001863 | ebi-a-GCS |
| MFI | Treg | Blue.530.30.Tr | CD3 on CD28+ | GCST90001864 | ebi-a-GCS |
| MFI | Treg | Blue.530.30.Tr | CD3 on CD28- ( | GCST90001865 | ebi-a-GCS |
| MFI | Treg | Blue.530.30.Tr | CD3 on CD39+ | GCST90001866 | ebi-a-GCS |
| MFI | Treg | Blue.530.30.Tr | CD3 on CD4+ | GCST90001867 | ebi-a-GCS |
| MFI | Treg | Blue.530.30.Tr | CD3 on CD4 Tr | GCST90001868 | ebi-a-GCS |
| MFI | Treg | Blue.530.30.Tr | CD3 on resting | GCST90001869 | ebi-a-GCS |
| MFI | Myeloid c | Blue.585.42.MI | CD34 on HSC | GCST90001870 | ebi-a-GCS |
| MFI | Maturatio | HVEM1000_15 | HVEM on T cell | GCST90001871 | ebi-a-GCS |
| MFI | Maturatio | HVEM1000_15 | HVEM on naive | GCST90001872 | ebi-a-GCS |
| MFI | Maturatio | HVEM1000_15 | HVEM on EM C | GCST90001873 | ebi-a-GCS |
| MFI | Maturatio | HVEM1000_15 | HVEM on TD C | GCST90001874 | ebi-a-GCS |
| MFI | Maturatio | HVEM1000_15 | HVEM on CD4+ | GCST90001875 | ebi-a-GCS |
| MFI | Maturatio | HVEM1000_15 | HVEM on CM C | GCST90001876 | ebi-a-GCS |
| MFI | Maturatio | HVEM1000_15 | HVEM on naive | GCST90001877 | ebi-a-GCS |
| MFI | Maturatio | HVEM1000_15 | HVEM on EM C | GCST90001878 | ebi-a-GCS |
| MFI | Maturatio | HVEM1000_15 | HVEM on TD C | GCST90001879 | ebi-a-GCS |
| MFI | Maturatio | HVEM1000_15 | HVEM on CD45 | GCST90001880 | ebi-a-GCS |
| MFI | Maturatio | HVEM1000_15 | HVEM on CD8b | GCST90001881 | ebi-a-GCS |
| MFI | Maturatio | HVEM1000_15 | HVEM on CM C | GCST90001882 | ebi-a-GCS |
| MFI | TBNK | Blue.585.42.TB | CD16-CD56 on | GCST90001883 | ebi-a-GCS |
| MFI | TBNK | Blue.585.42.TB | CD16-CD56 on | GCST90001884 | ebi-a-GCS |

| | | | | | |
|---|---|---|---|---|---|
| MFI | TBNK | Blue.585.42.TE | CD16-CD56 on | GCST90001885 | ebi-a-GCS |
| MFI | Treg | Blue.585.42.Tr | CD28 on CD39+ | GCST90001886 | ebi-a-GCS |
| MFI | Treg | Blue.585.42.Tr | CD28 on secreti | GCST90001887 | ebi-a-GCS |
| MFI | Treg | Blue.585.42.Tr | CD28 on CD39+ | GCST90001888 | ebi-a-GCS |
| MFI | Treg | Blue.585.42.Tr | CD28 on activat | GCST90001889 | ebi-a-GCS |
| MFI | Treg | Blue.585.42.Tr | CD28 on CD45F | GCST90001890 | ebi-a-GCS |
| MFI | Treg | Blue.585.42.Tr | CD28 on CD45F | GCST90001891 | ebi-a-GCS |
| MFI | Treg | Blue.585.42.Tr | CD28 on CD39+ | GCST90001892 | ebi-a-GCS |
| MFI | Treg | Blue.585.42.Tr | CD28 on CD28+ | GCST90001893 | ebi-a-GCS |
| MFI | Treg | Blue.585.42.Tr | CD28 on CD28+ | GCST90001894 | ebi-a-GCS |
| MFI | Treg | Blue.585.42.Tr | CD28 on CD28+ | GCST90001895 | ebi-a-GCS |
| MFI | Treg | Blue.585.42.Tr | CD28 on CD28+ | GCST90001896 | ebi-a-GCS |
| MFI | Treg | Blue.585.42.Tr | CD28 on CD39+ | GCST90001897 | ebi-a-GCS |
| MFI | Treg | Blue.585.42.Tr | CD28 on CD4+ | GCST90001898 | ebi-a-GCS |
| MFI | Treg | Blue.585.42.Tr | CD28 on CD4 T | GCST90001899 | ebi-a-GCS |
| MFI | Treg | Blue.585.42.Tr | CD28 on resting | GCST90001900 | ebi-a-GCS |
| MFI | Treg | Blue.585.42.Tr | CD28 on CD39+ | GCST90001901 | ebi-a-GCS |
| MFI | Treg | Blue.585.42.Tr | CD28 on activat | GCST90001902 | ebi-a-GCS |
| MFI | cDC | Blue.670.LP.cD | CD86 on myeloi | GCST90001903 | ebi-a-GCS |
| MFI | cDC | Blue.670.LP.cD | CD86 on CD62L | GCST90001904 | ebi-a-GCS |
| MFI | cDC | Blue.670.LP.cD | CD86 on monoc | GCST90001905 | ebi-a-GCS |
| MFI | cDC | Blue.670.LP.cD | CD86 on granul | GCST90001906 | ebi-a-GCS |
| MFI | Maturation | Blue.670.LP.M | CCR7 on naive | GCST90001907 | ebi-a-GCS |
| MFI | Maturation | Blue.670.LP.M | CCR7 on naive | GCST90001908 | ebi-a-GCS |
| MFI | TBNK | Blue.670.LP.TE | CD45 on CD14+ | GCST90001909 | ebi-a-GCS |
| MFI | TBNK | Blue.670.LP.TE | CD45 on B cell | GCST90001910 | ebi-a-GCS |
| MFI | TBNK | Blue.670.LP.TE | CD45 on NK | GCST90001911 | ebi-a-GCS |
| MFI | TBNK | Blue.670.LP.TE | CD45 on HLA D | GCST90001912 | ebi-a-GCS |
| MFI | TBNK | Blue.670.LP.TE | CD45 on granul | GCST90001913 | ebi-a-GCS |
| MFI | TBNK | Blue.670.LP.TE | CD45 on lympho | GCST90001914 | ebi-a-GCS |
| MFI | TBNK | Blue.670.LP.TE | CD45 on T cell | GCST90001915 | ebi-a-GCS |
| MFI | TBNK | Blue.670.LP.TE | CD45 on CD4+ | GCST90001916 | ebi-a-GCS |
| MFI | TBNK | Blue.670.LP.TE | CD45 on CD8br | GCST90001917 | ebi-a-GCS |
| MFI | TBNK | Blue.670.LP.TE | CD45 on HLA D | GCST90001918 | ebi-a-GCS |
| MFI | TBNK | Blue.670.LP.TE | CD45 on NKT | GCST90001919 | ebi-a-GCS |
| MFI | TBNK | Blue.670.LP.TE | CD45 on HLA D | GCST90001920 | ebi-a-GCS |
| MFI | TBNK | Blue.670.LP.TE | CD45 on HLA D | GCST90001921 | ebi-a-GCS |
| MFI | Treg | Blue.670.LP.Tr | CD127 on T cell | GCST90001922 | ebi-a-GCS |
| MFI | Treg | Blue.670.LP.Tr | CD127 on CD45 | GCST90001923 | ebi-a-GCS |
| MFI | Treg | Blue.670.LP.Tr | CD127 on CD28 | GCST90001924 | ebi-a-GCS |
| MFI | Treg | Blue.670.LP.Tr | CD127 on CD28 | GCST90001925 | ebi-a-GCS |
| MFI | Treg | Blue.670.LP.Tr | CD127 on granu | GCST90001926 | ebi-a-GCS |
| MFI | Treg | Blue.670.LP.Tr | CD127 on CD8b | GCST90001927 | ebi-a-GCS |
| MFI | Treg | Blue.670.LP.Tr | CD127 on CD28 | GCST90001928 | ebi-a-GCS |
| MFI | Treg | Blue.670.LP.Tr | CD127 on CD28 | GCST90001929 | ebi-a-GCS |

| | | | | | |
|---|---|---|---|---|---|
| MFI | Treg | Blue.670.LP.Tr | CD127 on CD2{ | GCST90001930 | ebi-a-GC{ |
| MFI | Treg | Blue.670.LP.Tr | CD127 on CD4+ | GCST90001931 | ebi-a-GC{ |
| MFI | Treg | Blue.670.LP.Tr | CD127 on CD4{ | GCST90001932 | ebi-a-GC{ |
| MFI | Treg | Blue.780.60.CI | CD25 on CD45 | GCST90001933 | ebi-a-GC{ |
| MFI | Treg | Blue.780.60.CI | CD25 on CD45I | GCST90001934 | ebi-a-GC{ |
| MFI | Treg | Blue.780.60.CI | CD25 on CD39+ | GCST90001935 | ebi-a-GC{ |
| MFI | Treg | Blue.780.60.CI | CD25 on CD4 T | GCST90001936 | ebi-a-GC{ |
| MFI | Treg | Blue.780.60.CI | CD25 on resting | GCST90001937 | ebi-a-GC{ |
| MFI | Treg | Blue.780.60.CI | CD25 on CD39+ | GCST90001938 | ebi-a-GC{ |
| MFI | Treg | Blue.780.60.CI | CD25 on activat | GCST90001939 | ebi-a-GC{ |
| MFI | Treg | Blue.780.60.CI | CD25 on CD39+ | GCST90001940 | ebi-a-GC{ |
| MFI | Treg | Blue.780.60.CI | CD25 on secreti | GCST90001941 | ebi-a-GC{ |
| MFI | Treg | Blue.780.60.CI | CD25 on CD39+ | GCST90001942 | ebi-a-GC{ |
| MFI | Treg | Blue.780.60.CI | CD25 on activat | GCST90001943 | ebi-a-GC{ |
| MFI | cDC | Blue.780.60.cD | CD123 on plasn | GCST90001944 | ebi-a-GC{ |
| MFI | cDC | Blue.780.60.cD | CD123 on CD6{ | GCST90001945 | ebi-a-GC{ |
| MFI | Myeloid c | Blue.780.60.MI | CD33 on CD14+ | GCST90001946 | ebi-a-GC{ |
| MFI | Myeloid c | Blue.780.60.MI | CD33 on CD33t | GCST90001947 | ebi-a-GC{ |
| MFI | Myeloid c | Blue.780.60.MI | CD33 on CD33c | GCST90001948 | ebi-a-GC{ |
| MFI | Myeloid c | Blue.780.60.MI | CD33 on CD33c | GCST90001949 | ebi-a-GC{ |
| MFI | Myeloid c | Blue.780.60.MI | CD33 on Gr MD | GCST90001950 | ebi-a-GC{ |
| MFI | Myeloid c | Blue.780.60.MI | CD33 on CD66t | GCST90001951 | ebi-a-GC{ |
| MFI | Myeloid c | Blue.780.60.MI | CD33 on Mo MI | GCST90001952 | ebi-a-GC{ |
| MFI | Myeloid c | Blue.780.60.MI | CD33 on CD33c | GCST90001953 | ebi-a-GC{ |
| MFI | Myeloid c | Blue.780.60.MI | CD33 on basopl | GCST90001954 | ebi-a-GC{ |
| MFI | Myeloid c | Blue.780.60.MI | CD33 on Im MD | GCST90001955 | ebi-a-GC{ |
| MFI | Myeloid c | Blue.780.60.MI | CD33 on CD33t | GCST90001956 | ebi-a-GC{ |
| MFI | Myeloid c | Blue.780.60.MI | CD33 on CD33t | GCST90001957 | ebi-a-GC{ |
| MFI | TBNK | Blue.780.60.TE | CD4 on monocy | GCST90001958 | ebi-a-GC{ |
| MFI | TBNK | Blue.780.60.TE | CD4 on HLA DF | GCST90001959 | ebi-a-GC{ |
| MFI | Treg | Blue.780.60.Tr | CD25 on CD4+ | GCST90001960 | ebi-a-GC{ |
| MFI | Treg | Blue.780.60.Tr | CD25 on CD39+ | GCST90001961 | ebi-a-GC{ |
| MFI | Treg | Blue.780.60.Tr | CD25 on CD28+ | GCST90001962 | ebi-a-GC{ |
| Morphological paramete | cDC | FSC.cDC.trait2 | FSC-A on myel | GCST90001963 | ebi-a-GC{ |
| Morphological paramete | cDC | FSC.cDC.trait3 | FSC-A on plasn | GCST90001964 | ebi-a-GC{ |
| Morphological paramete | cDC | FSC.cDC.trait4 | FSC-A on mono | GCST90001965 | ebi-a-GC{ |
| Morphological paramete | cDC | FSC.cDC.trait5 | FSC-A on granu | GCST90001966 | ebi-a-GC{ |
| Morphological paramete | TBNK | FSC.TBNK.trai | FSC-A on CD14 | GCST90001967 | ebi-a-GC{ |
| Morphological paramete | TBNK | FSC.TBNK.trai | FSC-A on B cell | GCST90001968 | ebi-a-GC{ |
| Morphological paramete | TBNK | FSC.TBNK.trai | FSC-A on NK | GCST90001969 | ebi-a-GC{ |
| Morphological paramete | TBNK | FSC.TBNK.trai | FSC-A on HLA | GCST90001970 | ebi-a-GC{ |
| Morphological paramete | TBNK | FSC.TBNK.trai | FSC-A on lymph | GCST90001971 | ebi-a-GC{ |
| Morphological paramete | TBNK | FSC.TBNK.trai | FSC-A on T cell | GCST90001972 | ebi-a-GC{ |
| Morphological paramete | TBNK | FSC.TBNK.trai | FSC-A on CD4+ | GCST90001973 | ebi-a-GC{ |
| Morphological paramete | TBNK | FSC.TBNK.trai | FSC-A on CD8t | GCST90001974 | ebi-a-GC{ |

| | | | | | |
|---|---|---|---|---|---|
| Morphological paramete | TBNK | FSC.TBNK.trai | FSC-A on HLA I | GCST90001975 | ebi-a-GCS |
| Morphological paramete | TBNK | FSC.TBNK.trai | FSC-A on NKT | GCST90001976 | ebi-a-GCS |
| Morphological paramete | TBNK | FSC.TBNK.trai | FSC-A on HLA | GCST90001977 | ebi-a-GCS |
| Morphological paramete | TBNK | FSC.TBNK.trai | FSC-A on HLA | GCST90001978 | ebi-a-GCS |
| MFI | Monocyte | mono.trait1 | CD16 on CD14- | GCST90001979 | ebi-a-GCS |
| MFI | Monocyte | mono.trait10 | CD40 on CD14+ | GCST90001980 | ebi-a-GCS |
| MFI | Monocyte | mono.trait11 | CD40 on CD14+ | GCST90001981 | ebi-a-GCS |
| MFI | Monocyte | mono.trait12 | CCR2 on CD14 | GCST90001982 | ebi-a-GCS |
| MFI | Monocyte | mono.trait14 | CD14 on CD14+ | GCST90001983 | ebi-a-GCS |
| MFI | Monocyte | mono.trait16 | HLA DR on CD1 | GCST90001984 | ebi-a-GCS |
| MFI | Monocyte | mono.trait17 | CD40 on monoc | GCST90001985 | ebi-a-GCS |
| MFI | Monocyte | mono.trait19 | CD14 on CD14+ | GCST90001986 | ebi-a-GCS |
| MFI | Monocyte | mono.trait2 | CD64 on CD14+ | GCST90001987 | ebi-a-GCS |
| MFI | Monocyte | mono.trait21 | HLA DR on CD1 | GCST90001988 | ebi-a-GCS |
| MFI | Monocyte | mono.trait22 | CD40 on CD14- | GCST90001989 | ebi-a-GCS |
| MFI | Monocyte | mono.trait24 | CD64 on CD14- | GCST90001990 | ebi-a-GCS |
| MFI | Monocyte | mono.trait25 | HLA DR on CD1 | GCST90001991 | ebi-a-GCS |
| MFI | Monocyte | mono.trait28 | CCR2 on CD14 | GCST90001992 | ebi-a-GCS |
| MFI | Monocyte | mono.trait29 | PDL-1 on CD14 | GCST90001993 | ebi-a-GCS |
| MFI | Monocyte | mono.trait3 | CX3CR1 on CD | GCST90001994 | ebi-a-GCS |
| MFI | Monocyte | mono.trait33 | CX3CR1 on mo | GCST90001995 | ebi-a-GCS |
| MFI | Monocyte | mono.trait35 | CX3CR1 on CD | GCST90001996 | ebi-a-GCS |
| MFI | Monocyte | mono.trait36 | CX3CR1 on CD | GCST90001997 | ebi-a-GCS |
| MFI | Monocyte | mono.trait39 | PDL-1 on CD14 | GCST90001998 | ebi-a-GCS |
| MFI | Monocyte | mono.trait4 | PDL-1 on CD14 | GCST90001999 | ebi-a-GCS |
| MFI | Monocyte | mono.trait41 | PDL-1 on CD14 | GCST90002000 | ebi-a-GCS |
| MFI | Monocyte | mono.trait42 | CD64 on CD14- | GCST90002001 | ebi-a-GCS |
| MFI | Monocyte | mono.trait43 | PDL-1 on mono | GCST90002002 | ebi-a-GCS |
| MFI | Monocyte | mono.trait44 | CCR2 on CD14 | GCST90002003 | ebi-a-GCS |
| MFI | Monocyte | mono.trait46 | CCR2 on CD14 | GCST90002004 | ebi-a-GCS |
| MFI | Monocyte | mono.trait47 | CD16 on CD14+ | GCST90002005 | ebi-a-GCS |
| MFI | Monocyte | mono.trait48 | CD64 on monoc | GCST90002006 | ebi-a-GCS |
| MFI | Monocyte | mono.trait50 | HLA DR on CD1 | GCST90002007 | ebi-a-GCS |
| MFI | Monocyte | mono.trait52 | CCR2 on monoc | GCST90002008 | ebi-a-GCS |
| MFI | Monocyte | mono.trait53 | HLA DR on CD1 | GCST90002009 | ebi-a-GCS |
| MFI | Monocyte | mono.trait54 | HLA DR on mor | GCST90002010 | ebi-a-GCS |
| MFI | Monocyte | mono.trait6 | CD64 on CD14+ | GCST90002011 | ebi-a-GCS |
| MFI | Monocyte | mono.trait7 | CX3CR1 on CD | GCST90002012 | ebi-a-GCS |
| MFI | cDC | Red.660.20.cD | CCR2 on myelo | GCST90002013 | ebi-a-GCS |
| MFI | cDC | Red.660.20.cD | CCR2 on CD62l | GCST90002014 | ebi-a-GCS |
| MFI | cDC | Red.660.20.cD | CCR2 on plasm | GCST90002015 | ebi-a-GCS |
| MFI | cDC | Red.660.20.cD | CCR2 on CD62l | GCST90002016 | ebi-a-GCS |
| MFI | cDC | Red.660.20.cD | CCR2 on monoc | GCST90002017 | ebi-a-GCS |
| MFI | cDC | Red.660.20.cD | CCR2 on granul | GCST90002018 | ebi-a-GCS |
| MFI | Myeloid c | Red.660.20.MI | CD14 on Mo MI | GCST90002019 | ebi-a-GCS |

| | | | | | |
|---|---|---|---|---|---|
| MFI | Myeloid c | Red.660.20.MI | CD14 on CD33k | GCST90002020 | ebi-a-GCS |
| MFI | Myeloid c | Red.660.20.MI | CD14 on CD33 | GCST90002021 | ebi-a-GCS |
| MFI | Maturatio | Red.660.20.M1 | CD4 on CD4+ | GCST90002022 | ebi-a-GCS |
| MFI | Maturatio | Red.660.20.M1 | CD4 on CM CD | GCST90002023 | ebi-a-GCS |
| MFI | Maturatio | Red.660.20.M1 | CD4 on naive C | GCST90002024 | ebi-a-GCS |
| MFI | Maturatio | Red.660.20.M1 | CD4 on EM CD | GCST90002025 | ebi-a-GCS |
| MFI | Maturatio | Red.660.20.M1 | CD4 on TD CD4 | GCST90002026 | ebi-a-GCS |
| MFI | Maturatio | Red.660.20.M1 | CD4 on CD45R | GCST90002027 | ebi-a-GCS |
| MFI | TBNK | Red.660.20.TB | CD19 on B cell | GCST90002028 | ebi-a-GCS |
| MFI | Treg | Red.660.20.Tr | CD39 on CD39 | GCST90002029 | ebi-a-GCS |
| MFI | Treg | Red.660.20.Tr | CD39 on CD39 | GCST90002030 | ebi-a-GCS |
| MFI | Treg | Red.660.20.Tr | CD39 on CD39 | GCST90002031 | ebi-a-GCS |
| MFI | Treg | Red.660.20.Tr | CD39 on CD39 | GCST90002032 | ebi-a-GCS |
| MFI | Treg | Red.660.20.Tr | CD39 on granul | GCST90002033 | ebi-a-GCS |
| MFI | Treg | Red.660.20.Tr | CD39 on monoc | GCST90002034 | ebi-a-GCS |
| MFI | cDC | Red.780.60.cD | CD80 on myeloi | GCST90002035 | ebi-a-GCS |
| MFI | cDC | Red.780.60.cD | CD80 on CD62L | GCST90002036 | ebi-a-GCS |
| MFI | cDC | Red.780.60.cD | CD80 on plasm | GCST90002037 | ebi-a-GCS |
| MFI | cDC | Red.780.60.cD | CD80 on CD62L | GCST90002038 | ebi-a-GCS |
| MFI | cDC | Red.780.60.cD | CD80 on monoc | GCST90002039 | ebi-a-GCS |
| MFI | cDC | Red.780.60.cD | CD80 on granul | GCST90002040 | ebi-a-GCS |
| MFI | Myeloid c | Red.780.60.MI | CD45 on lymph | GCST90002041 | ebi-a-GCS |
| MFI | Myeloid c | Red.780.60.MI | CD45 on CD33k | GCST90002042 | ebi-a-GCS |
| MFI | Myeloid c | Red.780.60.MI | CD45 on CD33k | GCST90002043 | ebi-a-GCS |
| MFI | Myeloid c | Red.780.60.MI | CD45 on CD33 | GCST90002044 | ebi-a-GCS |
| MFI | Myeloid c | Red.780.60.MI | CD45 on CD33- | GCST90002045 | ebi-a-GCS |
| MFI | Myeloid c | Red.780.60.MI | CD45 on CD33- | GCST90002046 | ebi-a-GCS |
| MFI | Myeloid c | Red.780.60.MI | CD45 on Gr MD | GCST90002047 | ebi-a-GCS |
| MFI | Myeloid c | Red.780.60.MI | CD45 on CD66k | GCST90002048 | ebi-a-GCS |
| MFI | Myeloid c | Red.780.60.MI | CD45 on Mo MI | GCST90002049 | ebi-a-GCS |
| MFI | Myeloid c | Red.780.60.MI | CD45 on CD33 | GCST90002050 | ebi-a-GCS |
| MFI | Myeloid c | Red.780.60.MI | CD45 on basop | GCST90002051 | ebi-a-GCS |
| MFI | Myeloid c | Red.780.60.MI | CD45 on Im MD | GCST90002052 | ebi-a-GCS |
| MFI | Myeloid c | Red.780.60.MI | CD45 on CD33k | GCST90002053 | ebi-a-GCS |
| MFI | Maturatio | Red.780.60.M1 | CD8 on CM CD | GCST90002054 | ebi-a-GCS |
| MFI | Maturatio | Red.780.60.M1 | CD8 on naive C | GCST90002055 | ebi-a-GCS |
| MFI | Maturatio | Red.780.60.M1 | CD8 on EM CD | GCST90002056 | ebi-a-GCS |
| MFI | Maturatio | Red.780.60.M1 | CD8 on TD CD | GCST90002057 | ebi-a-GCS |
| MFI | TBNK | Red.780.60.TB | CD8 on CD8br | GCST90002058 | ebi-a-GCS |
| MFI | TBNK | Red.780.60.TB | CD8 on NKT | GCST90002059 | ebi-a-GCS |
| MFI | TBNK | Red.780.60.TB | CD8 on HLA DF | GCST90002060 | ebi-a-GCS |
| MFI | Treg | Red.780.60.Tr | CD4 on CD39+ | GCST90002061 | ebi-a-GCS |
| MFI | Treg | Red.780.60.Tr | CD4 on CD28+ | GCST90002062 | ebi-a-GCS |
| MFI | Treg | Red.780.60.Tr | CD4 on CD4 Tr | GCST90002063 | ebi-a-GCS |
| MFI | Treg | Red.780.60.Tr | CD4 on resting | GCST90002064 | ebi-a-GCS |

| | | | | | |
|---|---|---|---|---|---|
| MFI | Treg | Red.780.60.Tre | CD4 on CD39+ | GCST90002065 | ebi-a-GCS |
| MFI | Treg | Red.780.60.Tre | CD4 on activate | GCST90002066 | ebi-a-GCS |
| MFI | Treg | Red.780.60.Tre | CD4 on CD39+ | GCST90002067 | ebi-a-GCS |
| MFI | Treg | Red.780.60.Tre | CD4 on secretin | GCST90002068 | ebi-a-GCS |
| MFI | Treg | Red.780.60.Tre | CD4 on CD39+ | GCST90002069 | ebi-a-GCS |
| MFI | Treg | Red.780.60.Tre | CD4 on activate | GCST90002070 | ebi-a-GCS |
| Morphological paramete | cDC | SSC.cDC.trait2 | SSC-A on myelo | GCST90002071 | ebi-a-GCS |
| Morphological paramete | cDC | SSC.cDC.trait3 | SSC-A on plasm | GCST90002072 | ebi-a-GCS |
| Morphological paramete | cDC | SSC.cDC.trait4 | SSC-A on mono | GCST90002073 | ebi-a-GCS |
| Morphological paramete | TBNK | SSC.TBNK.trai | SSC-A on CD14 | GCST90002074 | ebi-a-GCS |
| Morphological paramete | TBNK | SSC.TBNK.trai | SSC-A on B cell | GCST90002075 | ebi-a-GCS |
| Morphological paramete | TBNK | SSC.TBNK.trai | SSC-A on NK | GCST90002076 | ebi-a-GCS |
| Morphological paramete | TBNK | SSC.TBNK.trai | SSC-A on HLA | GCST90002077 | ebi-a-GCS |
| Morphological paramete | TBNK | SSC.TBNK.trai | SSC-A on granu | GCST90002078 | ebi-a-GCS |
| Morphological paramete | TBNK | SSC.TBNK.trai | SSC-A on lymph | GCST90002079 | ebi-a-GCS |
| Morphological paramete | TBNK | SSC.TBNK.trai | SSC-A on T cell | GCST90002080 | ebi-a-GCS |
| Morphological paramete | TBNK | SSC.TBNK.trai | SSC-A on CD4+ | GCST90002081 | ebi-a-GCS |
| Morphological paramete | TBNK | SSC.TBNK.trai | SSC-A on CD8b | GCST90002082 | ebi-a-GCS |
| Morphological paramete | TBNK | SSC.TBNK.trai | SSC-A on HLA I | GCST90002083 | ebi-a-GCS |
| Morphological paramete | TBNK | SSC.TBNK.trai | SSC-A on NKT | GCST90002084 | ebi-a-GCS |
| Morphological paramete | TBNK | SSC.TBNK.trai | SSC-A on HLA | GCST90002085 | ebi-a-GCS |
| Morphological paramete | TBNK | SSC.TBNK.trai | SSC-A on HLA | GCST90002086 | ebi-a-GCS |
| MFI | cDC | Violet.450.50.c | CD11c on myelo | GCST90002087 | ebi-a-GCS |
| MFI | cDC | Violet.450.50.c | CD11c on CD62 | GCST90002088 | ebi-a-GCS |
| MFI | cDC | Violet.450.50.c | CD11c on mono | GCST90002089 | ebi-a-GCS |
| MFI | cDC | Violet.450.50.c | CD11c on granu | GCST90002090 | ebi-a-GCS |
| MFI | Myeloid c | Violet.450.50.M | CD11b on CD14 | GCST90002091 | ebi-a-GCS |
| MFI | Myeloid c | Violet.450.50. | CD11b on Gr M | GCST90002092 | ebi-a-GCS |
| MFI | Myeloid c | Violet.450.50.M | CD11b on CD66 | GCST90002093 | ebi-a-GCS |
| MFI | Myeloid c | Violet.450.50.M | CD11b on Mo M | GCST90002094 | ebi-a-GCS |
| MFI | Myeloid c | Violet.450.50.M | CD11b on CD33 | GCST90002095 | ebi-a-GCS |
| MFI | Myeloid c | Violet.450.50.M | CD11b on baso | GCST90002096 | ebi-a-GCS |
| MFI | Myeloid c | Violet.450.50.M | CD11b on CD33 | GCST90002097 | ebi-a-GCS |
| MFI | Maturatio | Violet.450.50.M | CD45RA on nai | GCST90002098 | ebi-a-GCS |
| MFI | Maturatio | Violet.450.50.M | CD4RA on TD C | GCST90002099 | ebi-a-GCS |
| MFI | Maturatio | Violet.450.50.M | CD45RA on nai | GCST90002100 | ebi-a-GCS |
| MFI | Maturatio | Violet.450.50.M | CD45RA on TD | GCST90002101 | ebi-a-GCS |
| MFI | Treg | Violet.450.50.T | CD45RA on res | GCST90002102 | ebi-a-GCS |
| MFI | Treg | Violet.450.50.T | CD45RA on CD | GCST90002103 | ebi-a-GCS |
| MFI | cDC | Violet.510.50.c | HLA DR on mye | GCST90002104 | ebi-a-GCS |
| MFI | cDC | Violet.510.50.c | HLA DR on plas | GCST90002105 | ebi-a-GCS |
| MFI | cDC | Violet.510.50.c | HLA DR on DC | GCST90002106 | ebi-a-GCS |
| MFI | Myeloid c | Violet.510.50.M | HLA DR on HSC | GCST90002107 | ebi-a-GCS |
| MFI | Myeloid c | Violet.510.50.M | HLA DR on CD3 | GCST90002108 | ebi-a-GCS |
| MFI | Myeloid c | Violet.510.50.M | HLA DR on CD3 | GCST90002109 | ebi-a-GCS |

| | | | | | |
|---|---|---|---|---|---|
| MFI | Myeloid c | Violet.510.50.M | HLA DR on CD3 | GCST90002110 | ebi-a-GCS |
| MFI | Myeloid c | Violet.510.50.M | HLA DR on CD3 | GCST90002111 | ebi-a-GCS |
| MFI | Myeloid c | Violet.510.50.M | HLA DR on CD3 | GCST90002112 | ebi-a-GCS |
| MFI | TBNK | Violet.510.50.T | HLA DR on HLA | GCST90002113 | ebi-a-GCS |
| MFI | TBNK | Violet.510.50.T | HLA DR on HL | GCST90002114 | ebi-a-GCS |
| MFI | TBNK | Violet.510.50.T | HLA DR on HL | GCST90002115 | ebi-a-GCS |
| MFI | TBNK | Violet.510.50.T | HLA DR on B ce | GCST90002116 | ebi-a-GCS |
| MFI | TBNK | Violet.510.50.T | HLA DR on HLA | GCST90002117 | ebi-a-GCS |
| MFI | Treg | Violet.510.50.T | CD8 on CD28+ | GCST90002118 | ebi-a-GCS |
| MFI | Treg | Violet.510.50.T | CD8 on CD28+ | GCST90002119 | ebi-a-GCS |
| MFI | Treg | Violet.510.50.T | CD8 on CD28- ( | GCST90002120 | ebi-a-GCS |
| MFI | Treg | Violet.510.50.T | CD8 on CD39+ | GCST90002121 | ebi-a-GCS |

ST90001391
ST90001392
ST90001393
ST90001394
ST90001395
ST90001396
ST90001397
ST90001398
ST90001399
ST90001400
ST90001401
ST90001402
ST90001403
ST90001404
ST90001405
ST90001406
ST90001407
ST90001408
ST90001409
ST90001410
ST90001411
ST90001412
ST90001413
ST90001414
ST90001415
ST90001416
ST90001417
ST90001418
ST90001419
ST90001420
ST90001421
ST90001422
ST90001423
ST90001424
ST90001425
ST90001426
ST90001427
ST90001428
ST90001429
ST90001430
ST90001431
ST90001432
ST90001433
ST90001434

ST90001435
ST90001436
ST90001437
ST90001438
ST90001439
ST90001440
ST90001441
ST90001442
ST90001443
ST90001444
ST90001445
ST90001446
ST90001447
ST90001448
ST90001449
ST90001450
ST90001451
ST90001452
ST90001453
ST90001454
ST90001455
ST90001456
ST90001457
ST90001458
ST90001459
ST90001460
ST90001461
ST90001462
ST90001463
ST90001464
ST90001465
ST90001466
ST90001467
ST90001468
ST90001469
ST90001470
ST90001471
ST90001472
ST90001473
ST90001474
ST90001475
ST90001476
ST90001477
ST90001478
ST90001479

ST90001480
ST90001481
ST90001482
ST90001483
ST90001484
ST90001485
ST90001486
ST90001487
ST90001488
ST90001489
ST90001490
ST90001491
ST90001492
ST90001493
ST90001494
ST90001495
ST90001496
ST90001497
ST90001498
ST90001499
ST90001500
ST90001501
ST90001502
ST90001503
ST90001504
ST90001505
ST90001506
ST90001507
ST90001508
ST90001509
ST90001510
ST90001511
ST90001512
ST90001513
ST90001514
ST90001515
ST90001516
ST90001517
ST90001518
ST90001519
ST90001520
ST90001521
ST90001522
ST90001523
ST90001524

ST90001525
ST90001526
ST90001527
ST90001528
ST90001529
ST90001530
ST90001531
ST90001532
ST90001533
ST90001534
ST90001535
ST90001536
ST90001537
ST90001538
ST90001539
ST90001540
ST90001541
ST90001542
ST90001543
ST90001544
ST90001545
ST90001546
ST90001547
ST90001548
ST90001549
ST90001550
ST90001551
ST90001552
ST90001553
ST90001554
ST90001555
ST90001556
ST90001557
ST90001558
ST90001559
ST90001560
ST90001561
ST90001562
ST90001563
ST90001564
ST90001565
ST90001566
ST90001567
ST90001568
ST90001569

ST90001570
ST90001571
ST90001572
ST90001573
ST90001574
ST90001575
ST90001576
ST90001577
ST90001578
ST90001579
ST90001580
ST90001581
ST90001582
ST90001583
ST90001584
ST90001585
ST90001586
ST90001587
ST90001588
ST90001589
ST90001590
ST90001591
ST90001592
ST90001593
ST90001594
ST90001595
ST90001596
ST90001597
ST90001598
ST90001599
ST90001600
ST90001601
ST90001602
ST90001603
ST90001604
ST90001605
ST90001606
ST90001607
ST90001608
ST90001609
ST90001610
ST90001611
ST90001612
ST90001613
ST90001614

ST90001615
ST90001616
ST90001617
ST90001618
ST90001619
ST90001620
ST90001621
ST90001622
ST90001623
ST90001624
ST90001625
ST90001626
ST90001627
ST90001628
ST90001629
ST90001630
ST90001631
ST90001632
ST90001633
ST90001634
ST90001635
ST90001636
ST90001637
ST90001638
ST90001639
ST90001640
ST90001641
ST90001642
ST90001643
ST90001644
ST90001645
ST90001646
ST90001647
ST90001648
ST90001649
ST90001650
ST90001651
ST90001652
ST90001653
ST90001654
ST90001655
ST90001656
ST90001657
ST90001658
ST90001659

ST90001660
ST90001661
ST90001662
ST90001663
ST90001664
ST90001665
ST90001666
ST90001667
ST90001668
ST90001669
ST90001670
ST90001671
ST90001672
ST90001673
ST90001674
ST90001675
ST90001676
ST90001677
ST90001678
ST90001679
ST90001680
ST90001681
ST90001682
ST90001683
ST90001684
ST90001685
ST90001686
ST90001687
ST90001688
ST90001689
ST90001690
ST90001691
ST90001692
ST90001693
ST90001694
ST90001695
ST90001696
ST90001697
ST90001698
ST90001699
ST90001700
ST90001701
ST90001702
ST90001703
ST90001704

ST90001705
ST90001706
ST90001707
ST90001708
ST90001709
ST90001710
ST90001711
ST90001712
ST90001713
ST90001714
ST90001715
ST90001716
ST90001717
ST90001718
ST90001719
ST90001720
ST90001721
ST90001722
ST90001723
ST90001724
ST90001725
ST90001726
ST90001727
ST90001728
ST90001729
ST90001730
ST90001731
ST90001732
ST90001733
ST90001734
ST90001735
ST90001736
ST90001737
ST90001738
ST90001739
ST90001740
ST90001741
ST90001742
ST90001743
ST90001744
ST90001745
ST90001746
ST90001747
ST90001748
ST90001749

ST90001750
ST90001751
ST90001752
ST90001753
ST90001754
ST90001755
ST90001756
ST90001757
ST90001758
ST90001759
ST90001760
ST90001761
ST90001762
ST90001763
ST90001764
ST90001765
ST90001766
ST90001767
ST90001768
ST90001769
ST90001770
ST90001771
ST90001772
ST90001773
ST90001774
ST90001775
ST90001776
ST90001777
ST90001778
ST90001779
ST90001780
ST90001781
ST90001782
ST90001783
ST90001784
ST90001785
ST90001786
ST90001787
ST90001788
ST90001789
ST90001790
ST90001791
ST90001792
ST90001793
ST90001794

ST90001795
ST90001796
ST90001797
ST90001798
ST90001799
ST90001800
ST90001801
ST90001802
ST90001803
ST90001804
ST90001805
ST90001806
ST90001807
ST90001808
ST90001809
ST90001810
ST90001811
ST90001812
ST90001813
ST90001814
ST90001815
ST90001816
ST90001817
ST90001818
ST90001819
ST90001820
ST90001821
ST90001822
ST90001823
ST90001824
ST90001825
ST90001826
ST90001827
ST90001828
ST90001829
ST90001830
ST90001831
ST90001832
ST90001833
ST90001834
ST90001835
ST90001836
ST90001837
ST90001838
ST90001839

ST90001840
ST90001841
ST90001842
ST90001843
ST90001844
ST90001845
ST90001846
ST90001847
ST90001848
ST90001849
ST90001850
ST90001851
ST90001852
ST90001853
ST90001854
ST90001855
ST90001856
ST90001857
ST90001858
ST90001859
ST90001860
ST90001861
ST90001862
ST90001863
ST90001864
ST90001865
ST90001866
ST90001867
ST90001868
ST90001869
ST90001870
ST90001871
ST90001872
ST90001873
ST90001874
ST90001875
ST90001876
ST90001877
ST90001878
ST90001879
ST90001880
ST90001881
ST90001882
ST90001883
ST90001884

ST90001885
ST90001886
ST90001887
ST90001888
ST90001889
ST90001890
ST90001891
ST90001892
ST90001893
ST90001894
ST90001895
ST90001896
ST90001897
ST90001898
ST90001899
ST90001900
ST90001901
ST90001902
ST90001903
ST90001904
ST90001905
ST90001906
ST90001907
ST90001908
ST90001909
ST90001910
ST90001911
ST90001912
ST90001913
ST90001914
ST90001915
ST90001916
ST90001917
ST90001918
ST90001919
ST90001920
ST90001921
ST90001922
ST90001923
ST90001924
ST90001925
ST90001926
ST90001927
ST90001928
ST90001929

ST90001930
ST90001931
ST90001932
ST90001933
ST90001934
ST90001935
ST90001936
ST90001937
ST90001938
ST90001939
ST90001940
ST90001941
ST90001942
ST90001943
ST90001944
ST90001945
ST90001946
ST90001947
ST90001948
ST90001949
ST90001950
ST90001951
ST90001952
ST90001953
ST90001954
ST90001955
ST90001956
ST90001957
ST90001958
ST90001959
ST90001960
ST90001961
ST90001962
ST90001963
ST90001964
ST90001965
ST90001966
ST90001967
ST90001968
ST90001969
ST90001970
ST90001971
ST90001972
ST90001973
ST90001974

ST90001975
ST90001976
ST90001977
ST90001978
ST90001979
ST90001980
ST90001981
ST90001982
ST90001983
ST90001984
ST90001985
ST90001986
ST90001987
ST90001988
ST90001989
ST90001990
ST90001991
ST90001992
ST90001993
ST90001994
ST90001995
ST90001996
ST90001997
ST90001998
ST90001999
ST90002000
ST90002001
ST90002002
ST90002003
ST90002004
ST90002005
ST90002006
ST90002007
ST90002008
ST90002009
ST90002010
ST90002011
ST90002012
ST90002013
ST90002014
ST90002015
ST90002016
ST90002017
ST90002018
ST90002019

ST90002020
ST90002021
ST90002022
ST90002023
ST90002024
ST90002025
ST90002026
ST90002027
ST90002028
ST90002029
ST90002030
ST90002031
ST90002032
ST90002033
ST90002034
ST90002035
ST90002036
ST90002037
ST90002038
ST90002039
ST90002040
ST90002041
ST90002042
ST90002043
ST90002044
ST90002045
ST90002046
ST90002047
ST90002048
ST90002049
ST90002050
ST90002051
ST90002052
ST90002053
ST90002054
ST90002055
ST90002056
ST90002057
ST90002058
ST90002059
ST90002060
ST90002061
ST90002062
ST90002063
ST90002064

ST90002065
ST90002066
ST90002067
ST90002068
ST90002069
ST90002070
ST90002071
ST90002072
ST90002073
ST90002074
ST90002075
ST90002076
ST90002077
ST90002078
ST90002079
ST90002080
ST90002081
ST90002082
ST90002083
ST90002084
ST90002085
ST90002086
ST90002087
ST90002088
ST90002089
ST90002090
ST90002091
ST90002092
ST90002093
ST90002094
ST90002095
ST90002096
ST90002097
ST90002098
ST90002099
ST90002100
ST90002101
ST90002102
ST90002103
ST90002104
ST90002105
ST90002106
ST90002107
ST90002108
ST90002109

ST90002110
ST90002111
ST90002112
ST90002113
ST90002114
ST90002115
ST90002116
ST90002117
ST90002118
ST90002119
ST90002120
ST90002121

| id | p_val | avg_log2FC | pct.1 | pct.2 | p_val_adj |
|---|---|---|---|---|---|
| PKIB | 0 | 1.605195927 | 0.254 | 0.982 | 0 |
| WDR93 | 0 | 1.730581914 | 0.213 | 0.929 | 0 |
| CYP2J2 | 0 | -1.6361799 | 0.191 | 0.897 | 0 |
| RAMP3 | 0 | 1.379520522 | 0.254 | 0.955 | 0 |
| ITGB6 | 0 | 1.509924395 | 0.211 | 0.909 | 0 |
| LUM | 0 | -1.01268191 | 0.25 | 0.927 | 0 |
| STAB1 | 0 | 1.453998281 | 0.149 | 0.82 | 0 |
| AURKB | 0 | -1.56080098 | 0.201 | 0.87 | 0 |
| ZDHHC1 | 0 | -1.32218511 | 0.189 | 0.856 | 0 |
| SPATA17 | 0 | -1.65736688 | 0.225 | 0.89 | 0 |
| ERG | 0 | 2.131466905 | 0.235 | 0.899 | 0 |
| SDR16C5 | 0 | 1.62480221 | 0.157 | 0.817 | 0 |
| MT1G | 0 | -1.15735719 | 0.225 | 0.882 | 0 |
| KIF14 | 0 | -1.10724772 | 0.245 | 0.899 | 0 |
| SAXO2 | 0 | -1.07435027 | 0.24 | 0.883 | 0 |
| AC00826[illegible] | 0 | 1.392849294 | 0.118 | 0.757 | 0 |
| CLEC10A | 0 | -1.92984578 | 0.183 | 0.799 | 0 |
| DRD4 | 0 | 2.300659165 | 0.11 | 0.715 | 0 |
| UBE2C | 0 | -2.01673198 | 0.171 | 0.766 | 0 |
| CLEC4E | 0 | -1.00806779 | 0.11 | 0.666 | 0 |
| SLC15A2 | 0 | -1.90733124 | 0.088 | 0.626 | 0 |
| TNFAIP6 | 6.34E-306 | 1.064739409 | 0.164 | 0.792 | 1.27E-302 |
| CCL13 | 9.56E-277 | 1.098963669 | 0.026 | 0.427 | 1.91E-273 |
| ACOD1 | 6.91E-276 | 1.20029224 | 0.13 | 0.638 | 1.38E-272 |
| SPATA18 | 5.33E-275 | -1.23017035 | 0.088 | 0.608 | 1.07E-271 |
| TNFRSF[illegible] | 2.06E-263 | 1.082961259 | 0.404 | 0.965 | 4.12E-260 |
| DNAH12 | 6.42E-256 | 1.072312675 | 0.155 | 0.694 | 1.28E-252 |
| TIMP3 | 1.34E-240 | 1.709083677 | 0.272 | 0.894 | 2.68E-237 |
| GPRC5A | 5.88E-240 | 1.425860393 | 0.182 | 0.816 | 1.18E-236 |
| CLDN8 | 9.94E-238 | 3.115409319 | 0.074 | 0.484 | 1.99E-234 |
| RHOV | 4.52E-234 | 1.161337386 | 0.179 | 0.778 | 9.05E-231 |
| NPR3 | 8.00E-230 | -1.05030081 | 0.132 | 0.61 | 1.60E-226 |
| CFAP65 | 1.42E-229 | 1.921070423 | 0.147 | 0.625 | 2.84E-226 |
| PTPRB | 6.43E-227 | 1.508138236 | 0.312 | 0.894 | 1.29E-223 |
| MT1M | 1.91E-217 | -1.377514 | 0.041 | 0.407 | 3.81E-214 |
| SFTPA1 | 2.28E-216 | 1.490408465 | 0.17 | 0.798 | 4.55E-213 |
| SFTPB | 3.56E-216 | 1.065384534 | 0.148 | 0.776 | 7.13E-213 |
| TRBV14 | 1.45E-214 | 2.149842749 | 0.21 | 0.747 | 2.90E-211 |
| TNFSF15 | 1.46E-210 | 1.046899835 | 0.029 | 0.374 | 2.93E-207 |
| INMT | 3.33E-210 | -2.62661441 | 0.071 | 0.461 | 6.66E-207 |
| PTCRA | 2.64E-209 | 3.345670227 | 0.03 | 0.38 | 5.28E-206 |
| TCTEX1[illegible] | 5.70E-208 | -1.93479162 | 0.03 | 0.377 | 1.14E-204 |
| S100A14 | 5.25E-207 | 1.181799884 | 0.097 | 0.745 | 1.05E-203 |
| MAPK10 | 7.41E-207 | 1.038768267 | 0.104 | 0.536 | 1.48E-203 |

| | | | | | |
|---|---|---|---|---|---|
| TEKT2 | 2.68E-206 | -4.65006033 | 0.014 | 0.363 | 5.36E-203 |
| PKMYT1 | 3.25E-204 | -1.12728843 | 0.113 | 0.678 | 6.51E-201 |
| TNNT1 | 2.83E-202 | -3.49261173 | 0.03 | 0.394 | 5.66E-199 |
| ELN | 1.87E-197 | 1.039216518 | 0.22 | 0.805 | 3.73E-194 |
| HES4 | 8.83E-189 | 1.20989777 | 0.437 | 0.932 | 1.77E-185 |
| CD248 | 8.51E-183 | -1.52784071 | 0.119 | 0.546 | 1.70E-179 |
| SFTPA2 | 6.87E-182 | 1.488411875 | 0.185 | 0.755 | 1.37E-178 |
| DSP | 1.80E-179 | -2.73331691 | 0.073 | 0.474 | 3.61E-176 |
| DLGAP5 | 1.75E-169 | -1.6095868 | 0.036 | 0.34 | 3.50E-166 |
| FOXP3 | 7.49E-161 | -1.16746399 | 0.126 | 0.521 | 1.50E-157 |
| TRAV16 | 1.31E-160 | -1.2779743 | 0.239 | 0.798 | 2.63E-157 |
| TNXB | 3.33E-159 | 1.614444559 | 0.053 | 0.413 | 6.67E-156 |
| EFNB2 | 1.02E-155 | 1.00664035 | 0.344 | 0.874 | 2.05E-152 |
| HDC | 2.62E-155 | 1.058813715 | 0.133 | 0.569 | 5.24E-152 |
| HCAR3 | 1.20E-154 | -1.99440357 | 0.036 | 0.495 | 2.40E-151 |
| TNFRSF1 | 1.35E-153 | 1.191698135 | 0.282 | 0.836 | 2.70E-150 |
| ENPP2 | 1.84E-149 | 2.015083525 | 0.258 | 0.825 | 3.68E-146 |
| PLEKHH2 | 5.05E-143 | 1.305165534 | 0.102 | 0.509 | 1.01E-139 |
| TCIM | 4.63E-141 | 1.43920046 | 0.225 | 0.748 | 9.26E-138 |
| FBLN2 | 6.90E-141 | 1.183277929 | 0.241 | 0.808 | 1.38E-137 |
| IGKV3-15 | 2.43E-140 | -3.84333172 | 0.029 | 0.465 | 4.86E-137 |
| SIRPB1 | 2.23E-139 | -1.22288184 | 0.189 | 0.702 | 4.46E-136 |
| TPPP3 | 6.66E-139 | -1.05464021 | 0.274 | 0.781 | 1.33E-135 |
| HSPG2 | 7.83E-137 | 1.324624789 | 0.228 | 0.776 | 1.57E-133 |
| ICAM4 | 1.32E-135 | 1.0450949 | 0.09 | 0.569 | 2.63E-132 |
| RGS13 | 5.53E-133 | 1.261552659 | 0.108 | 0.717 | 1.11E-129 |
| LTC4S | 7.28E-132 | 1.326839509 | 0.227 | 0.768 | 1.46E-128 |
| MECOM | 5.18E-131 | 1.913009692 | 0.193 | 0.753 | 1.04E-127 |
| RAI14 | 1.27E-130 | 1.231188701 | 0.188 | 0.742 | 2.53E-127 |
| DNAJC5E | 1.74E-129 | -1.44261696 | 0.068 | 0.604 | 3.48E-126 |
| LAMP3 | 2.97E-127 | 1.085228604 | 0.278 | 0.779 | 5.94E-124 |
| IGFBP5 | 3.18E-123 | 1.226722038 | 0.123 | 0.681 | 6.35E-120 |
| TRDV2 | 2.63E-120 | -1.00290788 | 0.202 | 0.308 | 5.26E-117 |
| TNFRSF1 | 2.38E-119 | -6.35579968 | 0.079 | 0.411 | 4.76E-116 |
| SELENBF | 1.31E-118 | 1.039735504 | 0.084 | 0.68 | 2.61E-115 |
| TMEM232 | 2.70E-114 | -1.62558549 | 0.215 | 0.726 | 5.39E-111 |
| LDB2 | 6.79E-113 | 1.148546342 | 0.222 | 0.752 | 1.36E-109 |
| LRRC32 | 1.65E-112 | -1.4825376 | 0.062 | 0.405 | 3.30E-109 |
| DTX1 | 5.44E-111 | 1.188138976 | 0.191 | 0.651 | 1.09E-107 |
| CD34 | 2.46E-108 | -1.60216446 | 0.202 | 0.613 | 4.91E-105 |
| CFAP70 | 4.36E-107 | -1.63697407 | 0.14 | 0.632 | 8.73E-104 |
| NTRK1 | 2.04E-104 | 1.399861479 | 0.209 | 0.727 | 4.07E-101 |
| GATA2 | 6.83E-103 | 1.143373947 | 0.098 | 0.469 | 1.37E-99 |
| ELF3 | 1.53E-99 | 1.040777287 | 0.127 | 0.667 | 3.06E-96 |
| SEMA3C | 3.01E-99 | -2.47652973 | 0.109 | 0.653 | 6.03E-96 |

| | | | | | |
|---|---|---|---|---|---|
| RSPH4A | 7.95E-99 | 1.524605704 | 0.133 | 0.636 | 1.59E-95 |
| CFAP73 | 1.47E-98 | -2.54074904 | 0.076 | 0.468 | 2.94E-95 |
| CLIC5 | 4.21E-95 | -1.43592024 | 0.156 | 0.663 | 8.41E-92 |
| GTSE1 | 8.33E-94 | -1.32209571 | 0.147 | 0.634 | 1.67E-90 |
| RAMP2 | 9.05E-92 | 1.073093815 | 0.265 | 0.75 | 1.81E-88 |
| IGKV1-5 | 3.64E-90 | -3.00329649 | 0.069 | 0.513 | 7.29E-87 |
| TJP1 | 1.88E-89 | -2.95290431 | 0.028 | 0.261 | 3.76E-86 |
| TMEM176 | 5.25E-86 | 1.83170557 | 0.202 | 0.644 | 1.05E-82 |
| LCN2 | 1.01E-85 | -3.76511745 | 0.132 | 0.312 | 2.02E-82 |
| ITIH5 | 9.34E-84 | -1.4287535 | 0.01 | 0.254 | 1.87E-80 |
| TNFRSF4 | 1.59E-82 | 1.025101347 | 0.29 | 0.725 | 3.18E-79 |
| CNKSR3 | 2.71E-79 | -2.77322879 | 0.134 | 0.635 | 5.41E-76 |
| IGLV3-19 | 4.45E-79 | -2.85047026 | 0.024 | 0.418 | 8.90E-76 |
| CDC42EF | 3.98E-78 | 1.298393191 | 0.151 | 0.638 | 7.97E-75 |
| CKAP2L | 3.03E-76 | -1.04639501 | 0.068 | 0.441 | 6.07E-73 |
| ZNF750 | 3.81E-75 | -2.26975399 | 0.078 | 0.274 | 7.62E-72 |
| JAM2 | 1.21E-74 | 1.707679952 | 0.117 | 0.425 | 2.42E-71 |
| CENPA | 8.02E-74 | -1.88814942 | 0.132 | 0.563 | 1.60E-70 |
| IL3RA | 1.20E-72 | -1.64928165 | 0.213 | 0.685 | 2.40E-69 |
| OLFML3 | 3.70E-72 | 5.139112963 | 0.086 | 0.52 | 7.41E-69 |
| MFAP4 | 5.59E-72 | 2.07368502 | 0.1 | 0.537 | 1.12E-68 |
| EHD2 | 4.12E-71 | -1.23501781 | 0.015 | 0.489 | 8.24E-68 |
| TMEM45A | 2.74E-69 | -2.45825441 | 0.021 | 0.46 | 5.48E-66 |
| PDLIM3 | 3.68E-68 | 1.01403865 | 0.055 | 0.253 | 7.36E-65 |
| LRRC6 | 6.15E-68 | 1.139989765 | 0.025 | 0.255 | 1.23E-64 |
| RRAD | 4.37E-67 | 2.140559974 | 0.33 | 0.758 | 8.74E-64 |
| NPW | 1.28E-66 | -2.06068799 | 0.146 | 0.597 | 2.57E-63 |
| CCDC17 | 6.33E-66 | -1.11932875 | 0.132 | 0.342 | 1.27E-62 |
| LILRB5 | 5.42E-65 | 2.648166971 | 0.035 | 0.28 | 1.08E-61 |
| OAF | 9.77E-64 | 1.253222528 | 0.149 | 0.627 | 1.95E-60 |
| SPDEF | 1.84E-63 | -4.26930768 | 0.058 | 0.407 | 3.69E-60 |
| TRAV23D | 4.69E-60 | -1.68900373 | 0.269 | 0.715 | 9.38E-57 |
| ZNF683 | 1.83E-59 | -1.07744414 | 0.164 | 0.313 | 3.66E-56 |
| TRAV21 | 6.81E-58 | -1.3762189 | 0.17 | 0.64 | 1.36E-54 |
| MYLK | 4.59E-55 | 1.087696521 | 0.029 | 0.263 | 9.18E-52 |
| LILRB4 | 1.55E-54 | -1.36771418 | 0.086 | 0.261 | 3.11E-51 |
| PLK1 | 2.76E-54 | -1.53284999 | 0.144 | 0.635 | 5.52E-51 |
| TRAV13-1 | 3.78E-54 | -2.1752598 | 0.157 | 0.319 | 7.56E-51 |
| PPL | 3.04E-52 | 2.322514948 | 0.094 | 0.558 | 6.09E-49 |
| ALPL | 1.46E-51 | 2.252341626 | 0.021 | 0.506 | 2.91E-48 |
| TRAV8-4 | 8.20E-51 | -1.28104069 | 0.146 | 0.358 | 1.64E-47 |
| DEFB1 | 3.91E-49 | -4.22078243 | 0.03 | 0.395 | 7.82E-46 |
| VCAN | 6.38E-49 | -2.15807417 | 0.078 | 0.488 | 1.28E-45 |
| EPCAM | 6.28E-48 | -1.0623015 | 0.073 | 0.578 | 1.26E-44 |
| C4BPA | 3.20E-47 | 2.524466756 | 0.162 | 0.638 | 6.41E-44 |

| | | | | | |
|---|---|---|---|---|---|
| AARD | 4.13E-47 | 2.186353168 | 0.09 | 0.329 | 8.25E-44 |
| TRBV4-1 | 3.16E-46 | -1.17284873 | 0.279 | 0.68 | 6.32E-43 |
| MMRN2 | 1.21E-45 | -1.65538605 | 0.046 | 0.393 | 2.42E-42 |
| PODN | 2.30E-45 | -1.25842494 | 0.303 | 0.682 | 4.59E-42 |
| EGR4 | 5.65E-45 | 1.185219398 | 0.093 | 0.54 | 1.13E-41 |
| CNTD1 | 5.37E-44 | -2.13234381 | 0.054 | 0.345 | 1.07E-40 |
| MCAM | 2.97E-43 | -1.94663283 | 0.028 | 0.399 | 5.93E-40 |
| CTSK | 3.91E-43 | -1.35183456 | 0.197 | 0.625 | 7.81E-40 |
| MCTP1 | 4.30E-43 | 1.742963869 | 0.3 | 0.726 | 8.59E-40 |
| PRSS22 | 1.28E-41 | 1.783432986 | 0.185 | 0.656 | 2.55E-38 |
| CDCA2 | 1.85E-39 | -1.00052569 | 0.229 | 0.649 | 3.70E-36 |
| IGHA1 | 6.52E-39 | -2.36404085 | 0.115 | 0.588 | 1.30E-35 |
| MET | 9.84E-38 | 2.352847064 | 0.015 | 0.257 | 1.97E-34 |
| HCK | 2.89E-37 | -1.31637612 | 0.095 | 0.332 | 5.79E-34 |
| CFAP221 | 2.56E-36 | -1.32004344 | 0.071 | 0.341 | 5.12E-33 |
| KIF20A | 3.54E-35 | -5.26292554 | 0.031 | 0.262 | 7.08E-32 |
| PTGS2 | 5.29E-35 | 1.118491021 | 0.075 | 0.303 | 1.06E-31 |
| CFP | 1.30E-34 | -2.98607705 | 0.102 | 0.532 | 2.59E-31 |
| MMRN1 | 1.62E-33 | 1.324047398 | 0.073 | 0.397 | 3.23E-30 |
| PPIC | 5.40E-33 | -1.26979225 | 0.146 | 0.593 | 1.08E-29 |
| CLEC3B | 7.88E-33 | -1.28323764 | 0.084 | 0.358 | 1.58E-29 |
| BAMBI | 1.06E-32 | 1.032090826 | 0.287 | 0.666 | 2.13E-29 |
| MSMB | 4.73E-32 | 2.757905337 | 0.017 | 0.406 | 9.46E-29 |
| MNS1 | 4.76E-32 | 1.310590672 | 0.048 | 0.301 | 9.53E-29 |
| TNFRSF1 | 5.73E-32 | 1.071636382 | 0.277 | 0.646 | 1.15E-28 |
| NRARP | 1.38E-31 | 1.177775263 | 0.334 | 0.745 | 2.76E-28 |
| TRBV13 | 4.24E-31 | -2.99655353 | 0.114 | 0.333 | 8.48E-28 |
| TCN2 | 1.14E-30 | 1.116197819 | 0.064 | 0.523 | 2.29E-27 |
| SDS | 3.11E-30 | -1.79708808 | 0.019 | 0.282 | 6.22E-27 |
| AGRP | 1.91E-29 | -1.70592937 | 0.041 | 0.316 | 3.82E-26 |
| IL1A | 7.62E-28 | -1.28312503 | 0.101 | 0.36 | 1.52E-24 |
| CDS1 | 3.38E-27 | 1.243224699 | 0.149 | 0.606 | 6.75E-24 |
| KIF2C | 5.27E-27 | -1.11982299 | 0.092 | 0.52 | 1.05E-23 |
| NAPSA | 1.94E-26 | 1.83002664 | 0.112 | 0.489 | 3.87E-23 |
| TACC2 | 1.26E-25 | 1.522581754 | 0.083 | 0.434 | 2.51E-22 |
| TF | 2.13E-24 | -2.82044018 | 0.152 | 0.523 | 4.26E-21 |
| EMILIN1 | 2.55E-24 | -1.56727643 | 0.134 | 0.389 | 5.10E-21 |
| GPX3 | 1.50E-23 | 1.779717874 | 0.159 | 0.599 | 3.00E-20 |
| IGFBP6 | 3.39E-23 | 1.188589957 | 0.114 | 0.514 | 6.78E-20 |
| KRT8 | 3.76E-22 | 1.237571892 | 0.053 | 0.362 | 7.52E-19 |
| MUC4 | 6.70E-22 | -5.88999819 | 0.062 | 0.284 | 1.34E-18 |
| CCDC153 | 9.55E-22 | -1.45213177 | 0.084 | 0.357 | 1.91E-18 |
| CTLA4 | 1.19E-21 | -1.27631213 | 0.159 | 0.385 | 2.37E-18 |
| TNFRSF1 | 7.59E-21 | 2.042562293 | 0.19 | 0.593 | 1.52E-17 |
| KCNJ15 | 3.64E-20 | -1.54768335 | 0.113 | 0.545 | 7.28E-17 |

| | | | | | |
|---|---|---|---|---|---|
| MITF | 1.23E-19 | 1.14844359 | 0.136 | 0.56 | 2.45E-16 |
| CDKN1C | 1.65E-19 | 1.088991805 | 0.304 | 0.64 | 3.30E-16 |
| C7 | 2.09E-19 | 4.224242101 | 0.166 | 0.584 | 4.17E-16 |
| TPSB2 | 5.47E-19 | 1.270881413 | 0.144 | 0.548 | 1.09E-15 |
| SPARC | 1.37E-18 | 1.145474285 | 0.09 | 0.523 | 2.74E-15 |
| SOX17 | 2.41E-18 | -2.05859186 | 0.086 | 0.384 | 4.83E-15 |
| ANLN | 2.68E-18 | -1.53261632 | 0.137 | 0.419 | 5.36E-15 |
| S100B | 4.20E-18 | -2.09672046 | 0.233 | 0.457 | 8.40E-15 |
| KRT5 | 4.86E-18 | 1.387719563 | 0.033 | 0.476 | 9.72E-15 |
| RHPN2 | 6.79E-18 | 2.218909224 | 0.015 | 0.302 | 1.36E-14 |
| PDZK1IP | 1.58E-17 | 1.120802822 | 0.08 | 0.331 | 3.15E-14 |
| C11orf88 | 1.85E-17 | -1.50507974 | 0.002 | 0.332 | 3.70E-14 |
| SAA2 | 2.08E-17 | -2.33928942 | 0.082 | 0.376 | 4.16E-14 |
| APOC2 | 2.46E-17 | 1.058418226 | 0.249 | 0.627 | 4.92E-14 |
| OSCP1 | 2.59E-17 | 1.187462143 | 0.083 | 0.511 | 5.19E-14 |
| TPSAB1 | 7.21E-17 | 1.270229552 | 0.096 | 0.567 | 1.44E-13 |
| DNAH5 | 3.03E-16 | -3.5189622 | 0.009 | 0.41 | 6.05E-13 |
| FABP3 | 3.67E-16 | 2.403845954 | 0.082 | 0.376 | 7.33E-13 |
| IGKV3-20 | 3.88E-16 | -2.53996324 | 0.031 | 0.494 | 7.75E-13 |
| TRBV29- | 3.90E-16 | 1.575734319 | 0.212 | 0.579 | 7.80E-13 |
| TACSTD | 9.08E-16 | -1.35188541 | 0.074 | 0.365 | 1.82E-12 |
| TRBV3-1 | 1.23E-15 | -1.70827549 | 0.152 | 0.384 | 2.46E-12 |
| DUOX1 | 2.12E-15 | 1.119681761 | 0.185 | 0.467 | 4.25E-12 |
| BCAM | 2.61E-15 | 1.088173404 | 0.021 | 0.294 | 5.21E-12 |
| ABCA1 | 4.87E-15 | 1.466981522 | 0.102 | 0.502 | 9.74E-12 |
| CYP27A1 | 5.23E-15 | 1.86874162 | 0.128 | 0.355 | 1.05E-11 |
| FAM183A | 5.27E-15 | -1.99018455 | 0.048 | 0.446 | 1.05E-11 |
| IGHA2 | 2.02E-14 | -2.14051845 | 0.001 | 0.259 | 4.04E-11 |
| DNAAF1 | 2.76E-14 | -1.24080665 | 0.028 | 0.323 | 5.52E-11 |
| PLAU | 3.74E-14 | 1.397298097 | 0.147 | 0.568 | 7.47E-11 |
| CCL2 | 4.01E-14 | 1.399979599 | 0.076 | 0.357 | 8.02E-11 |
| CAVIN1 | 6.33E-14 | 2.166608852 | 0.112 | 0.385 | 1.27E-10 |
| MACC1 | 1.24E-13 | 1.752263681 | 0.097 | 0.379 | 2.48E-10 |
| C11orf96 | 1.83E-13 | 1.465640842 | 0.13 | 0.509 | 3.67E-10 |
| FBXO27 | 2.23E-13 | 1.261143488 | 0.094 | 0.366 | 4.47E-10 |
| SFTPC | 2.46E-13 | 1.186965369 | 0.483 | 0.951 | 4.91E-10 |
| MMP7 | 3.03E-13 | 2.369317693 | 0.074 | 0.396 | 6.06E-10 |
| TRBV12- | 3.70E-13 | -1.42736337 | 0.143 | 0.375 | 7.40E-10 |
| DDR2 | 5.05E-13 | -1.51017965 | 0.22 | 0.572 | 1.01E-09 |
| BGN | 4.29E-12 | 1.382239195 | 0.005 | 0.251 | 8.59E-09 |
| IGKV4-1 | 4.69E-12 | -1.71723249 | 0.111 | 0.513 | 9.38E-09 |
| TSPAN6 | 7.73E-12 | 2.962432363 | 0.019 | 0.425 | 1.55E-08 |
| TRBV18 | 2.75E-11 | -1.0455596 | 0.168 | 0.556 | 5.49E-08 |
| TRBV7-2 | 6.39E-11 | -2.0355023 | 0.107 | 0.397 | 1.28E-07 |
| IGSF10 | 6.85E-11 | 4.88282292 | 0.105 | 0.523 | 1.37E-07 |

| | | | | | |
|---|---|---|---|---|---|
| IGHV4-59 | 1.45E-10 | -3.67616171 | 0 | 0.306 | 2.90E-07 |
| IGHG4 | 5.39E-10 | -2.00620797 | 0.068 | 0.329 | 1.08E-06 |
| TSPAN1 | 1.12E-09 | -3.20888228 | 0.08 | 0.37 | 2.25E-06 |
| CPB2 | 6.53E-09 | -1.35782964 | 0.015 | 0.269 | 1.31E-05 |
| LTF | 9.25E-09 | -1.7982814 | 0.199 | 0.558 | 1.85E-05 |
| PODXL | 1.09E-08 | -1.29799774 | 0.043 | 0.324 | 2.18E-05 |
| FHAD1 | 1.95E-08 | -1.31602636 | 0.115 | 0.432 | 3.91E-05 |
| CTSG | 1.97E-08 | 1.275806198 | 0.053 | 0.302 | 3.95E-05 |
| TSNAXIP | 2.04E-08 | -2.84640596 | 0.089 | 0.396 | 4.08E-05 |
| THBS1 | 2.18E-08 | 1.177728666 | 0.295 | 0.595 | 4.35E-05 |
| STEAP4 | 5.50E-08 | 1.31626627 | 0.104 | 0.35 | 0.00010993194 |
| CXCL5 | 7.98E-08 | -2.40980337 | 0.064 | 0.373 | 0.00015968088 |
| RSPO3 | 9.67E-08 | 1.218896033 | 0.024 | 0.359 | 0.00019330038 |
| AKAP12 | 1.22E-07 | 1.68314571 | 0.049 | 0.458 | 0.00024399579 |
| CLDN4 | 1.63E-07 | 1.420083476 | 0.13 | 0.525 | 0.0003252675 |
| SPAG17 | 2.78E-07 | -1.14007401 | 0.016 | 0.261 | 0.00055577707 |
| VEGFA | 2.80E-07 | 1.418639788 | 0.101 | 0.517 | 0.00056029417 |
| CPM | 5.53E-07 | 2.008483991 | 0.118 | 0.517 | 0.00110584245 |
| ELN-AS1 | 7.94E-07 | 1.295969308 | 0.189 | 0.47 | 0.00158880416 |
| SNTN | 8.86E-07 | -2.40388606 | 0.045 | 0.332 | 0.00177253808 |
| KRT18 | 2.36E-06 | 1.510460265 | 0.157 | 0.525 | 0.00472676593 |
| JCAD | 3.39E-06 | -1.22482317 | 0.033 | 0.348 | 0.00678888517 |
| RSPH1 | 4.05E-06 | 1.36626142 | 0.074 | 0.397 | 0.00809964036 |
| IGLV2-14 | 4.88E-06 | -1.39848731 | 0.24 | 0.494 | 0.00975867242 |
| BHLHE41 | 5.08E-06 | 1.53252005 | 0.089 | 0.356 | 0.01015632317 |
| CXCL9 | 5.28E-06 | -3.49196398 | 0.093 | 0.431 | 0.01055411624 |
| MSR1 | 6.72E-06 | 1.34880672 | 0.101 | 0.344 | 0.01343116265 |
| MMP14 | 1.86E-05 | -2.11098262 | 0.072 | 0.436 | 0.03722335207 |
| CYP4B1 | 2.35E-05 | -1.83097345 | 0.156 | 0.538 | 0.04698889608 |

| id | nsnp | method | b | se | or | or_lci95 | or_uci95 | pvalue |
|---|---|---|---|---|---|---|---|---|
| ebi-a-GCST9000139 | 8 | Inverse va | -0.05937 | 0.019601 | 0.942363 | 0.906846 | 0.97927 | 0.002456 |
| ebi-a-GCST9000143 | 3 | Inverse va | -0.08217 | 0.031496 | 0.921114 | 0.865971 | 0.979768 | 0.009082 |
| ebi-a-GCST9000145 | 5 | Inverse va | -0.10033 | 0.031654 | 0.904536 | 0.850123 | 0.962432 | 0.001526 |
| ebi-a-GCST9000145 | 19 | Inverse va | 0.058004 | 0.014455 | 1.059719 | 1.030117 | 1.090172 | 6.00E-05 |
| ebi-a-GCST9000146 | 15 | Inverse va | 0.059749 | 0.015522 | 1.06157 | 1.02976 | 1.094363 | 0.000118 |
| ebi-a-GCST9000146 | 25 | Inverse va | 0.062272 | 0.011303 | 1.064252 | 1.040935 | 1.088091 | 3.60E-08 |
| ebi-a-GCST9000146 | 25 | Inverse va | 0.062272 | 0.011975 | 1.064251 | 1.039562 | 1.089527 | 1.99E-07 |
| ebi-a-GCST9000146 | 27 | Inverse va | 0.039579 | 0.01214 | 1.040373 | 1.015911 | 1.065424 | 0.001113 |
| ebi-a-GCST9000146 | 26 | Inverse va | 0.041506 | 0.012248 | 1.04238 | 1.017654 | 1.067706 | 0.000702 |
| ebi-a-GCST9000146 | 3 | Inverse va | -0.12999 | 0.044748 | 0.878108 | 0.804373 | 0.958602 | 0.003674 |
| ebi-a-GCST9000146 | 29 | Inverse va | 0.057029 | 0.010821 | 1.058687 | 1.036468 | 1.081381 | 1.36E-07 |
| ebi-a-GCST9000146 | 30 | Inverse va | 0.052343 | 0.010678 | 1.053737 | 1.031913 | 1.076022 | 9.48E-07 |
| ebi-a-GCST9000147 | 36 | Inverse va | 0.041014 | 0.009358 | 1.041866 | 1.022932 | 1.061152 | 1.17E-05 |
| ebi-a-GCST9000147 | 35 | Inverse va | 0.040643 | 0.00963 | 1.04148 | 1.022006 | 1.061326 | 2.44E-05 |
| ebi-a-GCST9000152 | 5 | Inverse va | 0.064828 | 0.022439 | 1.066975 | 1.021066 | 1.114949 | 0.003864 |
| ebi-a-GCST9000152 | 5 | Inverse va | -0.05348 | 0.018777 | 0.947924 | 0.913671 | 0.983461 | 0.004398 |
| ebi-a-GCST9000152 | 5 | Inverse va | 0.053875 | 0.018698 | 1.055352 | 1.017375 | 1.094747 | 0.003961 |
| ebi-a-GCST9000153 | 14 | Inverse va | 0.063555 | 0.011445 | 1.065618 | 1.04198 | 1.089792 | 2.81E-08 |
| ebi-a-GCST9000157 | 9 | Inverse va | -0.08999 | 0.03379 | 0.913937 | 0.855369 | 0.976516 | 0.007738 |
| ebi-a-GCST9000158 | 21 | Inverse va | -0.06973 | 0.014777 | 0.93265 | 0.906025 | 0.960057 | 2.38E-06 |
| ebi-a-GCST9000158 | 3 | Inverse va | 0.149661 | 0.049716 | 1.161441 | 1.053605 | 1.280312 | 0.00261 |
| ebi-a-GCST9000158 | 9 | Inverse va | -0.08687 | 0.033793 | 0.916797 | 0.858041 | 0.979577 | 0.010152 |
| ebi-a-GCST9000158 | 20 | Inverse va | -0.06067 | 0.013436 | 0.941131 | 0.91667 | 0.966245 | 6.31E-06 |
| ebi-a-GCST9000158 | 9 | Inverse va | 0.0806 | 0.02833 | 1.083937 | 1.02539 | 1.145827 | 0.004441 |
| ebi-a-GCST9000158 | 10 | Inverse va | -0.08501 | 0.027065 | 0.918502 | 0.871048 | 0.968542 | 0.001684 |
| ebi-a-GCST9000159 | 6 | Inverse va | 0.120078 | 0.051311 | 1.127585 | 1.019701 | 1.246883 | 0.019272 |
| ebi-a-GCST9000161 | 7 | Inverse va | 0.072328 | 0.023552 | 1.075008 | 1.026512 | 1.125796 | 0.002133 |
| ebi-a-GCST9000162 | 12 | Inverse va | 0.057755 | 0.022923 | 1.059455 | 1.012908 | 1.108141 | 0.011753 |
| ebi-a-GCST9000162 | 9 | Inverse va | 0.091163 | 0.037375 | 1.095448 | 1.018068 | 1.178708 | 0.014723 |
| ebi-a-GCST9000163 | 3 | Inverse va | 0.147809 | 0.045475 | 1.159292 | 1.060434 | 1.267366 | 0.001153 |
| ebi-a-GCST9000164 | 21 | Inverse va | -0.0504 | 0.015253 | 0.950849 | 0.922843 | 0.979704 | 0.000952 |
| ebi-a-GCST9000164 | 24 | Inverse va | -0.05177 | 0.014161 | 0.949546 | 0.923554 | 0.97627 | 0.000256 |
| ebi-a-GCST9000165 | 24 | Inverse va | -0.04562 | 0.014199 | 0.955405 | 0.929182 | 0.982368 | 0.001314 |
| ebi-a-GCST9000170 | 128 | Inverse va | -0.01246 | 0.005203 | 0.987621 | 0.9776 | 0.997744 | 0.016661 |
| ebi-a-GCST9000170 | 124 | Inverse va | -0.01096 | 0.005276 | 0.989098 | 0.978923 | 0.999379 | 0.037731 |
| ebi-a-GCST9000170 | 118 | Inverse va | -0.01309 | 0.00541 | 0.986999 | 0.976587 | 0.997521 | 0.01557 |
| ebi-a-GCST9000170 | 59 | Inverse va | -0.01272 | 0.006152 | 0.987359 | 0.975525 | 0.999337 | 0.038663 |
| ebi-a-GCST9000170 | 123 | Inverse va | -0.01171 | 0.00544 | 0.988361 | 0.977878 | 0.998957 | 0.031407 |
| ebi-a-GCST9000171 | 109 | Inverse va | -0.01228 | 0.006038 | 0.987794 | 0.976172 | 0.999553 | 0.041952 |
| ebi-a-GCST9000171 | 112 | Inverse va | -0.01193 | 0.005871 | 0.988139 | 0.976834 | 0.999575 | 0.042104 |
| ebi-a-GCST9000171 | 127 | Inverse va | -0.01184 | 0.005363 | 0.988227 | 0.977894 | 0.998669 | 0.027219 |
| ebi-a-GCST9000171 | 11 | Inverse va | -0.08353 | 0.029152 | 0.919861 | 0.868775 | 0.973951 | 0.004165 |
| ebi-a-GCST9000171 | 128 | Inverse va | -0.01299 | 0.005259 | 0.987092 | 0.976969 | 0.997319 | 0.013497 |
| ebi-a-GCST9000171 | 116 | Inverse va | -0.01159 | 0.005749 | 0.988481 | 0.977406 | 0.999682 | 0.043867 |
| ebi-a-GCST9000171 | 127 | Inverse va | -0.01171 | 0.005257 | 0.988354 | 0.978222 | 0.99859 | 0.02585 |
| ebi-a-GCST9000171 | 125 | Inverse va | -0.01146 | 0.005533 | 0.988606 | 0.977943 | 0.999385 | 0.038339 |
| ebi-a-GCST9000171 | 122 | Inverse va | -0.01133 | 0.00542 | 0.988732 | 0.978285 | 0.99929 | 0.036528 |

| | | | | | | | | |
|---|---|---|---|---|---|---|---|---|
| ebi-a-GCST9000172 | 97 | Inverse va | -0.01784 | 0.005786 | 0.98232 | 0.971242 | 0.993525 | 0.002051 |
| ebi-a-GCST9000174 | 10 | Inverse va | -0.07213 | 0.024495 | 0.930411 | 0.886797 | 0.97617 | 0.003233 |
| ebi-a-GCST9000174 | 8 | Inverse va | -0.06099 | 0.024784 | 0.940829 | 0.896218 | 0.98766 | 0.013855 |
| ebi-a-GCST9000175 | 17 | Inverse va | -0.05216 | 0.019538 | 0.949174 | 0.913514 | 0.986226 | 0.007588 |
| ebi-a-GCST9000175 | 10 | Inverse va | -0.05174 | 0.022727 | 0.949574 | 0.908205 | 0.992828 | 0.022804 |
| ebi-a-GCST9000176 | 9 | Inverse va | -0.05525 | 0.023882 | 0.946252 | 0.90298 | 0.991597 | 0.020703 |
| ebi-a-GCST9000179 | 34 | Inverse va | 0.025804 | 0.011302 | 1.02614 | 1.00366 | 1.049124 | 0.022416 |
| ebi-a-GCST9000180 | 20 | Inverse va | 0.039346 | 0.012342 | 1.04013 | 1.015271 | 1.065597 | 0.001432 |
| ebi-a-GCST9000180 | 16 | Inverse va | 0.040166 | 0.010623 | 1.040984 | 1.019533 | 1.062886 | 0.000156 |
| ebi-a-GCST9000180 | 24 | Inverse va | 0.042846 | 0.013383 | 1.043777 | 1.016753 | 1.071519 | 0.001367 |
| ebi-a-GCST9000180 | 31 | Inverse va | 0.030222 | 0.01121 | 1.030684 | 1.008284 | 1.053581 | 0.007019 |
| ebi-a-GCST9000180 | 24 | Inverse va | 0.037616 | 0.013313 | 1.038332 | 1.011589 | 1.065783 | 0.004721 |
| ebi-a-GCST9000180 | 26 | Inverse va | 0.049522 | 0.0118 | 1.050768 | 1.026746 | 1.075353 | 2.71E-05 |
| ebi-a-GCST9000180 | 33 | Inverse va | 0.030011 | 0.01075 | 1.030466 | 1.008981 | 1.052408 | 0.005243 |
| ebi-a-GCST9000182 | 116 | Inverse va | -0.01297 | 0.005456 | 0.987113 | 0.976613 | 0.997727 | 0.017447 |
| ebi-a-GCST9000183 | 3 | Inverse va | -0.14905 | 0.055046 | 0.861528 | 0.773417 | 0.959677 | 0.006775 |
| ebi-a-GCST9000183 | 5 | Inverse va | 0.05214 | 0.022257 | 1.053523 | 1.008552 | 1.100499 | 0.01915 |
| ebi-a-GCST9000183 | 9 | Inverse va | 0.039313 | 0.017653 | 1.040096 | 1.004725 | 1.076712 | 0.025946 |
| ebi-a-GCST9000186 | 4 | Inverse va | 0.07008 | 0.035282 | 1.072594 | 1.000929 | 1.14939 | 0.046999 |
| ebi-a-GCST9000188 | 22 | Inverse va | 0.032971 | 0.013276 | 1.033521 | 1.006974 | 1.060768 | 0.013011 |
| ebi-a-GCST9000189 | 5 | Inverse va | -0.05922 | 0.022738 | 0.942496 | 0.901415 | 0.98545 | 0.009199 |
| ebi-a-GCST9000189 | 28 | Inverse va | -0.0277 | 0.009996 | 0.972684 | 0.953813 | 0.991928 | 0.005592 |
| ebi-a-GCST9000189 | 9 | Inverse va | -0.05209 | 0.017118 | 0.949239 | 0.917919 | 0.981627 | 0.00234 |
| ebi-a-GCST9000190 | 7 | Inverse va | 0.048534 | 0.024349 | 1.049731 | 1.00081 | 1.101043 | 0.046235 |
| ebi-a-GCST9000197 | 15 | Inverse va | -0.0675 | 0.017327 | 0.934728 | 0.903516 | 0.967018 | 9.80E-05 |
| ebi-a-GCST9000197 | 79 | Inverse va | -0.02333 | 0.006614 | 0.976942 | 0.964359 | 0.989689 | 0.00042 |
| ebi-a-GCST9000198 | 14 | Inverse va | -0.0478 | 0.01437 | 0.953326 | 0.926849 | 0.980559 | 0.00088 |
| ebi-a-GCST9000198 | 23 | Inverse va | -0.03797 | 0.01349 | 0.962743 | 0.937621 | 0.988539 | 0.004886 |
| ebi-a-GCST9000198 | 23 | Inverse va | -0.04681 | 0.012925 | 0.954273 | 0.930401 | 0.978757 | 0.000293 |
| ebi-a-GCST9000198 | 38 | Inverse va | -0.0366 | 0.01057 | 0.964058 | 0.944291 | 0.984239 | 0.000534 |
| ebi-a-GCST9000199 | 14 | Inverse va | 0.084478 | 0.019489 | 1.088149 | 1.047367 | 1.130518 | 1.46E-05 |
| ebi-a-GCST9000199 | 13 | Inverse va | 0.048505 | 0.017957 | 1.049701 | 1.013398 | 1.087304 | 0.00691 |
| ebi-a-GCST9000199 | 11 | Inverse va | 0.055097 | 0.018539 | 1.056643 | 1.018937 | 1.095745 | 0.00296 |
| ebi-a-GCST9000199 | 17 | Inverse va | 0.045836 | 0.016591 | 1.046903 | 1.013407 | 1.081506 | 0.005733 |
| ebi-a-GCST9000199 | 11 | Inverse va | -0.05899 | 0.02119 | 0.942719 | 0.904367 | 0.982697 | 0.005374 |
| ebi-a-GCST9000200 | 3 | Inverse va | 0.088548 | 0.034162 | 1.092586 | 1.021825 | 1.168248 | 0.009542 |
| ebi-a-GCST9000201 | 56 | Inverse va | -0.02558 | 0.011977 | 0.974741 | 0.952126 | 0.997893 | 0.032674 |
| ebi-a-GCST9000201 | 7 | Inverse va | 0.063899 | 0.023116 | 1.065984 | 1.018764 | 1.115393 | 0.005706 |
| ebi-a-GCST9000202 | 5 | Inverse va | -0.06572 | 0.02983 | 0.936389 | 0.88321 | 0.992769 | 0.027576 |
| ebi-a-GCST9000203 | 4 | Inverse va | -0.13655 | 0.042736 | 0.87236 | 0.802266 | 0.948578 | 0.001397 |
| ebi-a-GCST9000203 | 16 | Inverse va | -0.0401 | 0.013105 | 0.960692 | 0.936331 | 0.985687 | 0.002212 |
| ebi-a-GCST9000208 | 4 | Inverse va | -0.06627 | 0.033732 | 0.935874 | 0.876 | 0.99984 | 0.049445 |
| ebi-a-GCST9000209 | 4 | Inverse va | -0.05936 | 0.020026 | 0.942363 | 0.906092 | 0.980087 | 0.003033 |
| ebi-a-GCST9000211 | 40 | Inverse va | 0.023058 | 0.009803 | 1.023326 | 1.003852 | 1.043177 | 0.018663 |
| ebi-a-GCST9000211 | 41 | Inverse va | -0.03122 | 0.012731 | 0.969264 | 0.945378 | 0.993753 | 0.014197 |

| id | nsnp.x | method.x | b.x | se.x | or.x | or_lci95.x | or_uci95.x | pvalue.x | nsnp.y |
|---|---|---|---|---|---|---|---|---|---|
| ebi-a-GC | 9 | Inverse va | 0.172838 | 0.084012 | 1.188674 | 1.008208 | 1.401443 | 0.039657 | 3 |
| ebi-a-GC | 9 | Inverse va | 0.163208 | 0.079305 | 1.177282 | 1.007801 | 1.375264 | 0.039592 | 128 |
| ebi-a-GC | 9 | Inverse va | 0.159204 | 0.079211 | 1.172578 | 1.003959 | 1.369516 | 0.044443 | 128 |
| ebi-a-GC | 9 | Inverse va | 0.155524 | 0.079228 | 1.16827 | 1.000237 | 1.364532 | 0.049648 | 127 |
| ebi-a-GC | 9 | Inverse va | 0.171756 | 0.079379 | 1.187388 | 1.016305 | 1.38727 | 0.030484 | 125 |
| ebi-a-GC | 9 | Inverse va | 0.157801 | 0.077946 | 1.170934 | 1.00504 | 1.36421 | 0.042919 | 10 |
| ebi-a-GC | 9 | Inverse va | 0.178953 | 0.078711 | 1.195964 | 1.024987 | 1.395463 | 0.022993 | 23 |
| ebi-a-GC | 9 | Inverse va | 0.205356 | 0.079225 | 1.227963 | 1.051351 | 1.434242 | 0.00954 | 56 |
| ebi-a-GC | 9 | Inverse va | -0.22902 | 0.086362 | 0.795312 | 0.671466 | 0.941999 | 0.008005 | 5 |

| method.y | b.y | se.y | or.y | or_lci95.y | or_uci95.y | pvalue.y | beta_all | beta1 | beta2 |
|---|---|---|---|---|---|---|---|---|---|
| Inverse va | 0.149661 | 0.049716 | 1.161441 | 1.053605 | 1.280312 | 0.00261 | 0.085763 | 0.172838 | 0.149661 |
| Inverse va | -0.01246 | 0.005203 | 0.987621 | 0.9776 | 0.997744 | 0.016661 | 0.085763 | 0.163208 | -0.01246 |
| Inverse va | -0.01299 | 0.005259 | 0.987092 | 0.976969 | 0.997319 | 0.013497 | 0.085763 | 0.159204 | -0.01299 |
| Inverse va | -0.01171 | 0.005257 | 0.988354 | 0.978222 | 0.99859 | 0.02585 | 0.085763 | 0.155524 | -0.01171 |
| Inverse va | -0.01146 | 0.005533 | 0.988606 | 0.977943 | 0.999385 | 0.038339 | 0.085763 | 0.171756 | -0.01146 |
| Inverse va | -0.07213 | 0.024495 | 0.930411 | 0.886797 | 0.97617 | 0.003233 | 0.085763 | 0.157801 | -0.07213 |
| Inverse va | -0.03797 | 0.01349 | 0.962743 | 0.937621 | 0.988539 | 0.004886 | 0.085763 | 0.178953 | -0.03797 |
| Inverse va | -0.02558 | 0.011977 | 0.974741 | 0.952126 | 0.997893 | 0.032674 | 0.085763 | 0.205356 | -0.02558 |
| Inverse va | -0.06572 | 0.02983 | 0.936389 | 0.88321 | 0.992769 | 0.027576 | 0.085763 | -0.22902 | -0.06572 |

| beta12 | beta_dir | se | Z | P | lci | uci | beta12_p | lci_p | uci_p |
|---|---|---|---|---|---|---|---|---|---|
| 0.025867 | 0.059896 | 0.016316 | 1.585401 | 0.112875 | -0.00611 | 0.057846 | 0.301611 | -0.07126 | 0.674486 |
| -0.00203 | 0.087796 | 0.012943 | -0.15707 | 0.87519 | -0.0274 | 0.023336 | -0.0237 | -0.31951 | 0.272097 |
| -0.00207 | 0.087832 | 0.012611 | -0.16402 | 0.869718 | -0.02679 | 0.022649 | -0.02412 | -0.31232 | 0.264086 |
| -0.00182 | 0.087585 | 0.012322 | -0.14786 | 0.882454 | -0.02597 | 0.022329 | -0.02124 | -0.30285 | 0.26036 |
| -0.00197 | 0.087732 | 0.013634 | -0.14437 | 0.885212 | -0.02869 | 0.024754 | -0.02295 | -0.33453 | 0.288633 |
| -0.01138 | 0.097145 | 0.012426 | -0.91597 | 0.359682 | -0.03574 | 0.012973 | -0.13271 | -0.4167 | 0.151269 |
| -0.00679 | 0.092558 | 0.014095 | -0.48206 | 0.629761 | -0.03442 | 0.020831 | -0.07922 | -0.40134 | 0.242893 |
| -0.00525 | 0.091017 | 0.016272 | -0.32286 | 0.746801 | -0.03715 | 0.02664 | -0.06126 | -0.43314 | 0.31062 |
| 0.015052 | 0.070711 | 0.019876 | 0.757319 | 0.448859 | -0.0239 | 0.054009 | 0.17551 | -0.27872 | 0.629742 |

| id | nsnp.x | method.x | b.x | se.x | or.x | or_lci95.x | or_uci95.x | pvalue.x | nsnp.y |
|---|---|---|---|---|---|---|---|---|---|
| ebi-a-GC | 208 | Inverse va | -0.2558 | 0.089754 | 0.774295 | 0.649391 | 0.923223 | 0.004371 | 8 |
| ebi-a-GC | 208 | Inverse va | 0.181635 | 0.091438 | 1.199177 | 1.002419 | 1.434555 | 0.046986 | 3 |
| ebi-a-GC | 208 | Inverse va | 0.232269 | 0.087229 | 1.261459 | 1.063218 | 1.496662 | 0.00775 | 7 |
| ebi-a-GC | 208 | Inverse va | 0.187783 | 0.084116 | 1.206572 | 1.023181 | 1.422834 | 0.025586 | 12 |
| ebi-a-GC | 208 | Inverse va | 0.188945 | 0.089399 | 1.207974 | 1.013817 | 1.439315 | 0.034558 | 9 |
| ebi-a-GC | 208 | Inverse va | 0.27096 | 0.08846 | 1.311222 | 1.102497 | 1.559464 | 0.002191 | 34 |
| ebi-a-GC | 208 | Inverse va | 0.273571 | 0.089742 | 1.314651 | 1.102607 | 1.567473 | 0.0023 | 20 |
| ebi-a-GC | 208 | Inverse va | 0.326278 | 0.121852 | 1.385801 | 1.091385 | 1.75964 | 0.007414 | 16 |
| ebi-a-GC | 208 | Inverse va | 0.387171 | 0.089338 | 1.472809 | 1.236233 | 1.754658 | 1.47E-05 | 24 |
| ebi-a-GC | 208 | Inverse va | 0.29311 | 0.089928 | 1.340591 | 1.123952 | 1.598986 | 0.001117 | 24 |
| ebi-a-GC | 208 | Inverse va | 0.212759 | 0.089269 | 1.237087 | 1.038516 | 1.473626 | 0.017156 | 26 |
| ebi-a-GC | 208 | Inverse va | 0.243832 | 0.089169 | 1.276129 | 1.071501 | 1.519836 | 0.006248 | 33 |

| method.y | b.y | se.y | or.y | or_lci95.y | or_uci95.y | pvalue.y | beta_all | beta1 | beta2 |
|---|---|---|---|---|---|---|---|---|---|
| Inverse va | -0.05937 | 0.019601 | 0.942363 | 0.906846 | 0.97927 | 0.002456 | 0.63619 | -0.2558 | -0.05937 |
| Inverse va | 0.149661 | 0.049716 | 1.161441 | 1.053605 | 1.280312 | 0.00261 | 0.63619 | 0.181635 | 0.149661 |
| Inverse va | 0.072328 | 0.023552 | 1.075008 | 1.026512 | 1.125796 | 0.002133 | 0.63619 | 0.232269 | 0.072328 |
| Inverse va | 0.057755 | 0.022923 | 1.059455 | 1.012908 | 1.108141 | 0.011753 | 0.63619 | 0.187783 | 0.057755 |
| Inverse va | 0.091163 | 0.037375 | 1.095448 | 1.018068 | 1.178708 | 0.014723 | 0.63619 | 0.188945 | 0.091163 |
| Inverse va | 0.025804 | 0.011302 | 1.02614 | 1.00366 | 1.049124 | 0.022416 | 0.63619 | 0.27096 | 0.025804 |
| Inverse va | 0.039346 | 0.012342 | 1.04013 | 1.015271 | 1.065597 | 0.001432 | 0.63619 | 0.273571 | 0.039346 |
| Inverse va | 0.040166 | 0.010623 | 1.040984 | 1.019533 | 1.062886 | 0.000156 | 0.63619 | 0.326278 | 0.040166 |
| Inverse va | 0.042846 | 0.013383 | 1.043777 | 1.016753 | 1.071519 | 0.001367 | 0.63619 | 0.387171 | 0.042846 |
| Inverse va | 0.037616 | 0.013313 | 1.038332 | 1.011589 | 1.065783 | 0.004721 | 0.63619 | 0.29311 | 0.037616 |
| Inverse va | 0.049522 | 0.0118 | 1.050768 | 1.026746 | 1.075353 | 2.71E-05 | 0.63619 | 0.212759 | 0.049522 |
| Inverse va | 0.030011 | 0.01075 | 1.030466 | 1.008981 | 1.052408 | 0.005243 | 0.63619 | 0.243832 | 0.030011 |

| beta12 | beta_dir | se | Z | P | lci | uci | beta12_p | lci_p | uci_p |
|---|---|---|---|---|---|---|---|---|---|
| 0.015186 | 0.621004 | 0.022989 | 0.660574 | 0.508886 | -0.02987 | 0.060244 | 0.02387 | -0.04695 | 0.094694 |
| 0.027184 | 0.609006 | 0.018199 | 1.493699 | 0.135254 | -0.00849 | 0.062854 | 0.042729 | -0.01334 | 0.098797 |
| 0.0168 | 0.61939 | 0.020332 | 0.826265 | 0.408654 | -0.02305 | 0.05665 | 0.026407 | -0.03623 | 0.089046 |
| 0.010845 | 0.625344 | 0.015851 | 0.684207 | 0.493844 | -0.02022 | 0.041913 | 0.017047 | -0.03179 | 0.065882 |
| 0.017225 | 0.618965 | 0.017232 | 0.999597 | 0.317506 | -0.01655 | 0.050999 | 0.027075 | -0.02601 | 0.080163 |
| 0.006992 | 0.629198 | 0.023971 | 0.291685 | 0.770528 | -0.03999 | 0.053975 | 0.01099 | -0.06286 | 0.084841 |
| 0.010764 | 0.625426 | 0.024556 | 0.438347 | 0.661135 | -0.03737 | 0.058893 | 0.016919 | -0.05873 | 0.092571 |
| 0.013105 | 0.623084 | 0.03976 | 0.32961 | 0.741694 | -0.06482 | 0.091035 | 0.0206 | -0.10189 | 0.143094 |
| 0.016589 | 0.619601 | 0.034594 | 0.479528 | 0.631563 | -0.05122 | 0.084393 | 0.026075 | -0.0805 | 0.132654 |
| 0.011026 | 0.625164 | 0.026364 | 0.418214 | 0.675791 | -0.04065 | 0.062698 | 0.017331 | -0.06389 | 0.098553 |
| 0.010536 | 0.625653 | 0.019002 | 0.554485 | 0.579247 | -0.02671 | 0.04778 | 0.016561 | -0.04198 | 0.075103 |
| 0.007318 | 0.628872 | 0.021745 | 0.336526 | 0.736474 | -0.0353 | 0.049937 | 0.011502 | -0.05549 | 0.078494 |

**STROBE-MR checklist of recommended items to address in reports of Mendelian randomization studies**[1 2]

| Item No. | Section | | Checklist item | Page No. | Relevant text from manuscript |
|---|---|---|---|---|---|
| 1 | **TITLE and ABSTRACT** | | Indicate Mendelian randomization (MR) as the study's design in the title and/or the abstract if that is a main purpose of the study | Page 1-2 | Title, abstract/paragraph 2 |
| | **INTRODUCTION** | | | | |
| 2 | **Background** | | Explain the scientific background and rationale for the reported study. What is the exposure? Is a potential causal relationship between exposure and outcome plausible? Justify why MR is a helpful method to address the study question | Page 4 | Introduction/paragraph 4 |
| 3 | **Objectives** | | State specific objectives clearly, including pre-specified causal hypotheses (if any). State that MR is a method that, under specific assumptions, intends to estimate causal effects | Page 4 | Introduction/paragraph 4 |
| | **METHODS** | | | | |
| 4 | **Study design and data sources** | | Present key elements of the study design early in the article. Consider including a table listing sources of data for all phases of the study. For each data source contributing to the analysis, describe the following: | Page 5 | Methods/paragraph 1 |
| | | a) | Setting: Describe the study design and the underlying population, if possible. Describe the setting, locations, and relevant dates, including periods of recruitment, exposure, follow-up, and data collection, when available. | Page 6 | Methods/paragraph 4 |
| | | b) | Participants: Give the eligibility criteria, and the sources and methods of selection of participants. Report the sample size, and whether any power or sample size calculations were carried out prior to the main analysis | Page 6 | Methods/paragraph 5 |
| | | c) | Describe measurement, quality control and selection of genetic variants | Page 6-7 | Methods/paragraph 5 |
| | | d) | For each exposure, outcome, and other relevant variables, describe methods of assessment and diagnostic criteria for diseases | Page 6-7 | Methods/paragraph 5 |
| | | e) | Provide details of ethics committee approval and participant informed consent, if relevant | NA: The data utilized in this study have all been previously published, thus obviating the requirement for ethical approval and consent to participate. | NA |

| | | | | | |
|---|---|---|---|---|---|
| 5 | **Assumptions** | | Explicitly state the three core IV assumptions for the main analysis (relevance, independence and exclusion restriction) as well assumptions for any additional or sensitivity analysis | Page 6 | Methods/paragraph 1 |
| 6 | **Statistical methods: main analysis** | | Describe statistical methods and statistics used | Page 6-7 | Methods/paragraph 5 |
| | | a) | Describe how quantitative variables were handled in the analyses (i.e., scale, units, model) | Page 6-7 | Methods/paragraph 5 |
| | | b) | Describe how genetic variants were handled in the analyses and, if applicable, how their weights were selected | Page 6-7 | Methods/paragraph 5 |
| | | c) | Describe the MR estimator (e.g. two-stage least squares, Wald ratio) and related statistics. Detail the included covariates and, in case of two-sample MR, whether the same covariate set was used for adjustment in the two samples | Page 6-7 | Methods/paragraph 5 |
| | | d) | Explain how missing data were addressed | NA: Missing data codes are automatically filtered out of the analysis when it is performed, and this description is not presented in the main text. | NA |
| | | e) | If applicable, indicate how multiple testing was addressed | NA: There is no batch analysis in this paper, and the issue of multiple testing is not addressed | NA |
| 7 | **Assessment of assumptions** | | Describe any methods or prior knowledge used to assess the assumptions or justify their validity | Page 6-7 | Methods/paragraph 5 |
| 8 | **Sensitivity analyses and additional analyses** | | Describe any sensitivity analyses or additional analyses performed (e.g. comparison of effect estimates from different approaches, independent replication, bias analytic techniques, validation of instruments, simulations) | Page 6-7 | Methods/paragraph 5 |
| 9 | **Software and pre-registration** | | | | |
| | | a) | Name statistical software and package(s), including version and settings used | Page 6-7 | Methods/paragraph 5 |
| | | b) | State whether the study protocol and details were pre-registered (as well as when and where) | NA: The data used in the analysis of the article had | NA |

| | | | | |
|---|---|---|---|---|
| | | | been published prior to the analysis of the article, and the research protocol and detailed information were conceptualized and adapted during the course of the analysis, and were not pre-registered. | |
| | **RESULTS** | | | |
| 10 | **Descriptive data** | | | |
| | a) | Report the numbers of individuals at each stage of included studies and reasons for exclusion. Consider use of a flow diagram | Page 8 | Results/paragraph 3-4 |
| | b) | Report summary statistics for phenotypic exposure(s), outcome(s), and other relevant variables (e.g. means, SDs, proportions) | NA: This information is described in the databases used, so it is not shown in the article. | NA |
| | c) | If the data sources include meta-analyses of previous studies, provide the assessments of heterogeneity across these studies | NA: The data used in the analysis of the article is not derived from a meta-analysis. | NA |
| | d) | For two-sample MR:<br>i. Provide justification of the similarity of the genetic variant-exposure associations between the exposure and outcome samples<br>ii. Provide information on the number of individuals who overlap between the exposure and outcome studies | NA: Our exposures were obtained from European and Icelandic populations, while the outcome data came from Finnish participants. The samples did not overlap; therefore, the sample overlap rate was not calculated in the analyses. Additionally, we used SNPs with an F-statistic greater than 10 to ensure that the samples remained independent. | NA |
| 11 | **Main results** | | | |
| | a) | Report the associations between genetic variant and exposure, and between genetic variant and outcome, preferably on an interpretable scale | Page 8 | Results/paragraph 5 |

| | | | | | |
|---|---|---|---|---|---|
| | | b) | Report MR estimates of the relationship between exposure and outcome, and the measures of uncertainty from the MR analysis, on an interpretable scale, such as odds ratio or relative risk per SD difference | Page 8 | Results/paragraph 5 |
| | | c) | If relevant, consider translating estimates of relative risk into absolute risk for a meaningful time period | NA: Not relevant to this article. | NA |
| | | d) | Consider plots to visualize results (e.g. forest plot, scatterplot of associations between genetic variants and outcome versus between genetic variants and exposure) | Page 8,10 | Results/paragraph 5,11 |
| 12 | **Assessment of assumptions** | | | | |
| | | a) | Report the assessment of the validity of the assumptions | Page 8 | Results/paragraph 6 |
| | | b) | Report any additional statistics (e.g., assessments of heterogeneity across genetic variants, such as $I^2$, Q statistic or E-value) | Page 8 | Results/paragraph 6 |
| 13 | **Sensitivity analyses and additional analyses** | | | | |
| | | a) | Report any sensitivity analyses to assess the robustness of the main results to violations of the assumptions | Page 8 | Results/paragraph 6-7 |
| | | b) | Report results from other sensitivity analyses or additional analyses | Page 8 | Results/paragraph 6-7 |
| | | c) | Report any assessment of direction of causal relationship (e.g., bidirectional MR) | NA: available genetic instruments for COPD were not used to explore the reverse causality in this analysis. | NA |
| | | d) | When relevant, report and compare with estimates from non-MR analyses | N/A: There are no relevant large cohort studies or clinical trials. | NA |
| | | e) | Consider additional plots to visualize results (e.g., leave-one-out analyses) | Page 10 | Results/paragraph 10 |
| | **DISCUSSION** | | | | |
| 14 | **Key results** | | Summarize key results with reference to study objectives | Page 11 | Discussion/Paragraph 1 |
| 15 | **Limitations** | | Discuss limitations of the study, taking into account the validity of the IV assumptions, other sources of potential bias, and imprecision. Discuss | Page 14 | Discussion/Paragraph 7 |

| | | | | |
|---|---|---|---|---|
| | | both direction and magnitude of any potential bias and any efforts to address them | | |
| 16 | **Interpretation** | | | |
| | a) | Meaning: Give a cautious overall interpretation of results in the context of their limitations and in comparison with other studies | Page 15 | Discussion/Paragraph 7-8 |
| | b) | Mechanism: Discuss underlying biological mechanisms that could drive a potential causal relationship between the investigated exposure and the outcome, and whether the gene-environment equivalence assumption is reasonable. Use causal language carefully, clarifying that IV estimates may provide causal effects only under certain assumptions | Page 13 | Discussion/Paragraph 5 |
| | c) | Clinical relevance: Discuss whether the results have clinical or public policy relevance, and to what extent they inform effect sizes of possible interventions | NA: The study is about the relationship between OAF and COPD and does not involve clinical guidance on some treatments. | NA |
| 17 | **Generalizability** | Discuss the generalizability of the study results (a) to other populations, (b) across other exposure periods/timings, and (c) across other levels of exposure | Page 14 | Discussion/Paragraph 6-7 |
| | **OTHER INFORMATION** | | | |
| 18 | **Funding** | Describe sources of funding and the role of funders in the present study and, if applicable, sources of funding for the databases and original study or studies on which the present study is based | Page 16 | Funding |
| 19 | **Data and data sharing** | Provide the data used to perform all analyses or report where and how the data can be accessed, and reference these sources in the article. Provide the statistical code needed to reproduce the results in the article, or report whether the code is publicly accessible and if so, where | Page 16 | Data Availability |
| 20 | **Conflicts of Interest** | All authors should declare all potential conflicts of interest | Page 16 | Disclosure |

This checklist is copyrighted by the Equator Network under the Creative Commons Attribution 3.0 Unported (CC BY 3.0) license.

1. Skrivankova VW, Richmond RC, Woolf BAR, Yarmolinsky J, Davies NM, Swanson SA, et al. Strengthening the Reporting of Observational Studies in Epidemiology using Mendelian Randomization (STROBE-MR) Statement. JAMA. 2021;under review.

2. Skrivankova VW, Richmond RC, Woolf BAR, Davies NM, Swanson SA, VanderWeele TJ, et al. Strengthening the Reporting of Observational Studies in Epidemiology using Mendelian Randomisation (STROBE-MR): Explanation and Elaboration. BMJ. 2021;375:n2233.

© 2025 You F. et al.
